# Friedelane-Type Triterpenoids from *Maytenus quadrangulata* as Potential Anticancer Agents

**DOI:** 10.1021/acsomega.5c12356

**Published:** 2026-05-15

**Authors:** Mariana G. Aguilar, Sandy V. M. Quintão, Túlio R. Freitas, Samuel R. Sabina, Mateus S. M. Serafim, Jônatas S. Abrahão, Adriano P. Sabino, Daniel C. F. Soares, Raimundo M. Cabrera, Ignacio A. Jiménez, Isabel L. Bazzocchi, Grasiely F. Sousa, Lucienir P. Duarte

**Affiliations:** † Departamento de Química, 28114 Universidade Federal de Minas Gerais, 31270-901 Belo Horizonte-MG, Brazil; ‡ Departamento de Análises Clínicas e Toxicológicas, Faculdade de Farmácia, 28114 Universidade Federal de Minas Gerais, 31270-901 Belo Horizonte-MG, Brazil; § Departamento de Botánica, Ecología y Fisiología Vegetal, Facultad de Biología, 16749 Universidad de La Laguna, Avenida Astrofísico Francisco Sánchez 2, 38206 La Laguna, Tenerife, Spain; ∥ Instituto de Ciências Biológicas, 28114 Universidade Federal de Minas Gerais, 31270-901 Belo Horizonte-MG, Brazil; ⊥ Laboratório de Bioengenharia, Universidade Federal de Itajubá, 35903-087 Itabira-MG, Brazil; # Instituto Universitario de Bio-Orgánica Antonio González, Departamento de Química Orgánica, 16749 Universidad de La Laguna, Avenida Astrofísico Francisco Sánchez 2, 38206 La Laguna, Tenerife, Spain

## Abstract

*Maytenus quadrangulata* is a species endemic to the Atlantic Forest in Brazil. While the pharmacological potential of the *Maytenus* genus is well-documented, specific phytochemical studies on *M. quadrangulata* remain limited, although previous investigations have identified triterpenes with significant cytotoxic effects. In this study, five new acetylated triterpenoid derivatives, namely, friedelane-3α,29-diyl diacetate (**1**), friedelane-3α,11β-diyl diacetate (**2**), friedelane-3α,25-diyl diacetate (**3**), 3β-hydroxyfriedelane-24-yl acetate (**4**), and 11β-hydroxyfriedelane-3α-yl acetate (**5**), were obtained by acetylation of a triterpenoid mixture from the chloroform extract of *M. quadrangulata* leaves. Additionally, 12 known compounds were identified from this extract. The structural elucidation was performed using spectroscopic (IR, NMR) and spectrometric (MS) techniques. The cytotoxic activity of the isolated triterpenes was assessed on leukemia (K-562, THP-1), breast cancer (MDA-MB-231), and epithelial lung carcinoma (A549) cell lines. Among the tested compounds, compound **5** showed a more pronounced effect against the K-562 chronic myeloid leukemia cell line . Furthermore, all tested compounds exhibited moderate cytotoxic activity against the A549 epithelial lung carcinoma cell line. The antiviral potential of the isolated compounds was also evaluated against Zika virus (ZIKV), along with the activity against phytopathogenic fungi *Alternaria alternata*, *Botrytis cinerea*, and *Fusarium oxysporum*; however, no significant inhibitory effects were observed. Collectively, this study provides a detailed characterization of the bioactive triterpenoids from *M. quadrangulata*, reinforcing its relevance as a potential source of cytotoxic natural products.

## Graphical abstract

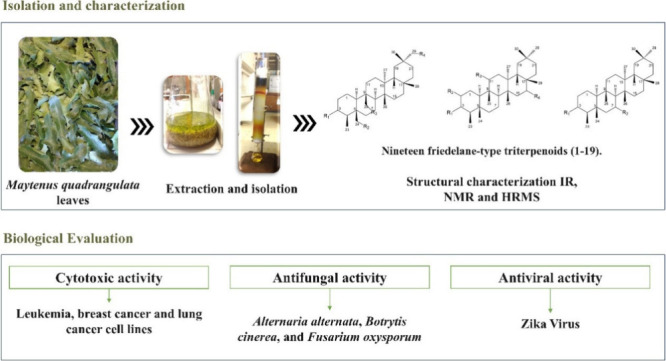

## Introduction

Terpenes represent one of the most widespread classes of metabolites among natural products, with over 20,000 identified substances. They display a broad diversity of chemical structures, comprising approximately 200 distinct skeletons, many of which are variations of a smaller number of common frameworks. Triterpenes, particularly pentacyclic ones, are widely distributed in plants, being found in leaves, roots, stems, and seeds, as well as coatings (similar to waxes) on fruits such as apples and in herbs like oregano, rosemary, lavender, and thyme.^1,2^

Triterpenes have demonstrated a broad spectrum of biological effects, including antiviral, antitumor, antifungal, hepatoprotective, cardioprotective, antioxidant, and anti-inflammatory activities.^3^ For instance, friedelan-3-one (friedelin) exhibits antiviral, hepatoprotective, anti-inflammatory (*in vivo*), and antileukemic effects,^4–6^ and pentacyclic triterpenoids, especially urs-12-en-3β-ol and friedelan-3β-ol, show cytotoxicity on leukemia cell lines (THP-1 and K562), with selectivity indexes comparable to commonly used chemotherapeutic agents.^7^ Another example is methyl bardoxolone, a synthetic derivative of the triterpenoid oleanolic acid, which has undergone two phase III clinical trials for the treatment of chronic kidney disease and pulmonary hypertension.^1,8,9^

Pentacyclic triterpenes are frequently isolated from species of the Celastraceae family, particularly from species of the genus *Maytenus*. In a comprehensive phytochemical investigation of 20 *Maytenus* species, 506 distinct compounds were identified, of which 250 were triterpenes, representing nearly half of the total metabolites reported.^10^ In addition, the biological assessment of different extracts and purified compounds has revealed anti-inflammatory, antiulcerogenic, antioxidant, antibacterial, and analgesic effects. Despite this chemical and biological diversity, most studies have focused on a small number of species.^10^

A previous study on the hexane extract of *M. quadrangulata* leaves led to isolation of triterpenes with high cytotoxic potential. The hexane extract and triterpenes 3,4-*seco*-3,11β-epoxyfriedel-4­(23)-en-3β-ol, friedelan-3α,11β-diol, and 11β-hydroxyfriedelan-3-one exhibited strong cytotoxicity and selectivity against leukemia (THP-1 and K562) and breast cancer (MDA-MB-231) cell lines compared to the positive controls.^11^ Moreover, ethyl acetate extracts from the leaves and branches of *M. quadrangulata* have been shown to exhibit *in vitro* antiviral activity against an arthropod-borne virus, Mayaro virus (MAYV).^12^ These findings prompted our broader investigation of *M. quadrangulata* leaves, with phytochemical study of the chloroform extract aimed toward the discovery new compounds with biological or therapeutic potential.

In this study, the phytochemical analysis of the chloroform extract from *M. quadrangulata* leaves (LCE) led to the identification of five new acetate triterpenoid derivatives: friedelane-3α,29-diyl diacetate (**1**), friedelane-3α,11β-diyl diacetate (**2**), friedelane-3α,25-diyl diacetate (**3**), 3β-hydroxyfriedelane-24-yl acetate (**4**), and 11β-hydroxyfriedelane-3α-yl acetate (**5**). Additionally, 12 other triterpenes previously described in the literature were identified: friedelan-3-one (**6**), friedelan-3β-ol (**7**), friedelan-3α-ol (**8**), friedelane-3,7-dione (**9**), 11β-hydroxyfriedelan-3-one (**10**), 3β-hydroxyfriedelan-7-one (**11**), 3α-hydroxyfriedelan-7-one (**12**), friedelane-2α,3α-diol (**13**), friedelane-3α,11β-diol (**14**), friedelane-3α,25-diol (**15**), friedelane-3α-yl acetate (**16**), friedelane-3β,24-diol (**17**), and two mixtures of triterpenes: friedelane-3α,16β-diol (**18**) and **13**, and friedelane-3β,24-diyl diacetate (**19**) and **3** (Figure 1). The biological relevance of these compounds was evaluated *in vitro* through comprehensive assessments of their antitumor, antiviral, and antifungal potential, aiming to identify novel bioactive molecules with therapeutic applications.

**Figure 1:**
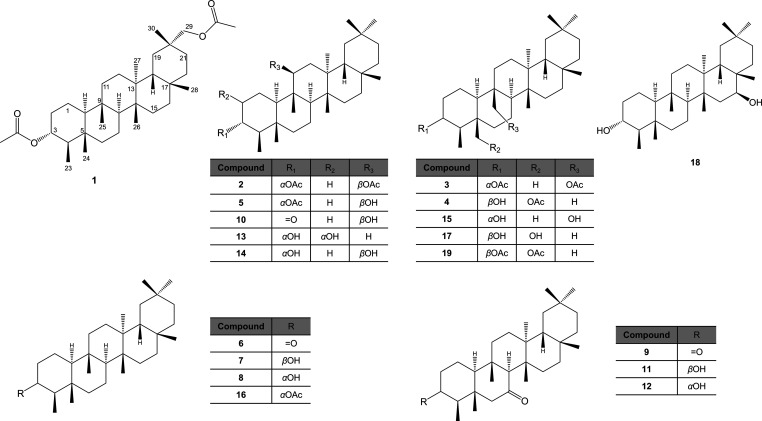
Chemical structure of isolated and derivatives triterpenoids from the chloroform extract of *M. quadrangulata* leaves.

## Results and Discussion

### Structure Elucidation

During the phytochemical study of the chloroform extract from *M. quadrangulata* leaves, 12 known triterpenes were directly characterized (**6–18**). Moreover, five new triterpene derivatives (**1–5**) in the form of acetates were obtained by acetylation of mixtures followed by subsequent isolation. In addition, two inseparable mixtures were characterized: (**13** and **18**) and (**3** and **19**). The structures of the new compounds were deduced as described below.

The acetate derivative, designated as compound **1**, was isolated as a white crystalline solid with a melting point of 195–196 °C. Its molecular formula, C_34_H_56_O_4_, was established by HR-ESI-MS (*m*/*z*: 551.4066 [M + Na]^+^, calcd. 551.4071). In the IR spectrum, a stretching band of the C=O bond was observed at 1738 cm^–1^, and bands at 1246 cm^–1^ and 1034 cm^–1^ were related to the stretching of the C–O bond of esters group. The ^1^H NMR spectrum (Table 1) showed six singlets at δ_H_ 0.82, 0.83, 0.99, 1.01, 1.04, and 1.20 corresponding to six methyl groups, a doublet at δ_H_ 0.76, (*J* = 6.7 Hz), and two singlets at δ_H_ 2.07 and δ_H_ 2.03 characteristic of the methyl groups of acetate moieties. Moreover, the spectrum also displayed signals attributable to an oxymethine proton at δ_H_ 4.63, as a triplet of doublets (*J* = 10.9, 5.0 Hz), together with a singlet at δ_H_ 3.74 corresponding to two equivalent oxymethylene protons. These resonances are characteristic of secondary and primary acetate moieties. These data provide evidence that compound **1** belongs to the pentacyclic triterpene with a friedelane skeleton.

**Table 1:** ^1^H NMR Spectroscopic Data of Compounds **1–4** ^a^ and **15** ^b^

H	**1**	**2**	**3**	**4**	**15**
1	1.36; 1.59	1.99	1.71; 1.74	1.46; 1.62	1.67
2	1.29 α; 2.07 β	1.45 α; 2.02 β	1.14 α; 2.03 β	2.02; 2.05	1.21 α; 2.08 β
3	4.63 β td (10.9, 5.0)	4.57 β dt (11.2, 4.9)	4.58 β	3.76 α	3.35 β td (10.7, 4.8)
4	1.28 α	1.26 α	1.30 α	1.40 α	1.10 α
6	1.05 α; 1.76 β	1.04 α; 1.76 β	1.12 α; 1.88 β	1.60; 1.97	1.08 α; 1.82 β
7	1.41	1.45	1.33 *β;* 1.43 α	1.42	1.41
8	1.28 α	1.36 α	1.45 α	1.32 α	1.42 α
10	0.95 α	1.15 α	1.09 α	1.07 α	1.05 α
11	1.13; 1.38	4.80 dd (11.2, 4.7)	1.34	1.34; 1.50	1.09; 2.01
12	1.24; 1.35	1.28 β; 1.62 α	1.26; 1.48	1.32	1.32 β; 1.43 α
15	1.28; 1.51	1.52	1.33	1.25; 1.46	1.26; 1.50
16	1.33 α; 1.58 β	1.10; 1.34	1.55	1.34; 1.21	1.34 α; 1.55 β
18	1.59 β	1.52 β	1.54 β	1.54 β	1.54 β
19	1.18 α; 1.45 β	1.34	1.18 α; 1.33 β	1.11; 1.36	1.18 α; 1.36 β
21	1.39	1.22; 1.45	1.26; 1.48	1.25; 1.48	1.26; 1.46
22	0.97 β; 1.44 α	0.93 β; 1.44 α	0.92 β; 1.48 α	0.91; 1.48	0.92 β; 1.49 α
23	0.76 d (6.7)	0.75 d (6.7)	0.78 d (6.7)	1.05 d (7.4)	0.95 d (6.6)
24	0.83 s	0.84 s	0.88 s	4.37 d (11.9); 4.62 d (11.9)	0.89 s
25	0.82 s	0.98 s	4.15 d (11.7); 4.53 d (11.7)	0.89 s	3.83 d (11.8); 3.93 d (11.8)
26	1.01 s	1.02 s	1.01 s	1.01 s	1.01 s
27	0.99 s	1.13 s	1.03 s	1.00 s	1.03 s
28	1.20 s	1.16 s	1.18 s	1.17 s	1.16 s
29	3.74 s	0.94 s	0.94 s	0.94 s	0.94 s
30	1.04 s	0.98 s	0.92 s	0.99 s	0.98 s
2′	2.03 s	1.98 s	2.02 s	2.08 s	
4′	2.07 s	1.94 s	2.03 s		

a Spectra were recorded in CDCl_3_ at 400 MHz.

b Spectra were recorded in CDCl_3_ at 600 MHz. *J* in Hz in parentheses. Signals without multiplicity assignments were overlapping resonances deduced by HSQC experiments.

The ^13^C NMR spectrum (Table 2) revealed 30 carbon signals for a triterpene unit and four carbon signals for two acetate groups, which were assigned by HSQC analysis as nine methyls, 11 methylenes, four methines, eight nonprotonated carbons, one oxymethine, one oxymethylene and two carboxyl carbons. The two *sp* ^3^ carbons observed at δ_C_ 75.2 (CH) and 75.2 (CH_2_) in the ^13^C NMR spectrum confirmed the secondary and primary acetate moieties on the friedelane skeleton. Moreover, the ^13^C NMR data for **1** are close to those of friedelan-3β,29-diol.^13^ The regiosubstitution of **1** was determined by a HMBC experiment, showing three bond correlations between the proton signal at δ_H_ 3.74 (H-29) and the resonances at δ_C_ 30.0 (C-19), δ_C_ 31.7 (C-20), δ_C_ 28.1 (C-21), δ_C_ 26.3 (C-30), and δ_C_ 171.6 (C-3′), establishing the presence of the acetyl group at the C-29 position. Moreover, the acetyl group at C-3 was determined by correlations between the proton signal at δ_H_ 4.63 (H-3) and the signals at δ_C_ 32.5 (C-2), 50.0 (C-4), 9.9 (C-23), and 171.0 (C-1′), along with the correlation of C-1′ with the signal at δ_H_ 2.03 (H-2′).

**Table 2:** ^13^C NMR Spectroscopic Data of Compounds **1–5** ^a^ and **15** ^b^

C	**1**		**2**		**3**		**4**		**5**		**15**	
1	CH_2_	19.3	CH_2_	21.5	CH_2_	21.9	CH_2_	16.0	CH_2_	22.3	CH_2_	22.1
2	CH_2_	32.5	CH_2_	32.6	CH_2_	34.0	CH_2_	37.0	CH_2_	32.8	CH_2_	37.8
3	CH	75.2	CH	74.4	CH	75.3	CH	71.8	CH	75.0	CH	71.8
4	CH	50.0	CH	50.0	CH	50.7	CH	49.5	CH	50.2	CH	53.7
5	C	38.3	C	39.1	C	38.6	C	30.0	C	39.2	C	37.9
6	CH_2_	41.3	CH_2_	41.1	CH_2_	41.8	CH_2_	35.3^c^	CH_2_	41.4	CH_2_	42.0
7	CH_2_	17.9	CH_2_	17.5	CH_2_	17.8	CH_2_	18.0	CH_2_	17.8	CH_2_	17.5
8	CH	53.2	CH	52.9	CH	53.8	CH	53.4	CH	52.7	CH	53.6
9	C	37.0	C	42.3	C	40.3	C	38.4	C	43.8	C	41.7
10	CH	59.9	CH	59.8	CH	61.0	CH	61.4	CH	60.7	CH	61.4
11	CH_2_	35.5	CH	80.2	CH_2_	30.3	CH_2_	36.0	CH	77.9	CH_2_	30.1
12	CH_2_	30.6	CH_2_	36.9	CH_2_	32.8	CH_2_	30.7	CH_2_	42.2	CH_2_	31.5
13	C	39.9	C	40.9	C	39.8	C	39.7	C	38.4	C	39.6
14	C	38.4	C	38.2	C	38.0	C	40.7	C	41.2	C	38.2
15	CH_2_	32.7	CH_2_	32.4	CH_2_	30.7	CH_2_	32.2	CH_2_	32.5	CH_2_	32.6
16	CH_2_	35.9	CH_2_	35.9	CH_2_	36.1	CH_2_	35.4^c^	CH_2_	36.0	CH_2_	36.0
17	C	30.4	C	30.1	C	30.1	C	29.7	C	30.1	C	29.9
18	CH	41.9	CH	42.5	CH	42.7	CH	42.8	CH	42.7	CH	42.7
19	CH_2_	30.0	CH_2_	35.4	CH_2_	35.4	CH_2_	35.8	CH_2_	35.5	CH_2_	35.2
20	C	31.7	C	28.2	C	28.3	C	28.2	C	28.3	C	28.1
21	CH_2_	28.1	CH_2_	32.7	CH_2_	32.8	CH_2_	32.8	CH_2_	33	CH_2_	32.8
22	CH_2_	39.2	CH_2_	39.3	CH_2_	39.4	CH_2_	39.3	CH_2_	39.3	CH_2_	39.2
23	CH_3_	9.9	CH_3_	9.8	CH_3_	10.2	CH_3_	13.2	CH_3_	10.1	CH_3_	10.1
24	CH_3_	14.5	CH_3_	14.7	CH_3_	14.5	CH_2_	66.0	CH_3_	14.8	CH_3_	14.6
25	CH_3_	18.1	CH_3_	14.4	CH_2_	65.5	CH_3_	18.0	CH_3_	13.2	CH_2_	62.9
26	CH_3_	20.5	CH_3_	20.1	CH_3_	20.5	CH_3_	18.7	CH_3_	20.1	CH_3_	19.9
27	CH_3_	18.4	CH_3_	19.0	CH_3_	18.8	CH_3_	20.1	CH_3_	19.6	CH_3_	18.6
28	CH_3_	32.1	CH_3_	31.9	CH_3_	32.2	CH_3_	31.8	CH_3_	32.1	CH_3_	32.1
29	CH_2_	75.2	CH_3_	35.1	CH_3_	35.2	CH_3_	35.0	CH_3_	35.1	CH_3_	35.0
30	CH_3_	26.3	CH_3_	31.5	CH_3_	31.8	CH_3_	32.1	CH_3_	31.8	CH_3_	31.7
1′	C	171.0	C	171.0	C	171.1	C	170.6	C	171.1		
2′	CH_3_	21.4	CH_3_	21.3	CH_3_	21.4	CH_3_	21.3	CH_3_	21.5		
3′	C	171.6	C	170.8	C	171.3						
4′	CH_3_	21.0	CH_3_	22.0	CH_3_	21.4						

a Spectra were recorded in CDCl_3_ at 400 MHz.

b Spectra were recorded in CDCl_3_ at 600 MHz.

c The assignments may be interchanged. Data were based on DEPT, HSQC, and HMBC experiments.

The relative configuration of **1** was established on the basis of the coupling constants and confirmed by a NOESY experiment. Therefore, the *J*_2α,3β_ (10.9 Hz), *J*_3β,4α_ (10.9 Hz), and *J*_2β,3β_ (5.0 Hz) values indicated a *trans* and *cis* relationship between H-2/H-3, H-3/H-4, and H-2/H-3, respectively. Moreover, the NOESY experiment showed correlations of H-3 and H-23, H-24, and H-2β, confirming the presence of H-3 in the β-position and the acetate group in the α-position. Furthermore, correlations between H-29 and H-19*a*, H-21*a*, H-27, and H-30, as well as correlations between H-18 and H-26, H-28, and H-30, confirm the presence of the acetate group at C-29, and it is possible to propose the chair conformation for the E-ring of compound **1**. This evidence allowed us to established the structure of **1** as friedelane-3α,29-diyl diacetate.

A literature review revealed no reports of the triterpene friedelan-3α,29-diol, originally present in the leaf extract of *M. quadrangulata*. In addition, no reports were found regarding the acetylated compound **1**.

Compound **2** was obtained as a white crystalline solid with a melting point of 241–242 °C. The molecular formula, C_34_H_56_O_4_, was established by HR-ESI-MS (*m*/*z*: 551.4069 [M + Na]^+^, calcd. 551.4071). In the IR spectrum, bands at 1736, 1250, and 1022 cm^–1^ were observed, corresponding to the stretching of the C=O bond and the C–O ester bonds. The ^1^H and ^13^C NMR data (Tables 1 and 2) revealed that compound **2** is a triterpene possessing a friedelane-type skeleton with two acetate groups. The ^1^H NMR spectrum showed signals for seven methyl groups as singlets at δ_H_ 0.84, 0.94, 0.98, 0.98, 1.02, 1.13, and 1.16, along with a doublet at δ_H_ 0.75 (*J* = 6.7 Hz), characteristic of the triterpene unit. Additionally, the ^1^H NMR spectrum exhibited signals for two oxymethine protons at δ_H_ 4.80 (doublet of doublets, *J* = 11.2 and 4.7 Hz) and δ_H_ 4.57 (triplet of doublets *J* = 11.2 and 4.9 Hz), along with two singlets δ_H_ 1.94 (3H) and δ_H_ 1.98 (3H), belonging to the acetate groups. Moreover, in the ^1^H–^1^H COSY spectrum, H-3 (δ_H_ 4.57) showed cross-peaks with H-2 (δ_H_ 2.02 and 1.45) and H-4 (δ_H_ 1.26), and H-11­(δ_H_ 4.80) showed cross-peaks with H-12 (δ_H_ 1.62 and 1.28).

The ^13^C NMR spectrum revealed 30 carbon signals for a triterpene unit and four carbon signals for two acetate groups. The HSQC spectrum disclosed the correlations of all protons with their directly bonded carbon partners and showed signals for ten methyls, ten methylenes, four methines, six nonprotonated carbons, two oxymethine, and two carboxyl carbons. The above data for **2** were comparable to those reported for friedelane-3α,11β-diol (**14**).^11^ The substitution patterns of **2** were determined by a HMBC experiment, showing three-bond correlations between the carboxyl signals of the acetate group at δ_C_ 171.0 (C-1′) and 170.8 (C-3′) and the resonance at δ_H_ 4.57 (H-3) and 4.48 (H-11), respectively. Moreover, correlations were observed from H-23 to C-3, C-4, and C-5, and H-25 to C-8, C-9, C-10, and C-11. The relative configuration of **2** was established on the basis of the coupling constants and confirmed by a NOESY experiment, correlations between H-11 and H-27 (δ_H_ 1.13) and H-12α (δ_H_ 1.62) confirmed the presence of the acetyl group at C-11 in the β-position and H-11 in the α position. Besides, correlations between H-3 and H-2β (δ_H_ 2.02), H-23 (δ_H_ 0.75), and H-24 (δ_H_ 0.84), confirming the presence of the acetyl group at C-3 in the α position. All these data and comparison with those found in the literature for friedelane-3α,11β-diol (**14**)^11^ established the structure of **2** as friedelane-3α,11β-diyl diacetate. Compound **2**, a newly identified triterpene, was derived from compound **14**, which occurs naturally in the LCE extract

Due to the close structural resemblance and nearly identical physicochemical properties of compounds **3** and **19**, separation of the original mixture was not feasible. To overcome this, the mixture containing these compounds was subjected to acetylation, a strategy that had previously facilitated the separation of similar compounds in this study. Unfortunately, the resulting acetylated mixture also could not be separated, likely due to the negligible effect of acetylation on their chromatographic behavior. As a result, the identification of both compounds was carried out directly from the mixture using mass spectrometry and complementary spectroscopic methods. Their molecular formula, C_34_H_56_O_4_, was established by HR-ESI-MS (*m*/*z*: 551.4069 [M + Na]^+^, calcd. 551.4071). The IR spectrum displayed a strong absorption band at 1738 cm^–1^, characteristic of the C=O stretching vibration of ester carbonyl groups. Additionally, absorption bands at 1244 cm^–1^ and 1026 cm^–1^ were assigned to C–O stretching vibrations, consistent with the presence of ester functionalities.

Spectroscopic analyses based on one-dimensional ^1^H NMR (Table 1) and ^13^C NMR (Table 2) data evidenced characteristic resonances attributable to two triterpenes with a friedelane skeleton. The ^1^H NMR spectrum exhibited 14 methyl signals appearing as singlets and two methyl signals as doublets. In addition, the spectrum showed signals for two oxymethine protons as two multiplet at δ_H_ 4.98 and 4.58 ppm and two oxymethylene groups as four doublets at δ_H_ 4.65 and 4.43 ppm (*J* = 12.0 Hz), and δ_H_ 4.53 and 4.15 ppm (*J* = 11.7 Hz), as well as three singlets at δ_H_ 2.04 (6H), 2.03 (3H), and 2.02 ppm (3H), which were assigned to the methyl protons of acetate moieties. In the ^13^C NMR spectrum, a total of 65 carbon signals were observed, several displaying increased intensity, which is indicative of signal overlap. This observation supports the presence of a mixture of two structurally related triterpenes.

The presence of compound **3** in the mixture was confirmed by a detailed analysis of 1D and 2D NMR spectra and by comparison of the NMR data with those of friedelane-3β,24-diyl diacetate (**19**)^14^ and friedelane-3α,25-diol (**15**).^15^ For compound **3**, H-3 correlated with the signal at δ_C_ 75.3 (C-3) in the HSQC spectrum, as well as with C-2 (δ_C_ 34.0), C-4 (δ_C_ 50.7), C-23 (δ_C_ 10.2), and C-1′ (δ_C_ 171.1) in the HMBC spectrum. In the HSQC spectrum, correlations between the signal of C-25 (δ_C_ 65.5) and the doublets referring to H-25 (δ_H_ 4.53, *J* = 11.7 Hz, and δ_H_ 4.15, *J* = 11.7 Hz) were observed, in turn, H-25 correlated with C-8 (δ_C_ 53.8), C-9 (δ_C_ 40.3), C-10 (δ_C_ 61.0), C-11 (δ_C_ 30.3), and C-3′ (δ_C_ 171.3) in the HMBC spectrum, confirming the presence of the acetyl group at C-25. In the NOESY spectrum, correlations between H-3 and H-2β (δ_H_ 2.03), H-23, and H-24 were observed, indicating that H-3 is in the β-position and the acetyl group is in the α-position. Compound **3** has not yet been reported in the literature, while the triterpene friedelane-3α,25-diol (**15**), present in the leaf extract of *M. quadrangulata* and serving as the precursor to compound **3**, was previously reported as a derivative obtained through the reduction of 25-hydroxyfriedelan-3-one.^15^

Compound **4** was obtained as a white crystalline solid with melting point of 185–187 °C. The molecular formula, C_32_H_54_O_3_, was established by HR-ESI-MS (*m*/*z*: 509.3960 [M + Na]^+^, calcd. 509.3965). The IR spectrum showed characteristic absorption band for hydroxy (3464 cm^–1^) and ester (1708 cm^–1^, 1260 cm^–1^, and 1038 cm^–1^). In the ^1^H NMR spectrum (Table 1), six methyl groups were observed as singlets at δ_H_ 0.89, 0.94, 0.99, 1.00, 1.01 and 1.17, along with one methyl group as a doublet at δ_H_ 1.05 (*J* = 7.4 Hz), a oxymethine group as a multiplet at δ_H_ 3.76, a oxymethylene group as two doublets δ_H_ 4.37 (*J* = 12.0 Hz) and δ_H_ 4.62 (*J* = 12.0 Hz), and the methyl group as a singlet at 2.08, assignable to the acetate moiety. All these data were confirmed by the ^13^C NMR (Table 2). Comparing the NMR data with those of the friedelane-3β,24-diyl diacetate (**19**) indicated the main difference is the presence of a secondary alcohol in compound **4** instead of an acetate group in compound **19**. The comparison of the chemical shift of H-3 (δ_H_ 4.99) and H-24 (δ_H_ 4.40, 4.60) in the ^1^H NMR spectrum of compound **19** with those of compound **4**, H-3 (δ_H_ 3.76) and H-24 (δ_H_ 4.37, 4.62) showed a upfield shift only for H-3 in compound **4**, and the presence of a secondary alcohol at C-3 and an acetate group at C-24 was confirmed, thereby unambiguously establishing the identity of compound **4** as 3β-hydroxyfriedelane-24-yl acetate. Compound **4**, a new triterpene, was obtained through the partial acetylation of compound **17**, which is biosynthesized by *M. quadrangulata*. Although compound **17** was not directly isolated from the chloroform extract of *M. quadrangulata* leaves, it was prepared by the hydrolysis of compound **4**.

Compound **5** was obtained as a white crystalline solid with a melting point of 230–232 °C. The IR spectrum suggested that it contained a hydroxyl group (3532 cm^–1^), ester function (1736 cm^–1^) and band for C–O bond (1250 cm^–1^and 1024 cm^–1^). ^1^H NMR spectrum were those corresponding to seven methyl groups, six of them as singlets at δ_H_ 0.85, 0.88, 0.95, 0.99 (6H), 1.07, and 1.17 and one doublet δ_H_ 0.76 (*J* = 6.7 Hz), as well as signals for two oxymethine protons at δ_H_ 4.63 (triplet of doublets, *J* = 11.1 and 4.9 Hz), and δ_H_ 3.59 (doublet of doublets *J* = 11.3 and 4.7 Hz), along with a singlet at δ_H_ 2.02 (3H), belonging to two oxymethine protons and one acetate group, which were confirmed by the ^13^C NMR data (Table 2). Its NMR data showed that it had similar features to friedelane-3α,11β-diyl diacetate (**2**), with the only difference being the replacement of the acetate group at C-11 in compound **2** by a secondary alcohol in compound **5**. This was observed by comparing the chemical shift of H-3 (δ_H_ 4.57) and H-11 (δ_H_ 4.57) of the friedelane-3α,11β-diyl diacetate (**2**) with those of the compound **5** (H-3, δ_H_ 4.63 and H-11, δ_H_ 3.59). A downfield shift was noted for H-3, whereas only H-11 exhibited an upfield shift in compound **5**, thus unequivocally confirming the identity of the compound **5**, 11β-hydroxyfriedelane-3α-yl acetate. Compound **5**, a new triterpene, was synthesized through acetylation of compound **14**, which was isolated during this phytochemical study from the chloroform extract of *M. quadrangulata* leaves (LCE).

The structure of compound **15** was determined as friedelane-3α,25-diol by HR-ESI-MS data, ^1^H and ^13^C NMR studies (Tables 1 and 2), 2D ^1^H–^1^H, and ^1^H–^13^C correlations including COSY, NOESY, HSQC, and HMBC experiments, and comparison with data in the literature for the semisynthetic triterpene friedelane-3α,25-diol.^15^ However, complete ^1^H and ^13^C NMR data have not been previously reported. Therefore, a detailed analysis of the 1D and 2D NMR spectra was carried out to assign the chemical shift positions in the structure.

The chemical structures of compounds **6**–**14** and **16**–**19** were established through a comparison of ^1^H and ^13^C NMR data (Table S1) with literature data and identified as friedelan-3-one (**6**),^16^ friedelan-3β-ol (**7**),^17^ friedelan-3α-ol (**8**),^17^ friedelane-3,7-dione (**9**),^18^ 11β-hydroxyfriedelan-3-one (**10**),^19^ 3β-hydroxyfriedelan-7-one (**11**),^11^ 3α-hydroxyfriedelan-7-one (**12**),^11^ friedelane-2α,3α-diol (**13**),^18^ friedelane-3α,11β-diol (**14**),^11^ friedelane-3α-yl acetate (**16**),^20^ friedelane-3β,24-diol (**17**),^14^ friedelane-3α,16β-diol (**18**),^21^ and friedelane-3β,24-diyl diacetate (**19**).^14^ To the best of our knowledge, compound **18** was isolated for the first time from species of the Celastraceae family, and compound **15** was obtained for the first time from a plant species, with its complete ^1^H and ^13^C NMR data reported here. Similarly, compounds **1**–**5** are herein reported for the first time, whereas **13**, **15**, **18**, and **19** were first described for *M. quadrangulata*, and compounds **13**–**18** were described for the genus *Maytenus*.

### Biological Activity

#### Cytotoxic Activity Evaluation

##### Antiproliferative Activity in Leukemia and Human Breast Adenocarcinoma Cell Lines

The cytotoxic activity of compounds **1, 2, 4, 5**, and **15–17** was evaluated against the following cell lines: acute monocytic leukemia (THP-1), chronic myeloid leukemia (K-562), breast adenocarcinoma (MDA-MB-231), and a nontumoral lung fibroblast (WI-26 VA4) (Table 3). The nontumoral cell line was used to calculate the selectivity index (SI), which measures a compound’s preferential cytotoxicity toward cancer cells compared to normal cells. The SI is calculated as the ratio of the IC_50_ value (half-maximal inhibitory concentration) for the nontumoral cell line to the IC_50_ value for each cancer cell line. A SI value greater than 1.0 indicates higher efficacy against tumor cells relative to normal cells.^22^ Commercial anticancer agents imatinib, cytarabine, and doxorubicin were used as positive controls. The remaining compounds were not tested due to their limited availability or because their cytotoxic activities had already been reported in the literature.^11^

**Table 3:** Cytotoxic Activity (IC_50_, μM) and Selectivity Index (SI) of Compounds **1**, **2**, **4**, **5**, and **15–17** on Leukemia (THP-1, K-562), Breast Cancer (MDA-MB-231), and Non-Tumoral (WI-26 VA4) Cell Lines^a^

	THP-1^d^			K-562^e^	MDA-MB-231^f^		WI-26 VA4^g^
Compounds	IC_50_ (μM)^b^	SI	IC_50_ (μM)^b^	SI	IC_50_ (μM)^b^	SI	IC_50_ (μM)^b^
**1**	210.5 ± 8.9	ND	66.5 ± 5.4	ND	>300	ND	>300
**2**	230.4 ± 9.1	1.0	74.2 ± 6.3	3.3	143.5 ± 8.3	1.7	244.5 ± 10.8
**4**	81.3 ± 8.8	1.3	58.9 ± 4.5	1.8	72.5 ± 7.9	1.4	105.5 ± 10.3
**5**	120.8 ± 7.1	0.7	28.9 ± 3.6	3.1	52.9 ± 4.2	1.7	88.9 ± 6.0
**15**	245.6 ± 12.5	0.3	45.9 ± 4.8	1.5	61.3 ± 6.5	1.2	70.9 ± 5.6
**16**	>300		>300		>300		>300
**17**	>300		35.4 ± 5.6		>300		>300
**Imatinib** ^c^			34.6 ± 4.2				
**Citarabine** ^c^	40.7 ± 4.4						
**Doxorubicin** ^c^					2.3 ± 0.5		1.9 ± 0.9

a IC_50_, the minimum inhibitory concentration that resulted in 50% cell viability; SI, selectivity index.

b IC_50_ values are presented as means ± standard deviation (SD) of two independent experiments.

c Positive controls.

d THP-1, acute monocytic leukemia cell line.

e K-562, chronic myeloid leukemia cell line.

f MDA-MB-231, breast adenocarcinoma cell line.

g WI-26 VA4, nontumoral lung fibroblast cell line. Empty entries correspond to undetermined assays.

The tested compounds exhibited IC_50_ values ranging from 28.9 μM to over 300 μM against the leukemia cell lines THP-1 and K-562. The most favorable results were observed on the K-562 cell line, with IC_50_ values ranged from 28.9 μM to 74.2 μM, and selectivity indexes between 1.5 to 3.3. For the breast adenocarcinoma cell line MDA-MB-231, compounds **4**, **5**, and **15** showed moderate activity (IC_50_ values from 52.9 to 72.5 μM) with selectivity indexes ranging from 1.2 to 1.7.

These results are consistent with previous reports indicating that triterpenes display cytotoxic activity against cancer cell lines.^7,23–25^ In previous studies, the compound friedelane-3α,11β-diol (**14)** was reported to exhibit high cytotoxicity and selectivity against THP-1, K562, and MDA-MB-231 cell lines, with IC_50_ values below 30 μM and selectivity indexes above four.^11^ Therefore, it can be inferred that the presence of acetyl groups in compounds **2** (friedelane-3α,11β-diyl diacetate) and **5** (11β-hydroxyfriedelane-3α-yl acetate) may be responsible for the reduction in activity. This effect could be attributed to the increased lipophilicity of the compounds and the concomitant absence of hydrogen bonding capacity, factors which may interfere with their bioactivity. However, further studies, including mechanistic assays such as apoptosis induction, cell cycle analysis, and molecular target identification, are required to better elucidate the mechanisms underlying the observed cytotoxic effects.

##### Antiproliferative Activity in Lung Carcinoma Epithelial Cell Line

The cytotoxicity of compounds **2**, **4**, **5**, **8**, **10**, **12**, **14**, **16**, and **17** and the triterpene 3,4-*seco*-friedelan-3,11-olide (**TTPC1**), previously isolated from the hexane extract of *M. quadrangulata* leaves,^11^ was evaluated in lung carcinoma epithelial cells (A549). The remaining compounds were excluded from testing either due to their limited quantities or because their cytotoxic activities have already been documented in the literature.^23^ The IC_50_ data obtained are available in Table 4. All tested compounds revealed significant cytotoxic profiles against the tested cell line, with IC_50_ values ranging from 13.2 μM to 16.8 μM. However, despite the structural differences between the compounds, no significant variations in cytotoxic activity were observed (*p* > 0.05).

**Table 4:** Cytotoxic Activity (IC_50_, μM) of Compounds **2**, **4**, **5**, 8, **10**, **12**, **14**, **16**,**17**, and Triterpene (**TTPC1**) on a Carcinoma Epithelial Cell Line (A549)^a^

Compounds	IC_50_(μM)^b^
**2**	13.2 ± 0.4
**4**	14.6 ± 0.5
**5**	15.0 ± 0.5
**8**	16.4 ± 0.5
**10**	16.8 ± 0.7
**12**	16.7 ± 0.5
**14**	16.8 ± 0.7
**16**	15.3 ± 0.4
**17**	15.9 ± 0.4
**TTPC1**	16.4 ± 0.7

a IC_50_, the minimum inhibitory concentration that resulted in 50% cell viability; A549, epithelial lung adenocarcinoma cell line.

b IC_50_ values are presented as means ± standard deviation (SD).

These results agree with previous studies, in which several triterpenes, including ganoderic acid and related compounds isolated from *Ganoderma lucidum*,^25^ were shown to induce apoptosis in A549 tumor cells through downregulation of the antiapoptotic Bcl-2 protein and pro-caspase 9. For these triterpenes, an IC_50_ value of 24.63 μg/mL was reported, which is considerably higher than the values obtained in the present work (7.0–7.5 μg/mL). In another study, the cytotoxic potential of six pentacyclic triterpenes (oleanolic acid, ursolic acid, asiatic acid, betulinic acid, betulin, and lupeol) was evaluated against A549 cells, with IC_50_ values ranging from 15 μM for betulinic acid to 80 μM for lupeol.^26^ These values are comparable to those obtained in the present work, supporting the cytotoxic potential of the triterpenes under investigation. Additionally, triterpenes with a friedelane skeleton, such as friedelin and its derivatives, have been shown to exert cytotoxic effects against A549 cells, with IC_50_ values ranging from 19 to 21.6 μM.^23^ For comparison, doxorubicin, a widely used chemotherapeutic agent, exhibited an IC_50_ of 15.12 μM in the same cell line,^23^ highlighting the notable cytotoxic potential of these triterpenes. Taken together, these findings indicate that the compounds tested in the present study exhibit significant cytotoxicity against lung carcinoma epithelial cells (A549), in agreement with previous reports on the activity of structurally related triterpenes.

Pentacyclic triterpenes are widely recognized for their anticancer properties, acting through different mechanisms involved in cytotoxic and antiproliferative activities. Friedelan-3-one (**6**, friedelin) has shown promising activity in various tumor models, including leukemia, breast, colon, and prostate cancers, being able to induce apoptosis, inhibit cell proliferation, and modulate signaling pathways associated with tumor cell survival, such as PI3K/AKT, MAPK, and NF-κB, in addition to promoting the downregulation of antiapoptotic proteins of the Bcl-2 family and activation of caspases.^6,27–29^ Friedelan-3β-ol (**7**) also exhibits cytotoxic activity in head and neck cancer cells (HN22), where its activity has been associated with interference in the cell cycle by promoting arrest in the S phase, which is responsible for DNA replication. In leukemia cells (THP-1 and K-562), friedelan-3β-ol has been reported to induce apoptosis via the intrinsic mitochondrial pathway, characterized by increased expression of pro-apoptotic proteins (BAK) and reduced Bcl-2 levels.^7,30^ Celastrol, a quinone methide triterpenoid biosynthetically derived from friedelane-type skeleton, displays potent cytotoxic activity in several cancer cell lines, including breast, leukemia, lung, ovarian, gastric, liver, and colon cancers. Its antitumor effects have been associated with multiple mechanisms, such as inhibition of cell proliferation and regulation of the cell cycle, induction of apoptosis and autophagy, inhibition of cell invasion and metastasis, anti-inflammatory effects, and interference with angiogenesis and tumor immunoregulation processes.^31,32^ Although friedelane-type triterpenes exhibit promising cytotoxic activity against different cancer cell lines, the molecular mechanisms involved remain poorly explored and are largely restricted to the most studied compounds, highlighting the need for further investigations to elucidate their mechanisms of action and their potential as prototypes of antitumor agents.

#### Antiviral Activity Evaluation

The chloroform extract (LCE) from *M. quadrangulata* leaves and the obtained triterpenes **1**, **2**, **4–8**, **10–12**, and **14–17** were tested against ZIKV (PE243). The remaining triterpenes were not tested because of their small quantities. The antiviral and cytotoxic effective concentrations in the nontumoral Vero cells, CC_50_ values, and EC_50_ values were determined by employing the MTT assay, resulting in the corresponding SI for the active samples (Table 5).

**Table 5:** Evaluation of Triterpenes and LCE against ZIKV (PE243) in Vero Cells

Compound	CC_50_ ^a^	EC_50_	SI^b^
LCE	>214 mg/mL	<107 mg/mL	>2
**1**	>200 μM	Inactive	
**2**	194.3 ± 3.9 μM	Inactive	
**4**	80.0 ± 6.4 μM	Inactive	
**5**	21.5 ± 2.6 μM	Inactive	
**6**	118.5 ± 6.3 μM	Inactive	
**7**	155.1 ± 6.2 μM	Inactive	
**8**	>100 μM^d^	Inactive	
**10**	56.5 ± 11.3 μM	Inactive	
**11**	59.7 ± 13.3 μM	Inactive	
**12**	150.1 ± 6.5 μM	Inactive	
**14**	>200 μM	Inactive	
**15**	>200 μM	Inactive	
**16**	>200 μM	Inactive	
**17**	94.7 ± 5.4 μM	Inactive	
Ribavirin^c^	>100 μM	4.1 ± 0.3 μM	>24.39

a Values presented as mean ± standard deviation (SD) of one or two independent triplicates (*n* ≥ 3 data points).

b SI values were calculated as CC_50_/EC_50_.

c Ribavirin was used as an inhibition control.^33^

d Tested only up to 100 μM. LCE, chloroform extract from *M. quadrangulata*.

The CC_50_ values for the compounds ranged from 21.5 to >200 μM, with compound **5** having the lowest value (21.5 ± 2.6 μM) and compounds **1** and **14–16** showing the highest values (>200 μM) (Table 5). The EC_50_ against ZIKV (PE243) was also obtained using the MTT assay under the same conditions, adding a viral suspension (MOI 0.1) to the compound dilutions. Only LCE (<107 mg/mL) exhibited antiviral activity, resulting in a SI > 2. Although LCE showed slight antiviral activity against ZIKV, no other of the isolated compounds were active at the concentrations tested (100 μM). In other words, none of the compounds effectively protected over 50% of the cells from viral cytopathic effects. These results suggest that the antiviral activity observed for LCE is likely due to the presence of other bioactive compounds in the extract. It is also possible to propose the existence of synergy among the constituents of the extracts, which would explain the observed activity for the extracts.^34^

#### Antifungal Activity Evaluation

The objective of the study is to explore the potential of *M. quadrangulata* as a potential source for biopesticides. The percentage of growth inhibition (% IG) was assessed using a mycelial assay^33^ against three phytopathogenic fungi: *Botrytis cinerea*, *Fusarium oxysporum*, and *Alternaria alternata*. Commercial antifungal agents, specifically, methylparaben, Fosbel-Plus (comprising 35% Fosetil Al and 35% Mancozeb), and Ortiva PC (containing 250 g/L azoxystrobin, equivalent to 22.8% p/p) were used as positive controls, and ethanol was used as a negative control. Antifungal activity assays were performed with triterpenes **2**, **4**, **5**, **7**, **12**, **16**, and **17**. The remaining compounds were not included in the assays due to their limited availability.

All evaluated compounds demonstrated low or negligible inhibition against the three species at the highest tested concentration (0.1 mg/mL), and compound **7** exhibited the most significant effect, achieving an inhibition percentage of less than 20%. Consequently, under the experimental conditions employed, none of the tested compounds exhibited a sufficient potential against the fungi evaluated. Nevertheless, this study marks the inaugural assessment of the antifungal activity for these specific compounds.

## Conclusions

The phytochemical investigation of *Maytenus quadrangulata* leaves led to the identification of five new derivatives of triterpenoids with a friedelane skeleton, along with 14 known compounds, some of which were structurally modified through acetylation. The 1D and 2D NMR spectral data for compounds **1** to **5** are reported here for the first time, as well as the identification of compound **15** from a plant source. Compounds **6**, **7**, **13**, **15**, **18**, and **19** are documented in *M. quadrangulata* for the first time. Among the tested compounds, compound **5** exhibited significant cytotoxic activity against the K-562 chronic myeloid leukemia cell line, comparable to that of the positive control imatinib. Furthermore, all compounds displayed notable cytotoxicity against the A549 epithelial lung carcinoma cell line, highlighting their potential as antitumor agents. Finally, the compounds did not show notable antiviral activity against the Zika virus or antifungal activity against the phytopathogenic fungi *Alternaria alternata*, *Botrytis cinerea*, and *Fusarium oxysporum*.

## Experimental Section

### General Experimental Procedures

The chromatographic columns (CC) and thin-layer chromatography (TLC) plates were performed using silica gel 60 (70–230 Mesh or 230–400 Mesh) and silica gel 60 G, respectively. 1D and 2D NMR spectra were obtained on Bruker Avance DRX-600, DRX-400 or DRX-200 spectrometers. The deuterated solvents used are indicated in each case. Chemical shifts (δ) were recorded in ppm using tetramethylsilane (TMS) as an internal reference standard, and coupling constants (*J*) were expressed in Hz. Uncorrected melting temperatures were determined using a Microquímica MQAPF-302 apparatus. Infrared spectra were recorded on a Shimadzu IR-408 spectrometer using KBr pellets (1% w/w). Mass spectra of compounds, in both positive and negative modes, were acquired using a Thermo Scientific Q-Exactive Orbitrap mass spectrometer. Compounds were confirmed and detected based on exact mass and isotopic parameters obtained from MS^1^ and MS^2^ spectra. The parameters for the ESI ionization source were as follows: flow 20 μL/min, spray voltage 5200 V, capillary temperature 300 °C, auxiliary gas temperature 37 °C, sheath gas 12, s-lens 50. The parameters used in the APCI ionization source were: flow 20 μL/min, spray voltage 5000 V, capillary temperature 320 °C, auxiliary gas temperature 37 °C, sheath gas 10, s-lens 50.

### Plant Material

The plant material was identified and collected by Professor Dr. Maria Olívia Mercadante Simões from the Department of General Biology at the State University of Montes Claros (Brazil). The leaves of *Maytenus quadrangulata* were collected in the Brejo do Amparo district, municipality of Januária, Minas Gerais, Brazil, in February 2017. These collections were registered with the National System for the Management of Genetic Heritage (SisGen) (registration number A639A9E). A voucher specimen has been deposited in the Herbarium of the State University of Montes Claros (UNIMONTES) (code HMC, number 405).

### Extraction and Isolation

The *M. quadrangulata* leaves (1.74 kg), after air-drying, were pulverized and subjected to maceration extraction at room temperature. The solvents used were hexane, chloroform, ethyl acetate, and methanol, all pure and previously distilled. After filtration, the solvent was removed by using a rotary evaporator under reduced pressure, thus generating the respective extracts. Upon addition of hexane to the chloroform extract (LCE: 44.6 g), a white solid precipitated. This solid was filtered under reduced pressure, resulting in a chloroform solid extract (LCES: 28.3 g) and a filtered chloroform extract (LCEF: 16.0 g), which were processed separately.

The solid LCES (28.3 g) underwent purification by column chromatography (CC), using silica gel 60 (811.0 g, 230–400 Mesh), resulting in 150 fractions (250 mL each) grouped (A1–A27) based on their thin-layer chromatography (TLC) profile. From group A2, the triterpene friedelan-3β-ol (**7**) (hexane-CH_2_Cl_2_ (4:6); 96.6 mg) was obtained. Groups A4, A7, and A8 were subjected to successive CC, leading to the isolation of the following triterpenes: friedelan-3-one (**6**) (A4; hexane-CH_2_Cl_2_ (4:6); 12.6 mg), friedelan-3β-ol (**7**) (A4; hexane-CH_2_Cl_2_ (4:6); 21.9 mg), friedelan-3α-ol (**8**) (A4; hexane-CH_2_Cl_2_ (4:6); 101.7 mg), friedelane-3,7-dione (**9**) (A4; hexane-CH_2_Cl_2_ (4:6); 8.2 mg), 11β-hydroxyfriedelan-3-one (**10**) (A7; hexane-CHCl_3_ (1:9); 46.5 mg), 3β-hydroxyfriedelan-7-one (**11**) (A7; hexane-CHCl_3_ (1:9); 38.9 mg), 3α-hydroxyfriedelan-7-one (**12**) (A8; hexane-CHCl_3_ (1:9) to CHCl_3_; 584.8 mg), friedelane-2α,3α-diol (**13**) (A8; CHCl_3_; 8.9 mg). Upon adding acetone to group A9, a slightly greenish solid precipitated, which was filtered under reduced pressure to obtain 3.0 g of solid (A9s). The solid A9s underwent CC, resulting in 103 fractions (15 mL each), grouped (B1–B4) according to their TLC profile. Groups B1, B2, and B3 were subjected to CC, leading to the isolation of the mixture of triterpenes friedelane-2α,3α-diol (**13**) and friedelane-3α,16β-diol (**18**) (B1; CHCl_3_ to CHCl_3_-ethyl acetate (9:1); 16.0 mg), compound friedelane-3α,11β-diol (**14**) (B2; hexane-CHCl_3_ (9:1); 51.3 mg), and friedelane-3α,25-diol (**15**) (B3; hexane-CHCl_3_ (9:1) to CHCl_3_; 77.8 mg). To group B4 was added acetone, resulting in the precipitation of a solid that was filtered under reduced pressure to obtain the solid (B4s). B4s was partially soluble in chloroform, showing two spots in TLC. The solid B4s underwent consecutive chromatographic columns, resulting in a mixture of unidentified triterpenes (M1) (CHCl_3_-ethyl acetate (95:5) to CHCl_3_-ethyl acetate (9:1); 43.7 mg).

To group A10 (4.1 g) was added ethanol, leading to the precipitation of a solid that was filtered under reduced pressure, generating the solid A10s (2.9 g). The obtained solid, partially soluble in chloroform, exhibited a profile like that of the mixture M1 through the analysis of the ^1^H NMR spectrum. To separate the triterpenes in this mixture, an acetylation reaction was performed with the A10s sample. The triterpene mixture (2.5 g), acetic anhydride (50 mL), and pyridine (60 mL) were added to a flask and kept under agitation at room temperature. The progress of the reaction was monitored by TLC, totaling 30 h of reaction. At the end of the reaction, distilled water was added to the flask cooled in an ice bath, resulting in the precipitation of a white solid. The flask was left at rest in the refrigerator for 3 h to maximize precipitation. Then, the reaction medium was filtered under reduced pressure, and the solid was washed with cold water. After complete drying, 2.6 g of a white solid (M2) was obtained, soluble in chloroform. The solid M2 underwent CC, resulting in 55 fractions (50 mL each), grouped (C1–C6) according to their TLC profile. From groups C1 to C4, the triterpenes friedelane-3α-yl acetate (**16**) (C1; hexane-CHCl_3_ (8:2); 48.8 mg), friedelane-3α,29-diyl diacetate (**1**) (C2; hexane + CHCl_3_ (9:1); 7.3 mg), a mixture of **1** and friedelane-3α,11β-diyl diacetate (**2**) (C3; CHCl_3_; 619.3 mg), and friedelane-3α,11β-diyl diacetate (**2**) (C4; CHCl_3_-ethyl acetate (95:05); 298.8 mg) were obtained. Group C5 underwent CC, resulting in 65 fractions (10 mL each), grouped by the TLC profile (D1-D2). From groups D1 and D2, the mixture of triterpenes friedelane-3β,24-diyl diacetate (**19**) and friedelane-3α,25-diyl diacetate (**3**) (D1; CHCl_3_; 210.3 mg) and the triterpene 11β-hydroxyfriedelane-3α-yl acetate (**5**) (D2; CHCl_3_ + ethyl acetate (9:1); 123.3 mg) were obtained. Group C6 yielded the triterpene 3β-hydroxyfriedelane-24-yl acetate (**4**) (C6; CHCl_3_-ethyl acetate (9:1); 293.5 mg).

### Preparation of the Friedelane-3β,24-diol (**17**)

To obtain the triterpene friedelane-3β,24-diol (**17**), a hydrolysis reaction of compound **4** was performed. The triterpene 3β-hydroxyfriedelane-24-yl acetate (99.7 mg), potassium hydroxide (105.0 mg), and methanol (6 mL) were added to a flask. The flask was refluxed for 1 h, and the reaction progress was monitored by TLC. At the end of the reaction, 30 mL of water were added, leading to the precipitation of a solid that was filtered under reduced pressure and washed with water. After complete drying, 89.1 mg (98% yield) of a white solid, characterized as friedelane-3β,24-diol (**17**), was obtained.

The filtered chloroform extract (LCEF: 16.0 g) appeared as a greenish solid with a plastic-like appearance, suggesting the presence of a significant amount of gutta-percha polymer. In order to separate gutta-percha from the other constituents of the extract, LCEF was subjected to a filtering column using silica gel, with pure methanol and chloroform as eluents, as described by Rodrigues (2015).^35^ Initially, methanol was used as the eluent to remove all constituents of the extract except gutta-percha, as it is insoluble in methanol. Subsequently, the column was eluted with chloroform to remove gutta-percha, which is soluble in this solvent. After solvent removal, 7.0 g of solid were obtained from the fractions eluted with methanol (FECFM), and 7.2 g of solid were obtained using chloroform as the eluent. The latter fraction predominantly consisted of gutta-percha, as confirmed by NMR (Figure S78–79). FECFM underwent consecutive CC, resulting in the isolation of additional quantities of triterpenes **8** (CHCl_3_-ethyl acetate (9:1); 15.8 mg), **10** (CHCl_3_; 32.3 mg), and **14** (CHCl_3_-ethyl acetate (9:1), 4.2 mg).

### Compound **1**: Friedelane-3α,29-diyl Diacetate

White solid with a melting point of 195–196 °C. IR: 2930, 2870, 1738, 1462, 1384, 1246, 1034 cm^–1^. ^1^H and ^13^C NMR data, see Tables 1 and 2. HR-ESI-MS (positive-ion mode): *m*/*z*, calcd. for C_34_H_56_O_4_ [M + Na]^+^: 551.4071; found: 551.4066.

### Compound **2**: Friedelane-3α,11β-diyl diacetate

White solid with a melting point of 241–242 °C. IR 2946, 2868, 1736, 1462, 1382, 1250, 1022, 974 cm^–1^. ^1^H and ^13^C NMR data, see Tables 1 and 2. HR-ESI-MS (positive-ion mode): *m*/*z*, calcd. for C_34_H_56_O_4_ [M + Na]^+^: 551.4071; found: 551.4069.

### Compounds **3** and **19**: Friedelane-3α,25-diyl Diacetate and Friedelane-3β,24-diyl Diacetate

White solid; IR: 2942, 2868, 1738, 1460, 1382, 1244, 1026, 972 cm^–1^. ^1^H and ^13^C NMR data, see Tables 1 and 2. HR-ESI-MS (positive-ion mode): *m*/*z*, calcd for C_34_H_56_O_4_ [M + Na]^+^, 551.4071; found, 551.4069; C_34_H_56_O_4_ [M + K]^+^, 567.3810; found, 567.38062.

### Compound **4**: 3β-Hydroxyfriedelane-24-yl Acetate

White solid with a melting point of 185–187 °C. IR: 3464, 2936, 2868, 1708, 1458, 1384, 1260, 1038 cm^–1^. ^1^H and ^13^C NMR data, see Tables 1 and 2. HR-ESI-MS (positive-ion mode): *m*/*z*, calcd. for C_32_H_54_O_3_ [M + Na]^+^: 509.3965; found: 509.3960.

### Compound **5**: 11β-Hydroxyfriedelane-3α-yl Acetate

White solid with a melting point of 230–232 °C. IR: 3532, 2942, 2868, 1736, 1462, 1384, 1262, 1024, 974 cm^–1^. ^1^H and ^13^C NMR data, see Tables 1 and 2.

### Compound **15**: Friedelan-3α,25-diol

White solid with a melting point of 273–275 °C. IR: 3478, 2934, 2868, 1462, 1384, 1032 cm^–1^. ^1^H and ^13^C NMR data, see Tables 1 and 2. HR-APCI-MS (negative-ion mode): *m*/*z*, calcd. for C_30_H_52_O_2_ [M + Cl]^−^: 479.3661; found: 479.3664; [M - H]^−^: 443.3894; found: 443.3899. HR-APCI-MS (positive-ion mode): *m*/*z*, calcd. for C_30_H_52_O_2_ [M + H - H_2_O]^+^: 427.3934; found: 427.3932.

### Antiviral Activity

#### Cell Lineages and Virus Strain

Zika virus (ZIKV) PE243, which was previously isolated from human with symptomatic infection,^36^ was used for the antiviral assays. Vero (ATCC CCL-81) cells were used for antiviral and cytotoxicity assays. Cells were cultured in Eagle’s minimum essential medium (MEM; Cultilab, Brazil) supplemented with 5% fetal bovine serum (FBS; Cultilab, Brazil), 100 IU/mL penicillin (Cellofarm, Brazil), 100 μg/mL streptomycin (Merck, Germany), and 0.25 μg/mL amphotericin B (Cultilab, Brazil). All cell cultures were incubated at 37 °C and a 5% CO_2_ atmosphere. Viral titration and propagation were performed in Vero cells, as reported.^38^

#### Cytotoxicity and Antiviral Evaluation Using MTT

The 3-(4,5-dimethylthiazol-2-yl)-2,5-diphenyltetrazolium bromide (MTT; Thermo Fischer Scientific, USA) assay was performed to determine the cytotoxic concentration at which the viability of Vero cells was reduced by 50% (cytotoxic concentration of 50% or CC_50_) and the effective concentration at which viral cytopathic effects were reduced by 50% (Effective Concentration of 50% or EC_50_).^37^ Briefly, cells were seeded in 96-well microplates (4.0 × 10^4^ cells per well) and incubated for 24 h in 200 μL of MEM with 1% FBS. After incubation, the medium was removed and 200 μL of MEM with 1% FBS containing a serial dilution of the compounds (200 to 6.25 μM and 214 to 6.68 μg/mL for LCE) was incubated for 72 h. MTT assays were performed as reported.^38^ Linear regression was used to calculate CC_50_ values, considering only those data for which r^2^ > 0.9. Inhibition of cell viability was normalized to serial dilutions of DMSO (from 0.76 up to 1.84% v/v). One or two independent assays in triplicate were performed (*n* ≥ 3 data points). As for the antiviral assays using MTT, the same steps were performed until adding the compounds, which were now diluted in 100 μL of MEM with 1% FBS up to 5-fold from their CC_50_ values. These were added with 100 μL of viral suspensions at a MOI of 0.1, (*i.e*., one viral particle per 10 cells in culture) in MEM (no FBS) and incubated for 72 h, following the same MTT protocol as reported above. The EC_50_ values were calculated relative to the DMSO controls (infected cells in MEM containing serial dilutions of DMSO). These were compared to the hepatitis C drug, ribavirin, as an inhibition control.^33^ Selectivity indexes (SI) were calculated as CC_50_/EC_50_. One independent assay in triplicate was performed *(*n = 3 data points).

### Cytotoxic Activity Assay on Human Leukemia and Breast Adenocarcinoma Cell Lines

Human cell lines used for cytotoxicity analysis were acquired from the American Type Culture Collection (ATCC) and included K-562 (chronic myeloid leukemia, ATCC CCL-243), THP-1 (acute monocytic leukemia, ATCC TIB-202), MDA-MB-231 (human breast adenocarcinoma, ATCC HTB-26), and WI-26 VA4 (human lung embryonic fibroblast, ATCC CCL-95.1). Cells were maintained in RPMI 1640 medium supplemented with 10% fetal bovine serum (FBS), 100 U/mL penicillin, 100 μg/mL streptomycin, and 10 mM HEPES (pH 7.4). Cultures were incubated at 37 °C in a humidified atmosphere with 5% CO_2_ until use in assays.

Cell viability was assessed using the MTT colorimetric assay. Cells were centrifuged at 70*g* for 5 min, the supernatant was discarded, and the pellet was resuspended in complete medium. Cells were then seeded into 96-well plates at a density of 1 × 10^5^ cells per well and incubated at 37 °C in a 5% CO_2_ humidified incubator for 24 h. Test compounds and positive controls (imatinib, cytarabine, and doxorubicin) were prepared in culture medium containing 1% FBS and added to the cells at final concentrations of 100, 10, 1, and 0.1 μg/mL. Following a 48 h incubation period, 100 μL of MTT solution (0.5 mg/mL) was added to each well, and plates were incubated for an additional 3 h. Post-incubation, the supernatant was removed, and 100 μL of DMSO was added to dissolve the formazan crystals. Absorbance was measured at 550 nm using a Versamax microplate reader (Molecular Devices). The minimum inhibitory concentration that resulted in 50% cell viability (IC_50_) was determined by comparing treated cells to untreated controls (considered 100% viable). Dose–response curves were constructed using linear regression analysis. The selectivity index (SI) was calculated as the ratio of the IC_50_ for normal cells (WI-26 VA4) to the IC_50_ for cancer cell lines. All the experiments were conducted in duplicate, and data were analyzed from two independent experiments. Results are presented as mean ± standard deviation (SD).

### Cytotoxic Activity Assay on Lung Carcinoma Epithelial Cell Line

To investigate the cytotoxicity profile of the isolated compounds, human A549 epithelial lung adenocarcinoma cell line (ATCC CCL-185) was utilized. The cells were cultured in 50 mL cell culture flasks using Dulbecco’s Modified Eagle Medium (DMEM) containing 4500 mg/L glucose, 4 mM glutamine, 11 mg/L sodium pyruvate, and 3.7 g/L sodium bicarbonate supplemented with 10% fetal bovine serum and 1% streptomycin. The cultures were maintained at 37 °C in a CO_2_ incubator with 95% O_2_ and 5% CO_2_ to ensure optimal humidity levels.

Once the cells reached the desired confluence (>90%), approximately 1.0 × 10^8^ cells were transferred to 12-well cell culture plates and allowed to adhere for 24 h. The compounds were then prepared in PBS and added to the culture wells. Experimental groups included the following (*n* = 3 per group): (i) sterile NaCl solution (0.9% w/v) as a negative control, (ii) 20% DMSO solution as a positive control, and (iii) compounds **2**, **4**, **5**, **8**, **10**, **12**, **14**, **16** and **17** and the triterpene 3,4-*seco*-friedelan-3,11-olide (**TTPC1**), previously isolated from the hexanic extract of *M. quadrangulata* leaves,^11^ at concentrations of 5, 8, 12, and 15 μg/mL. All cell culture and flow cytometry experiments adhered to the biosecurity standards of ISO 10993–5 (2009). Materials were sterilized prior to use, and cell handling was performed in a BIOSEG 12, Class II type A1 biological safety cabinet. Flow cytometry was conducted using the Fixable Viability Stain 450 (V-450) reagent to distinguish live from dead cells. Each sample was analyzed for 30,000 events to ensure statistical robustness. IC_50_ values and statistical analyses were calculated using GraphPad Prism, version 8.0.

### Antifungal Activity Assay

The fungi *Alternaria alternata*, *Botrytis cinerea*, and *Fusarium oxysporum* were maintained at 25 °C in the dark and periodically subcultured on Petri dishes. The culture medium used was Potato Dextrose Agar with tetracycline to prevent contamination proliferation. Antifungal activity was evaluated as inhibition of mycelial growth using the agar dilution method. Stock solutions of 50 mg/mL were prepared with the compounds using ethanol as a solvent. Colonies grown on Petri dishes were incubated for 48 h for *B. cinerea* and 72 h for *A. alternata* and *F. oxysporum*, and colony growth was digitized and measured. The percentage of inhibition (% *I*) was calculated as % *I* = (*C* – *T*/*C*) × 100, where *C* is the diameter of control colonies and *T* is the diameter of colonies containing the samples. Commercial antifungal agents, specifically, methylparaben, Fosbel-Plus (comprising 35% Fosetil Al and 35% Mancozeb), and Ortiva PC (containing 250 g/L Azoxystrobin, equivalent to 22.8% p/p), were used as positive controls, and ethanol as a negative control.^39^ The samples were tested at concentrations of 0.1 mg/mL, 0.05 mg/mL, and 0.01 mg/mL.

## Supplementary Material



## Notes

IR, HR-MS, and NMR spectra for compounds **1–19** (Figures S1–S81), as well as a table with ^13^C NMR data for compounds **6–14** and **16–18** (Table S1) (PDF)
