# Supplementary material for: Friedelane-Type Triterpenoids from Maytenus quadrangulata as Potential Anticancer Agents
Source: ACS Omega. 2026 May 15;11(21):30663–74. doi: 10.1021/acsomega.5c12356 (PMC13234797; doi:10.1021/acsomega.5c12356)
Supplement: Supplementary file 1 [file ao5c12356_si_001.pdf]

Supplementary Information

Friedelane-type triterpenoids from *Maytenus quadrangulata* as potential anticancer agents

Mariana G. Aguilar,[1] Sandy V. M. Quintão,[1] Túlio R. Freitas,[2] Samuel R. Sabina,[3] Mateus S. M. Serafim,[4] Jônatas S. Abrahão,[4] Adriano P. Sabino,[2] Daniel C. F. Soares,[5] Raimundo M. Cabrera,[3] Ignacio A. Jiménez,[6] Isabel L. Bazzocchi,[6] Grasiely F. Sousa,*[1] Lucienir P. Duarte[1].

[1] Departamento de Química, Universidade Federal de Minas Gerais, 31270-901 Belo Horizonte-MG, Brazil

[2] Departamento de Análises Clínicas e Toxicológicas, Faculdade de Farmácia, Universidade Federal de Minas Gerais, 31270-901 Belo Horizonte-MG, Brazil

[3] Departamento de Botánica, Ecología y Fisiología Vegetal, Facultad de Biología, Universidad de La Laguna, Avenida Astrofísico Francisco Sánchez 2, 38206 La Laguna, Tenerife, Spain

[4] Instituto de Ciências Biológicas, Universidade Federal de Minas Gerais, 31270-901 Belo Horizonte-MG, Brazil

[5] Laboratório de Bioengenharia, Universidade Federal de Itajubá, 35903-087 Itabira-MG, Brazil

[6] Instituto Universitario de Bio-Orgánica Antonio González, Departamento de Química Orgánica, Universidad de La Laguna, Avenida Astrofísico Francisco Sánchez 2, 38206 La Laguna, Tenerife, Spain

* grasielysousa@ufmg.br

Table S1: ^{13}C NMR (100 MHz) data of compounds **6** to **14,** and **16** to **19**.

Posição	6[a]	7[b]	8[b]	9[a]	10[a]	11[a]	12[b]	13[a]	14[a]	16[a]	17[b]	18[a*]	19[a**]
1	22.3	16.3	19.2	21.8	25.1	16.5	19.9	26.7	22.6	19.3	16.7	19.7	16.5
2	41.6	36.3	37.0	41.0	41.7	35.3	36.3	69.8	37.0	32.7	38.9	36.8	32.4
3	213.2	71.6	71.8	211.0	212.8	72.2	71.3	73.4	71.9	75.2	70.7	72.2	74.4
4	58.3	49.8	53.3	58.0	58.2	49.0	53.3	45.8	53.4	50.0	49.6	53.3	48.8
5	42.2	38.2	38.1	47.2	43.2	43.6	44.4	37.9	39.0	38.4	41.5	38.3	40.8
6	41.3	42.1	41.4	57.1	41.4	58.4	57.9	41.1	41.5	41.3	36.1	41.4	36.1
7	18.3	17.8	17.9	210.5	18.1	212.2	212.1	17.8	17.8	17.8	18.9	18.2	17.9
8	53.1	53.4	53.0	63.7	52.9	64.2	63.7	53.0	52.8	52.9	53.6	53.4	53.3
9	37.5	37.3	36.8	42.5	44.2	44.3	42.8	36.5	43.9	37.0	37.1	37.2	37.1
10	59.5	61.8	60.1	59.2	60.2	61.3	59.9	51.4	60.9	59.9	61.7	60.2	61.3
11	35.7	35.8	35.5	35.6	77.0	35.8	36.4	35.4	76.7	35.5	35.8	36.1	36.0
12	30.5	30.8	30.6	30.0	42.2	30.3	30.1	30.5	42.2	30.6	30.8	32.1	30.8
13	39.7	38.5	38.3	39.5	41.2	39.6	39.5	39.7	38.4	38.3	38.5	40.1	38.5
14	38.3	39.8	39.7	37.6	38.4	37.8	37.6	38.3	41.2	39.7	39.7	39.3	39.8
15	32.5	32.5	32.4	32.0	32.6	32.1	31.9	32.4	32.5	32.4	32.2	44.3	32.2
16	36.1	36.1	36.1	36.5	36.0	36.5	35.7	36.1	36.1	36.0	35.9	75.6	35.9
17	30.0	30.1	30.0	30.3	30.1	30.4	30.3	30.0	30.2	30.0	30.0	38.0	30.0
18	42.8	43.0	42.8	42.0	42.7	42.1	42.0	42.8	42.7	42.8	42.9	44.8	42.9
19	35.4	35.5	35.5	35.1	35.5	35.0	35.1	35.4	35.5	35.3	35.3	35.7	35.5
20	28.2	28.3	28.2	28.2	28.3	28.3	28.2	28.2	28.3	28.2	28.2	28.1	28.2
21	32.8	33.0	32.8	33.0	32.8	33.1	33.0	32.8	32.9	32.8	32.8	35.8	32.9
22	39.3	39.4	39.3	38.8	39.3	39.0	38.8	39.3	39.3	39.3	39.3	30.9	39.4
23	6.8	12.2	10.0	7.0	7.0	11.8	10.2	9.8	10.0	9.9	12.0	10.1	14.0
24	14.7	16.7	14.6	15.3	15.0	17.1	14.9	13.6	14.9	14.5	63.4	14.7	65.3
25	18.0	18.5	18.1	18.4	13.0	18.6	19.5	17.9	13.3	18.1	17.6	18.4	18.5
26	20.3	20.3	20.2	19.4	20.2	19.7	19.6	18.7	20.1	20.2	18.7	21.4	18.8
27	18.7	18.8	18.6	19.6	19.7	19.8	18.4	20.2	19.6	18.6	20.0	20.3	20.3
28	32.1	32.2	32.1	32.3	32.2	32.3	31.7	32.1	32.1	32.1	32.1	25.0	32.4
29	35.0	35.1	35.0	31.7	31.8	31.9	34.7	31.8	35.1	35.0	35.0	35.6	35.2
30	31.8	32.0	31.8	34.7	35.2	34.9	32.3	35.1	31.8	31.8	31.8	30.8	31.9
1’	-	-	-	-	-	-	-	-	-	171.2	-	-	171.0
2’	-	-	-	-	-	-	-	-	-	21.4	-	-	21.3
3’	-	-	-	-	-	-	-	-	-	-	-	-	171.2
4’	-	-	-	-	-	-	-	-	-	-	-	-	21.5

[a] $CDCl_3$ [b] $CDCl_3$ + PyD_5 *^{13}C NMR data of **18** in mixture of **13** and **18**. ** Data corresponding to the mixture of compounds **3** and **19**.

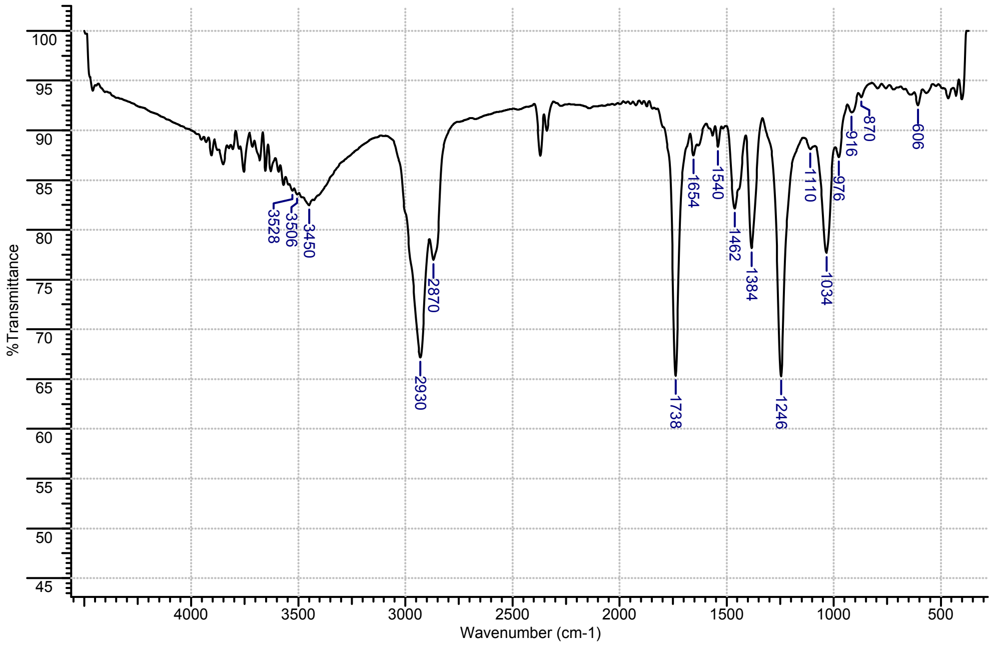

Figure S1: IR spectrum (KBr) of compound **1**.

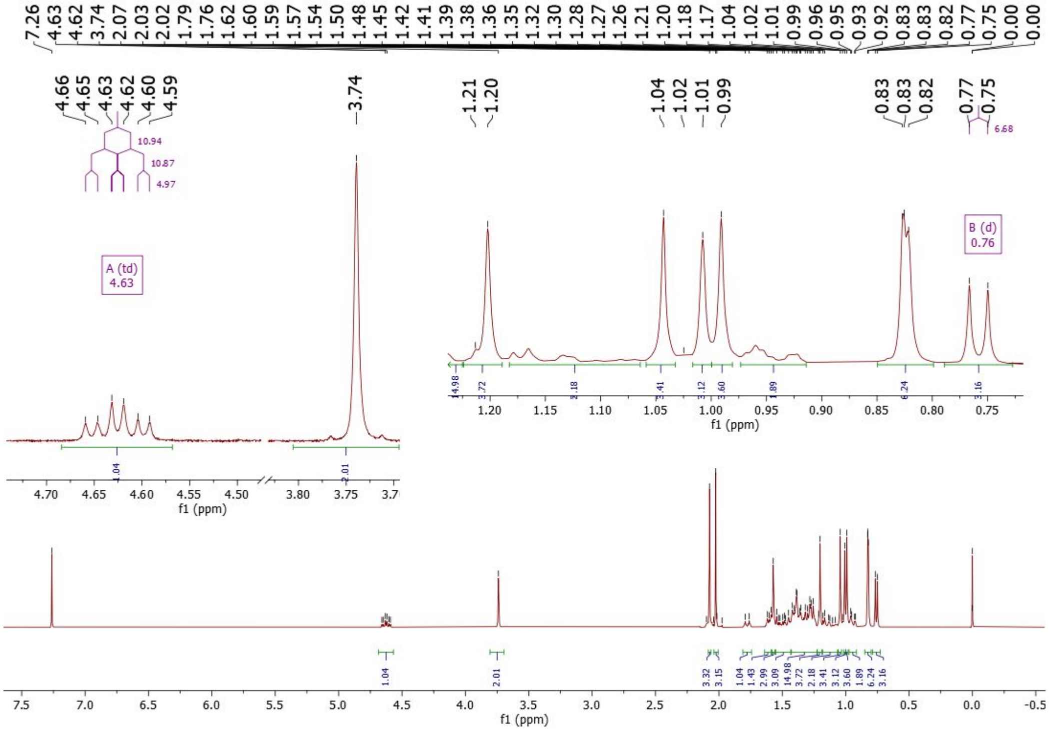

Figure S2: ^{1}H NMR spectrum (400 MHz. $CDCl_3$) of compound **1.**

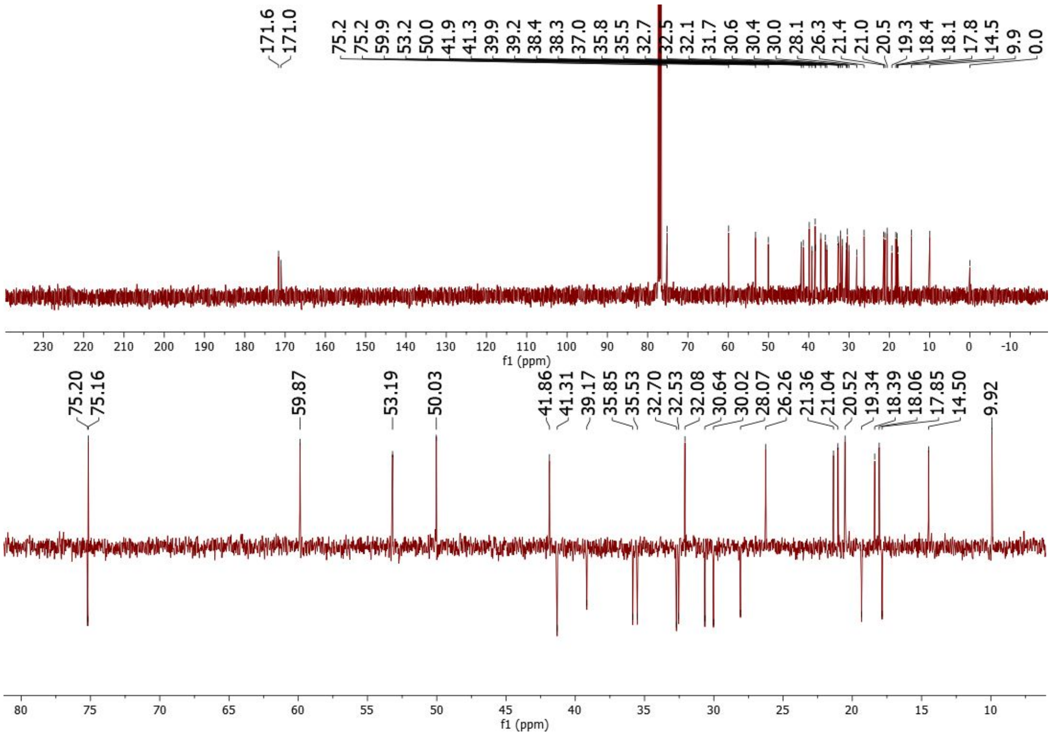

Figure S3: ^{13}C NMR and DEPT-135 spectra (100 MHz. $CDCl_3$) of compound **1.**

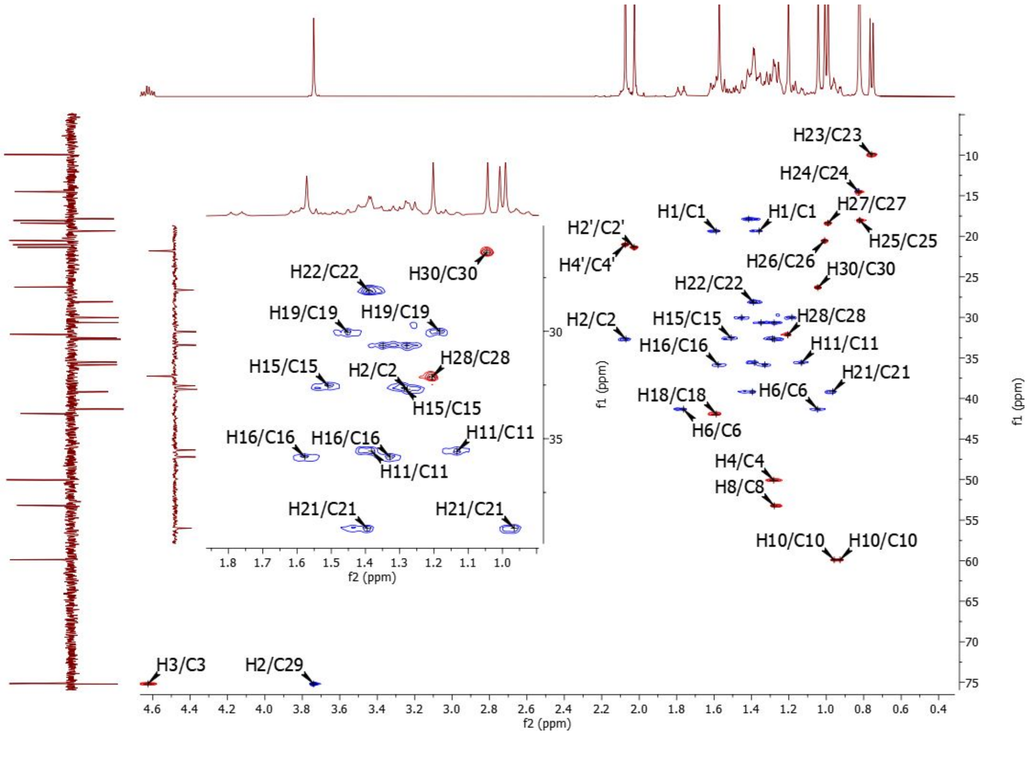

Figure S4: HSQC and expanded HSQC (400 MHz. $CDCl_3$) spectrum of **1** in the region between δ_H 1.8 to 0.9 ppm.

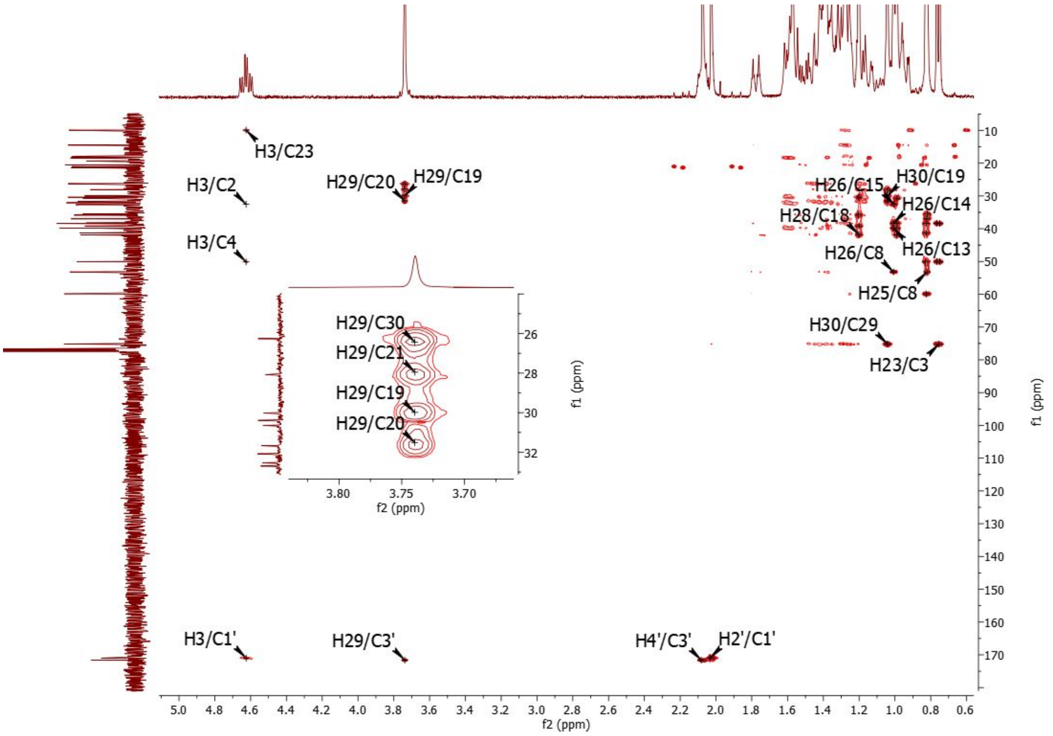

Figure S5: Expanded HMBC (400 MHz. $CDCl_3$) spectrum of **1** in the region between δ_H 3.9 to 3.6 ppm.

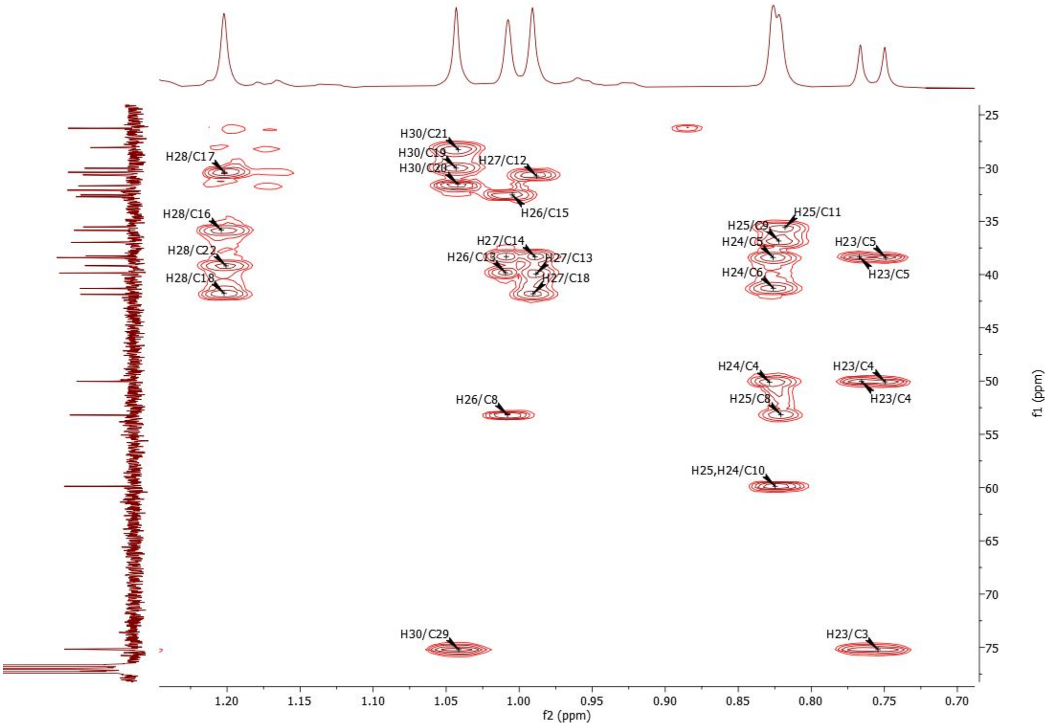

Figure S6: Expanded HMBC (400 MHz. $CDCl_3$) spectrum of **1** in the region between δ_H 1.3 to 0.7 ppm.

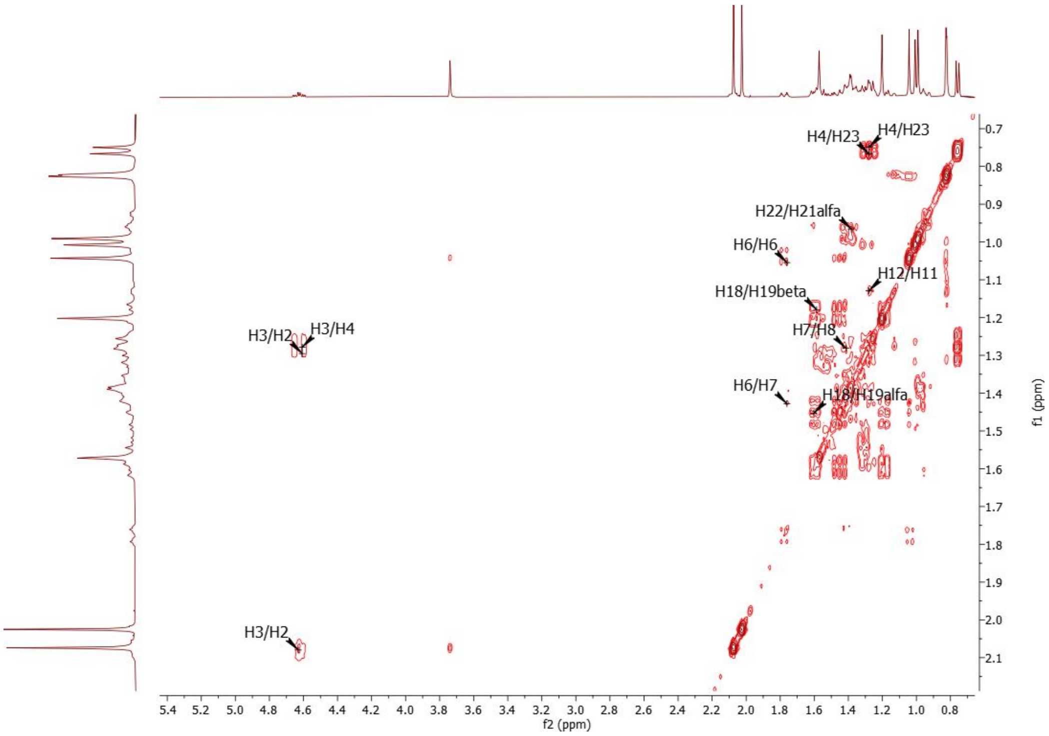

Figure S7: COSY (400 MHz. $CDCl_3$) spectrum of **1**.

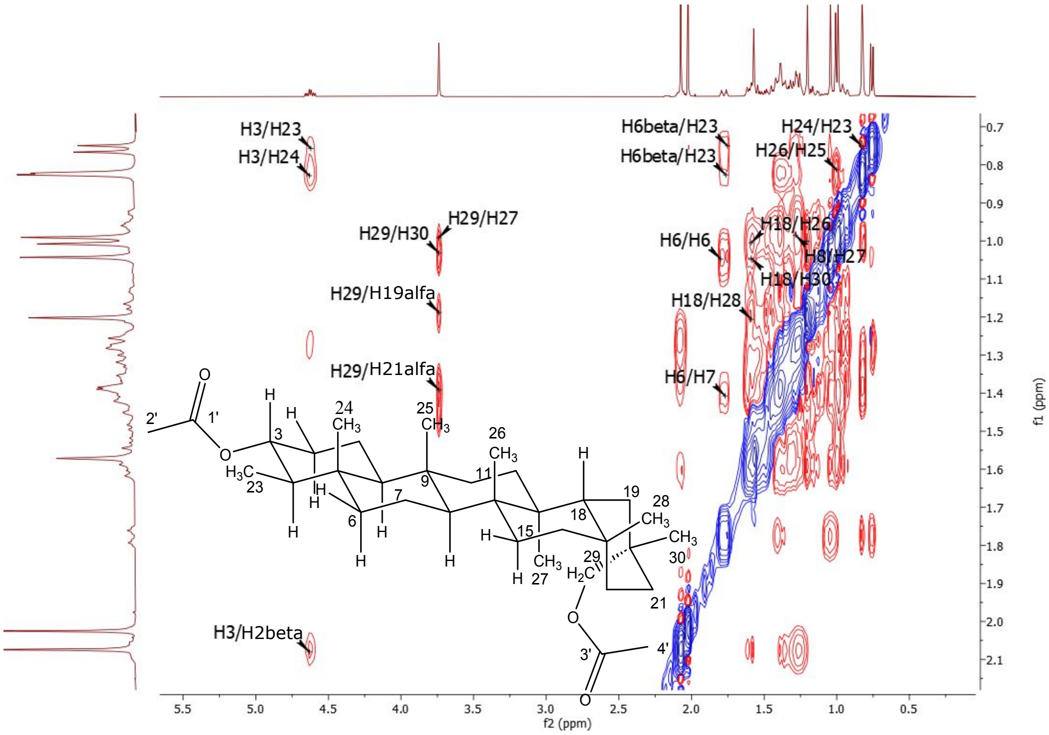

Figure S8: NOESY (400 MHz. $CDCl_3$) spectrum of **1**.

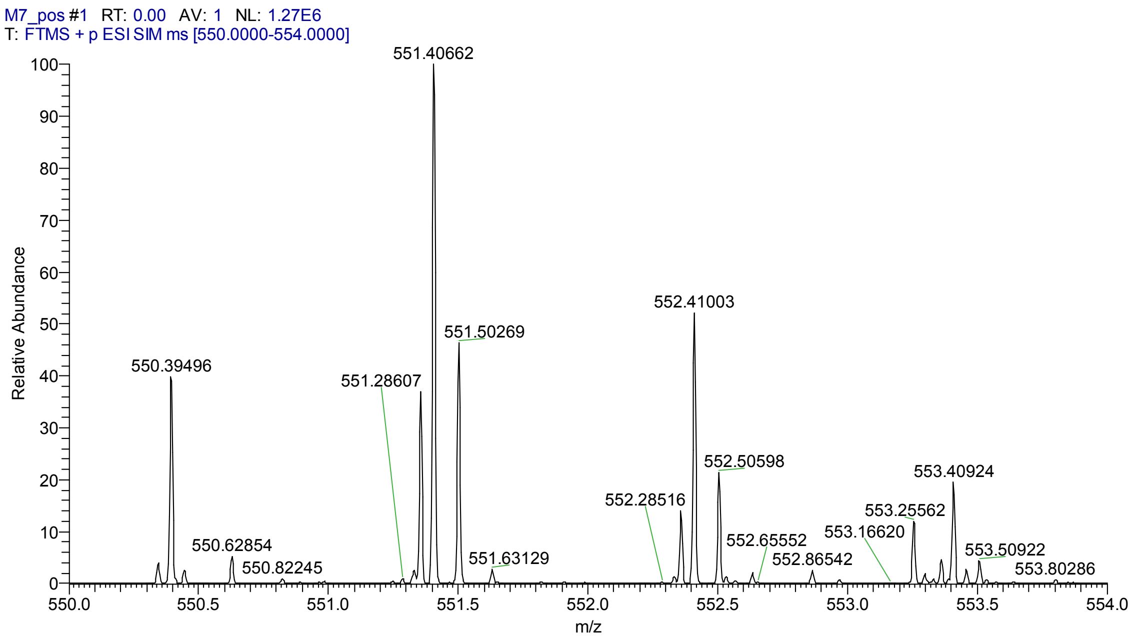

Figure S9: Mass spectrum (HR-ESI-MS) of compound **1**.

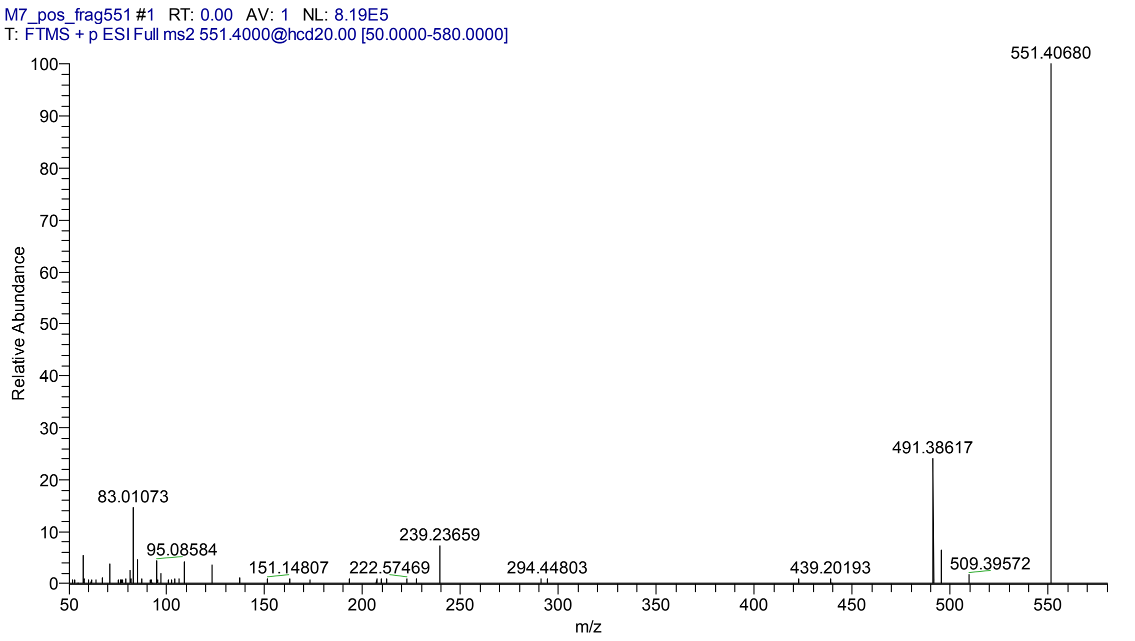

Figure S10: MS^2 spectrum of the 551 *m/z* ion fragmentation (HR-ESI-MS) of compound **1**.

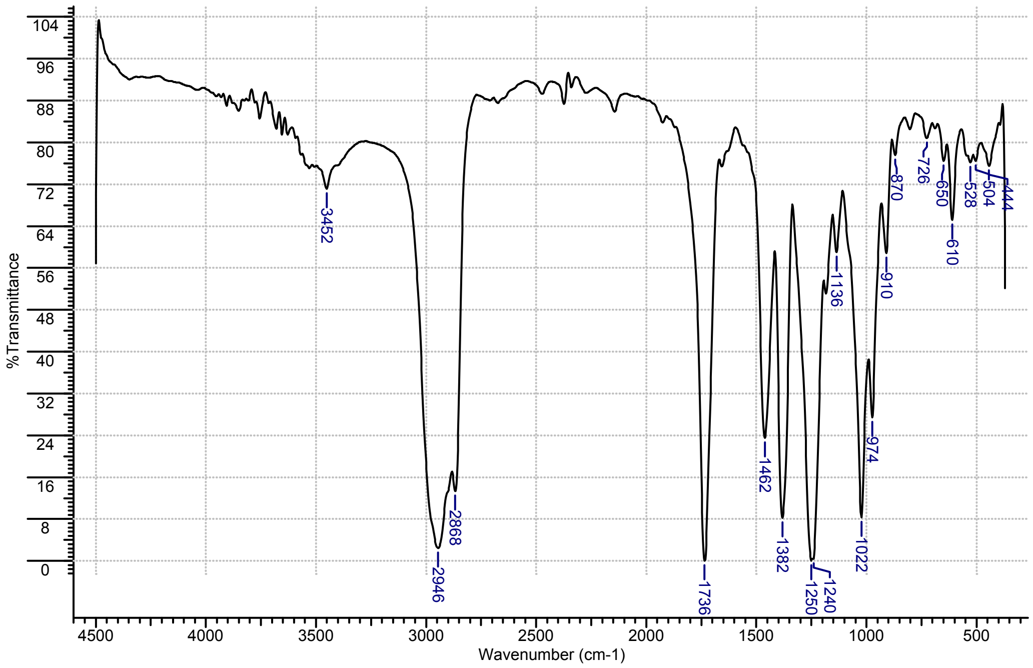

Figure S11: IR spectrum (KBr) of compound **2**.

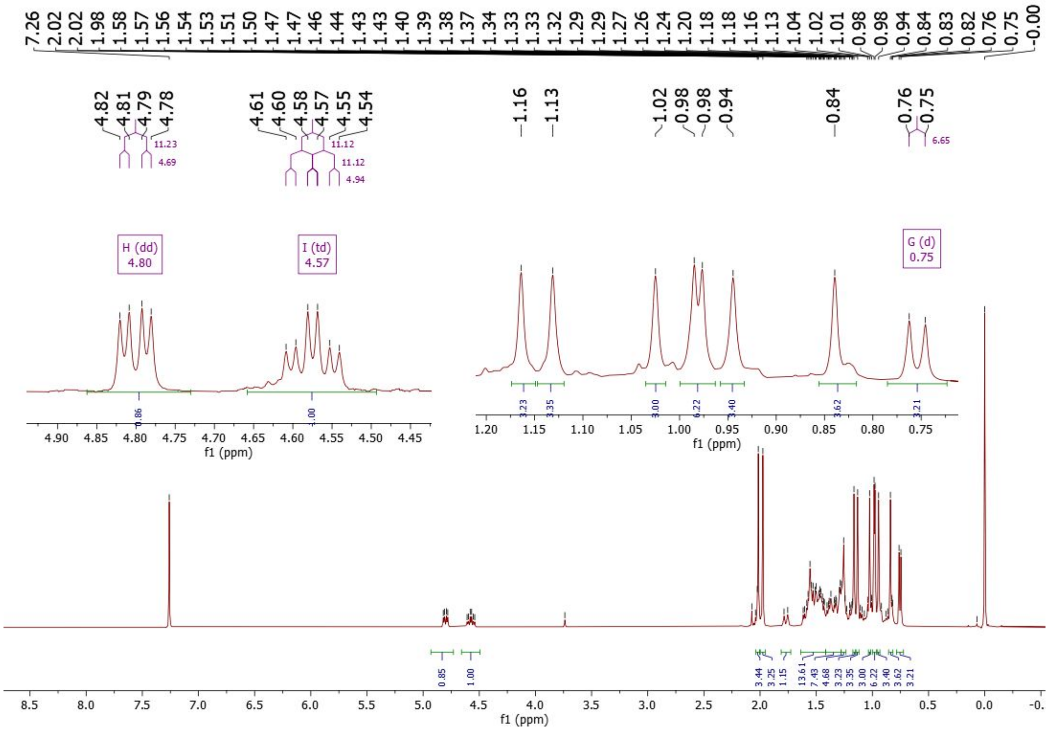

Figure S12: ^{1}H NMR spectrum (400 MHz. $CDCl_3$) of compound **2.**

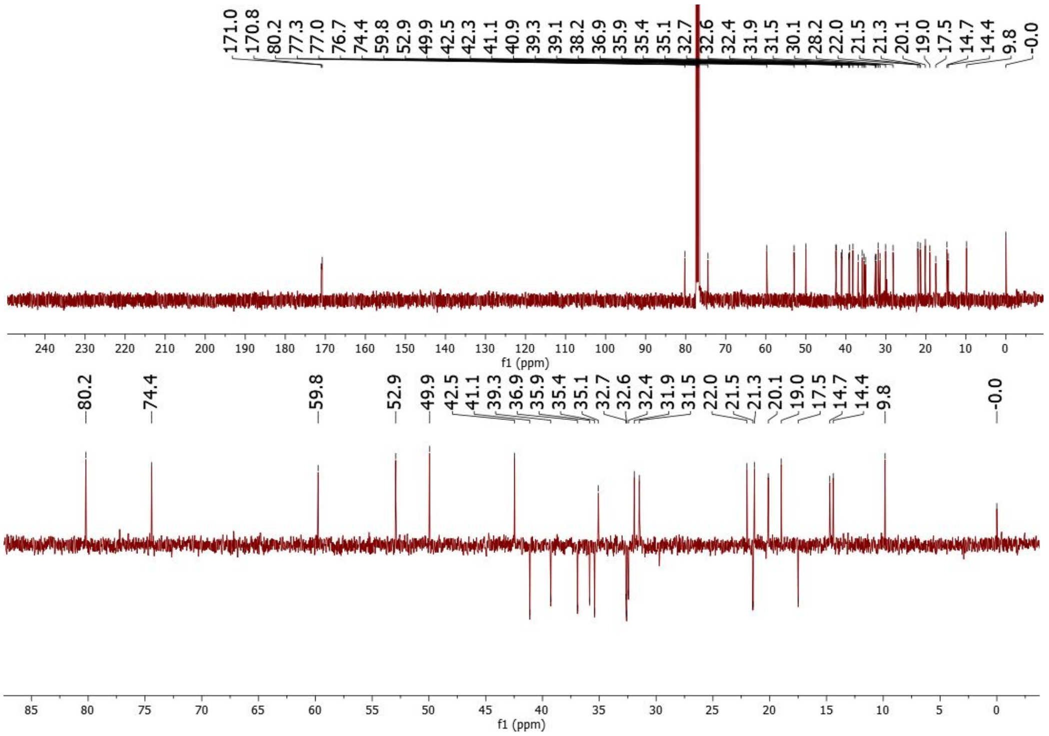

Figure S13: ^{13}C NMR and DEPT-135 spectra (100 MHz. $CDCl_3$) of compound **2**.

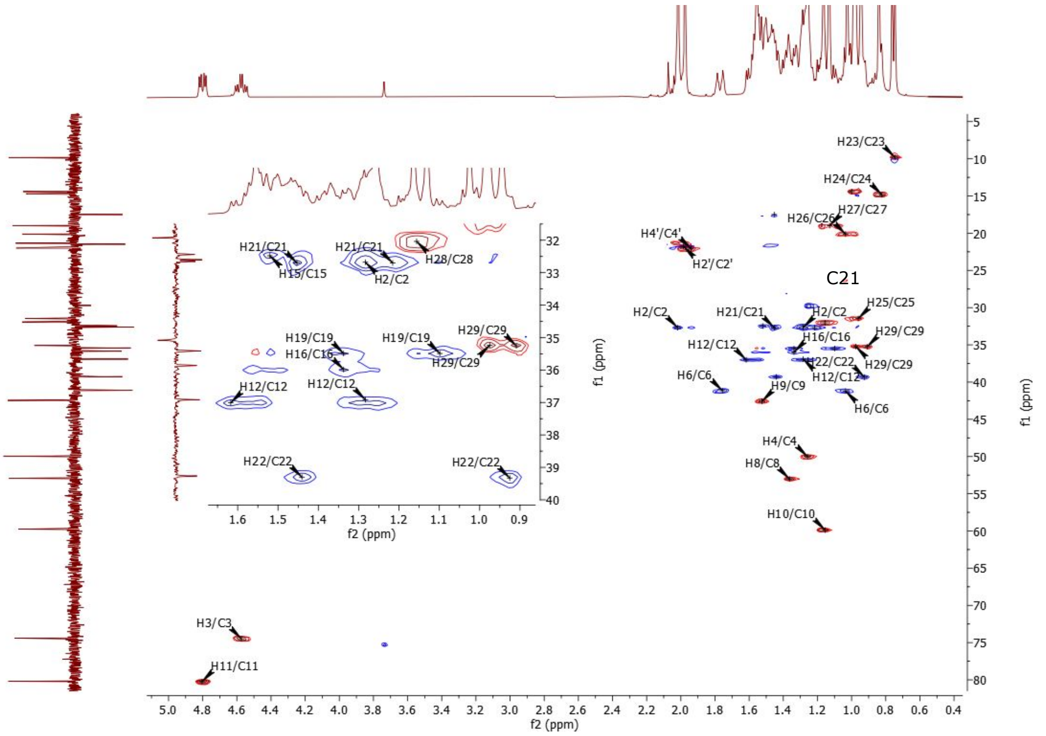

Figure S14: HSQC and expanded HSQC (400 MHz. $CDCl_3$) spectrum of **2** in the region between δ_H 1.6 to 0.9 ppm.

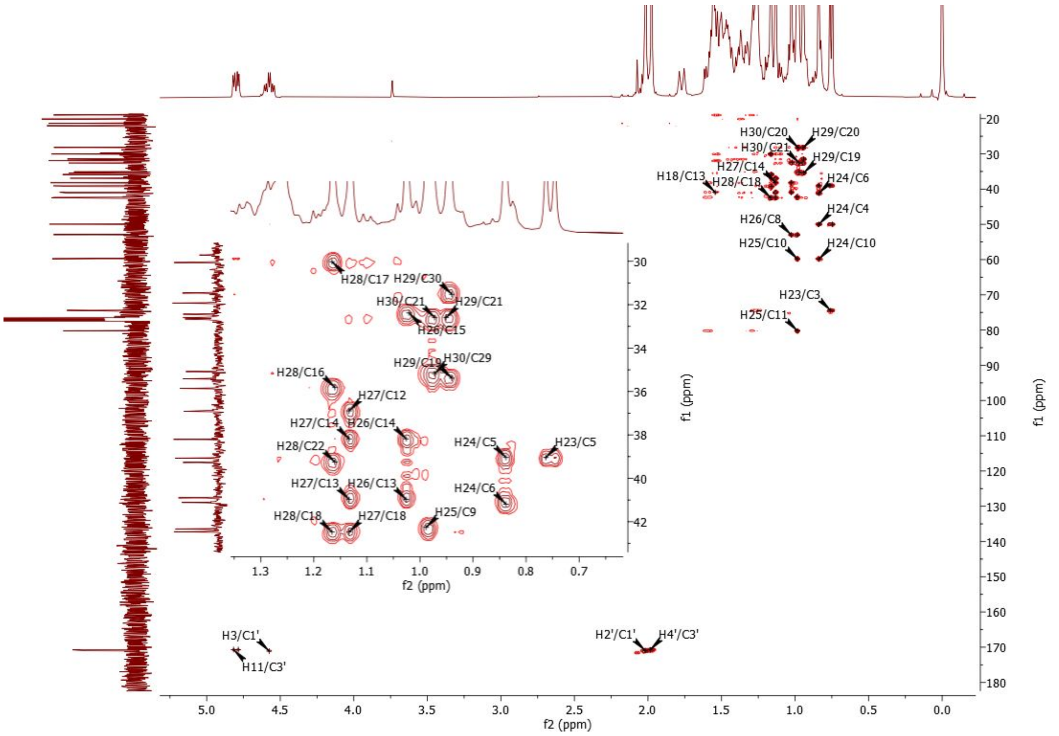

Figure S15: HMBC and expanded HMBC (400 MHz. $CDCl_3$) spectrum of **2** in the region between δ_H 1.3 to 0.6 ppm.

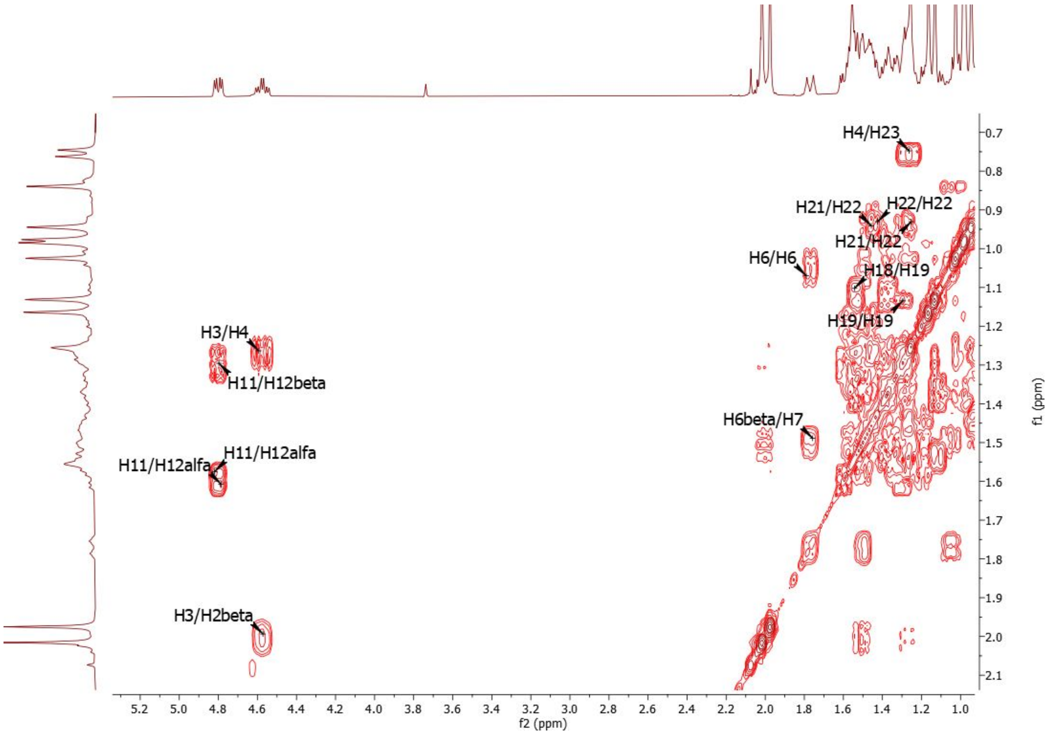

Figure S16: COSY (400 MHz. $CDCl_3$) spectrum of **2**.

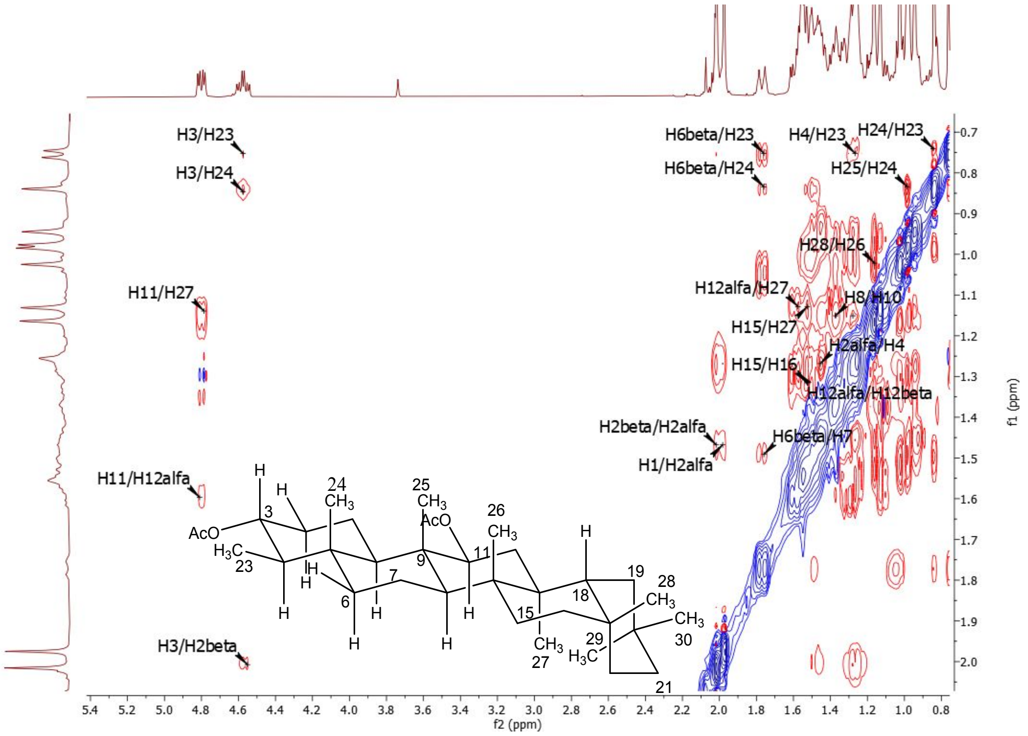

Figure S17: NOESY (400 MHz. $CDCl_3$) spectrum of **2**.

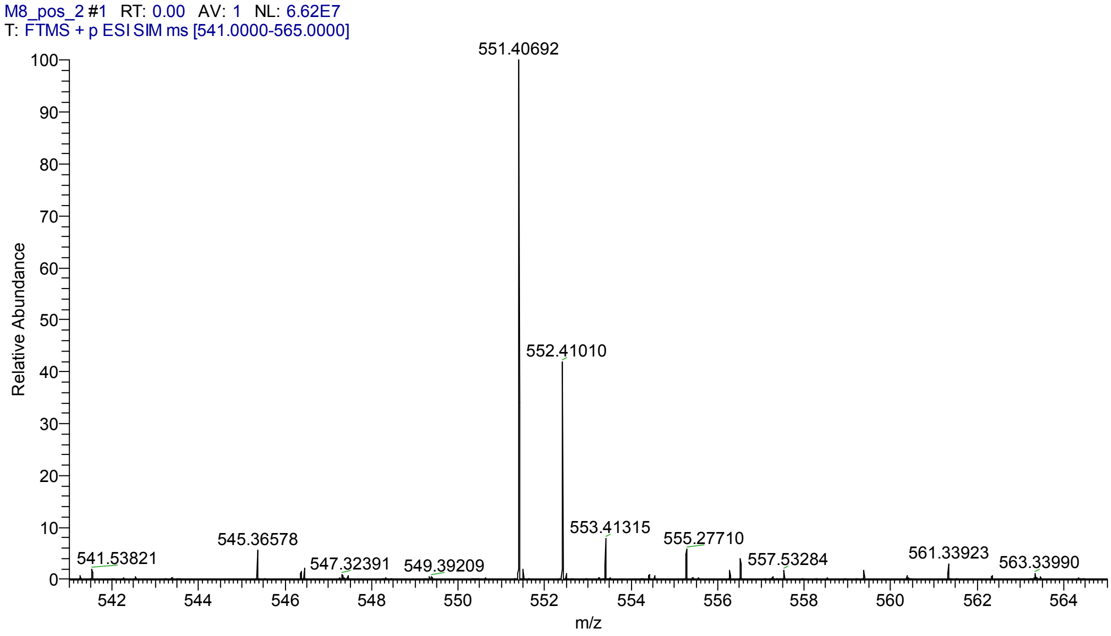

Figure S18: Mass spectrum (HR-ESI-MS) of compound **2**.

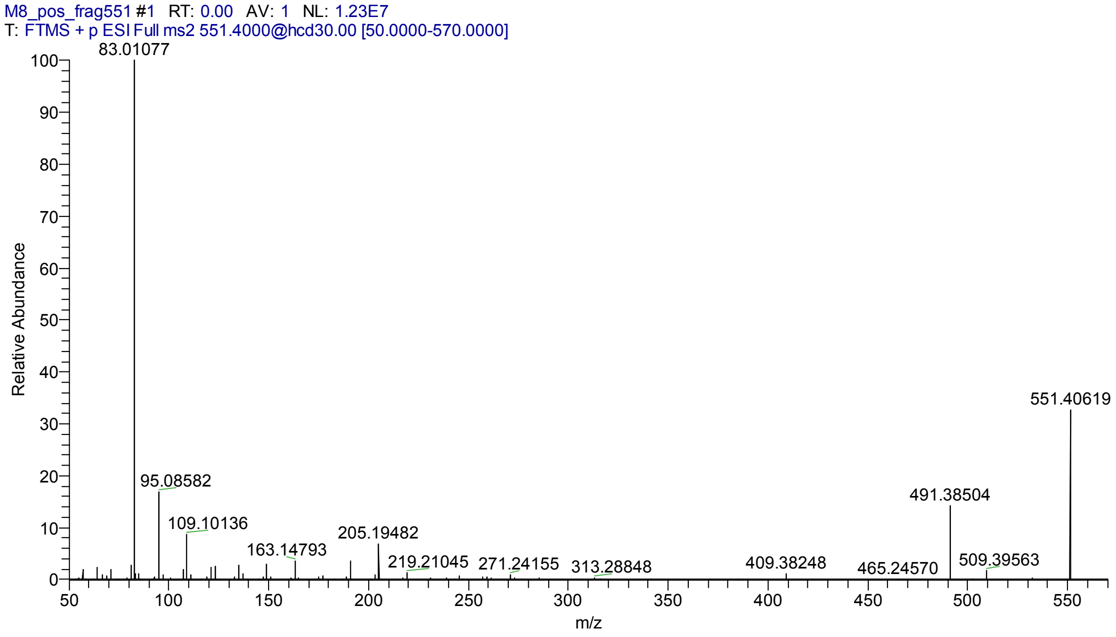

Figure S19: MS^2 spectrum of the 551 *m/z* ion fragmentation (HR-ESI-MS) of compound **2**.

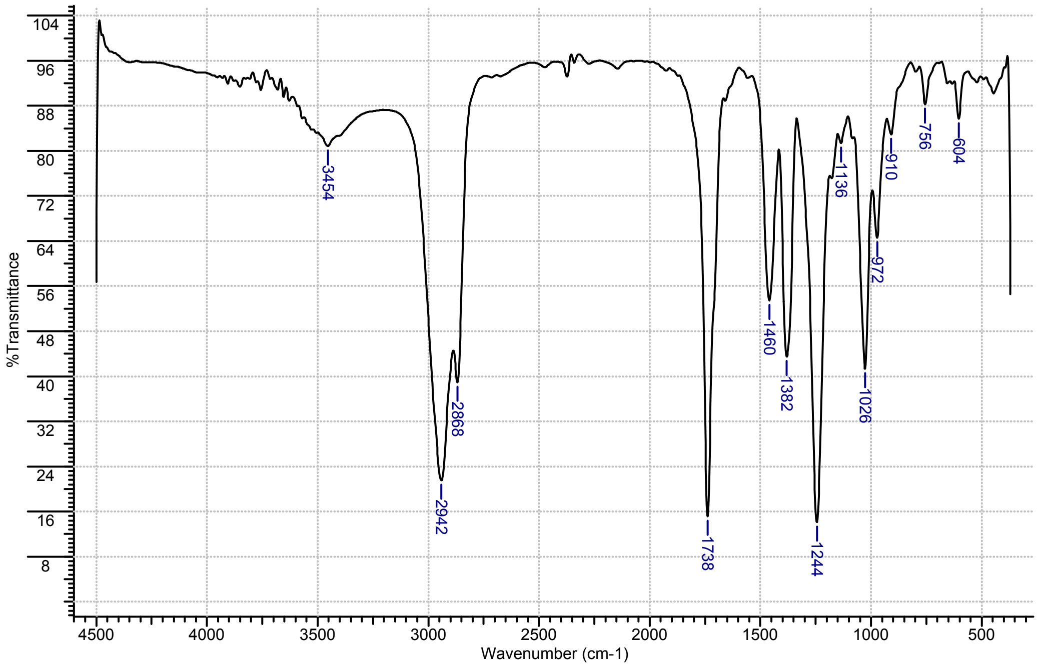

Figure S20: IR spectrum (KBr) of the mixture of compounds **3** and **19.**

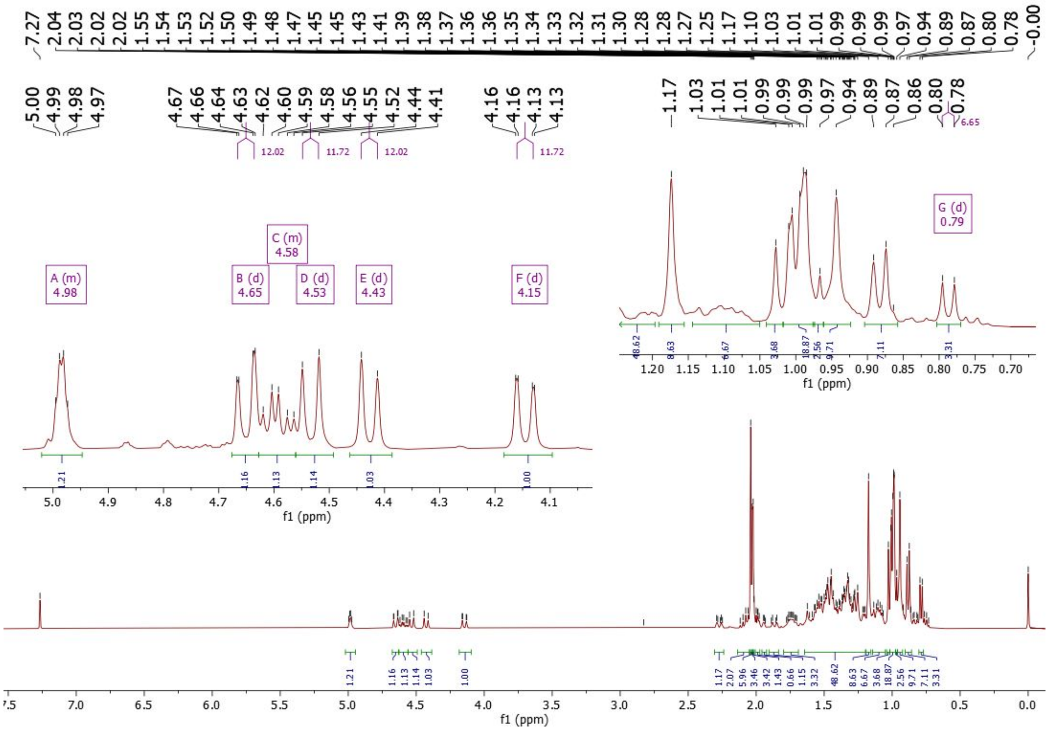

Figure S21: ^{1}H NMR spectrum (400 MHz. $CDCl_3$) of the mixture of compounds **3** and **19**.

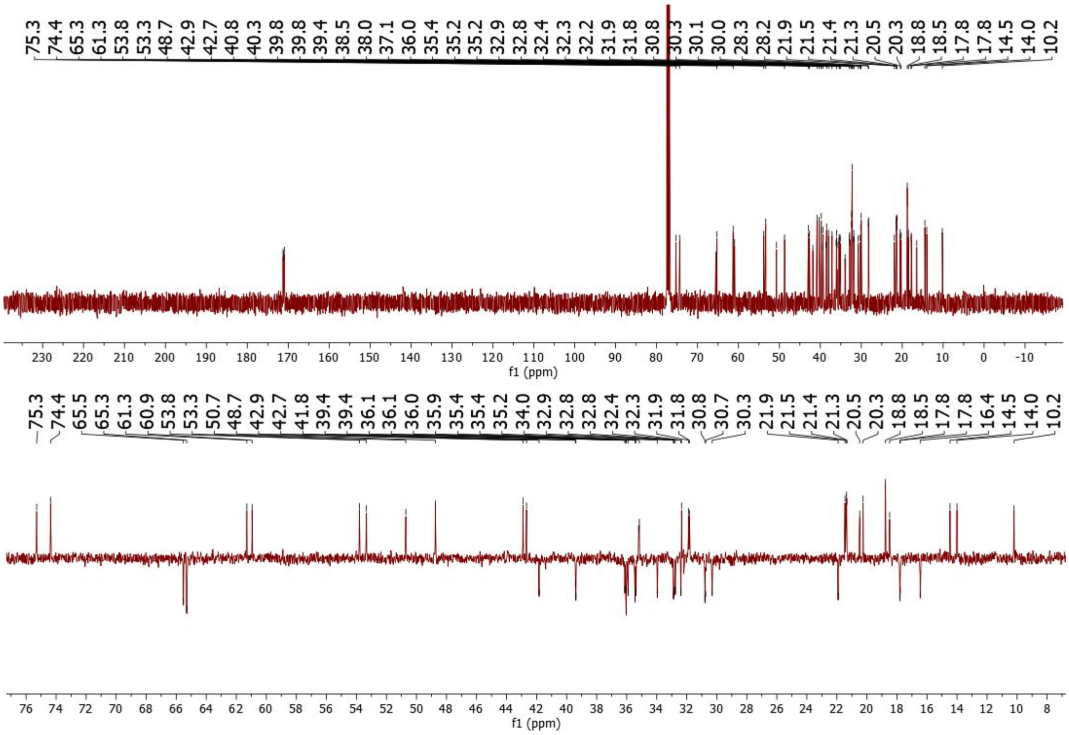

Figure S22: ^{13}C NMR and DEPT-135 spectra (100 MHz. $CDCl_3$) of the mixture of compounds **3** and **19.**

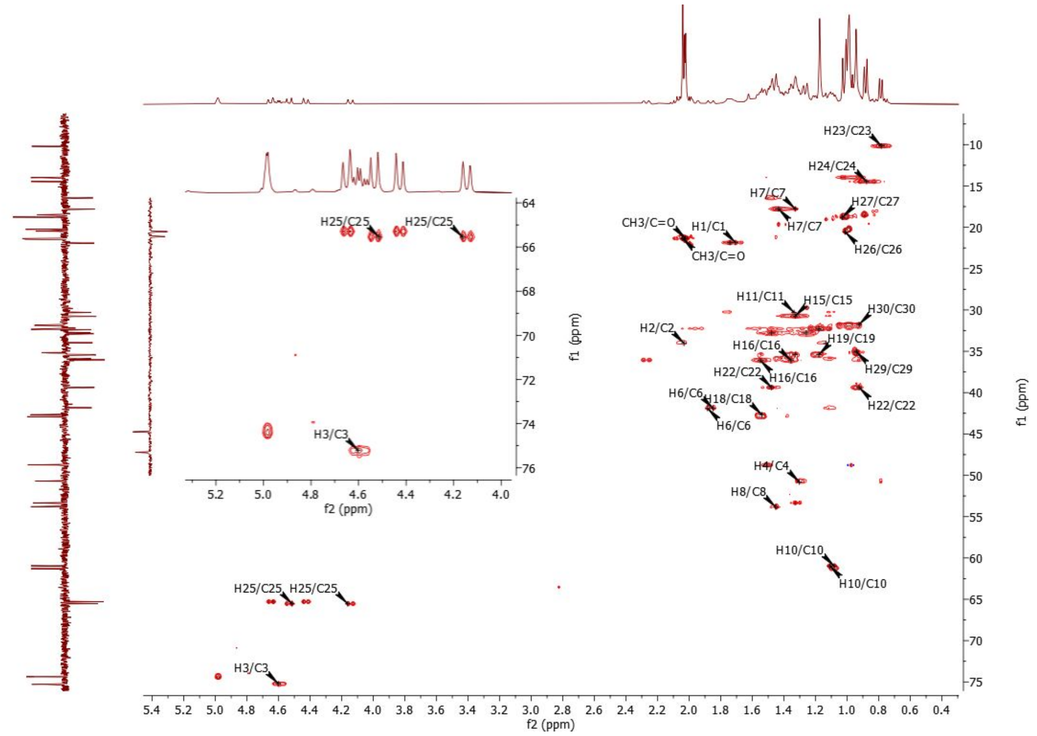

Figure S23: HSQC and expanded HSQC (400 MHz. $CDCl_3$) spectrum of the mixture of compounds **3** and **19** in the region between δ_H 5.2 to 4.0 ppm.

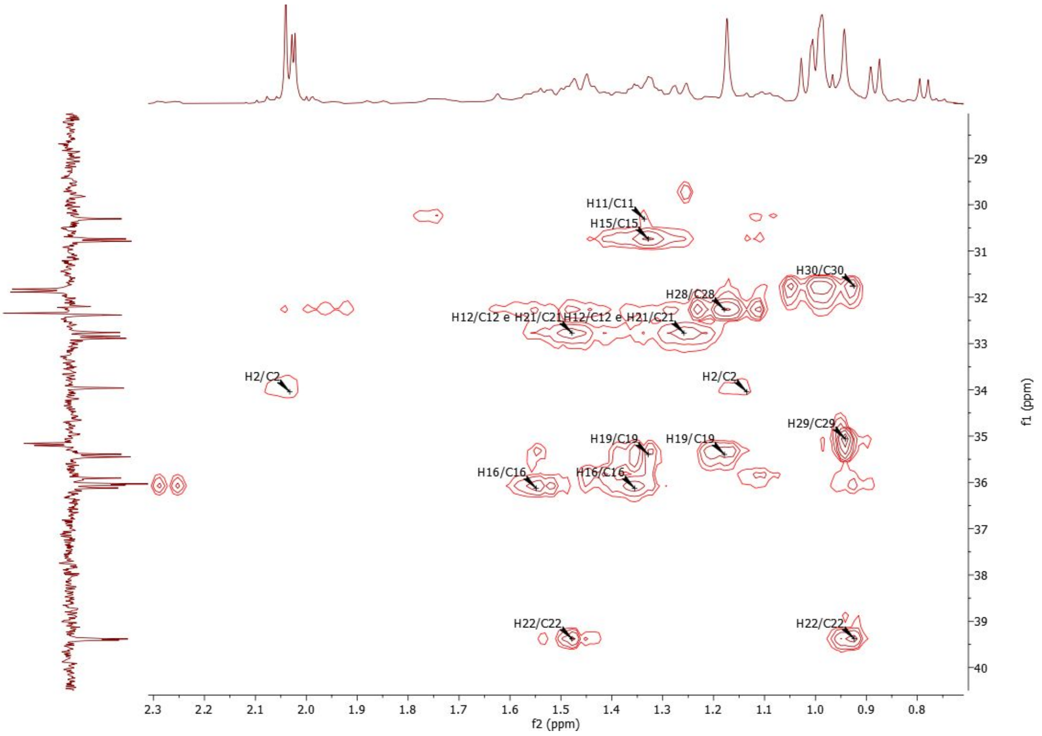

Figure S24: Expanded HSQC (400 MHz. $CDCl_3$) spectrum of the mixture of compounds **3** and **19** in the region between δ_H 2.3 to 0.7 ppm.

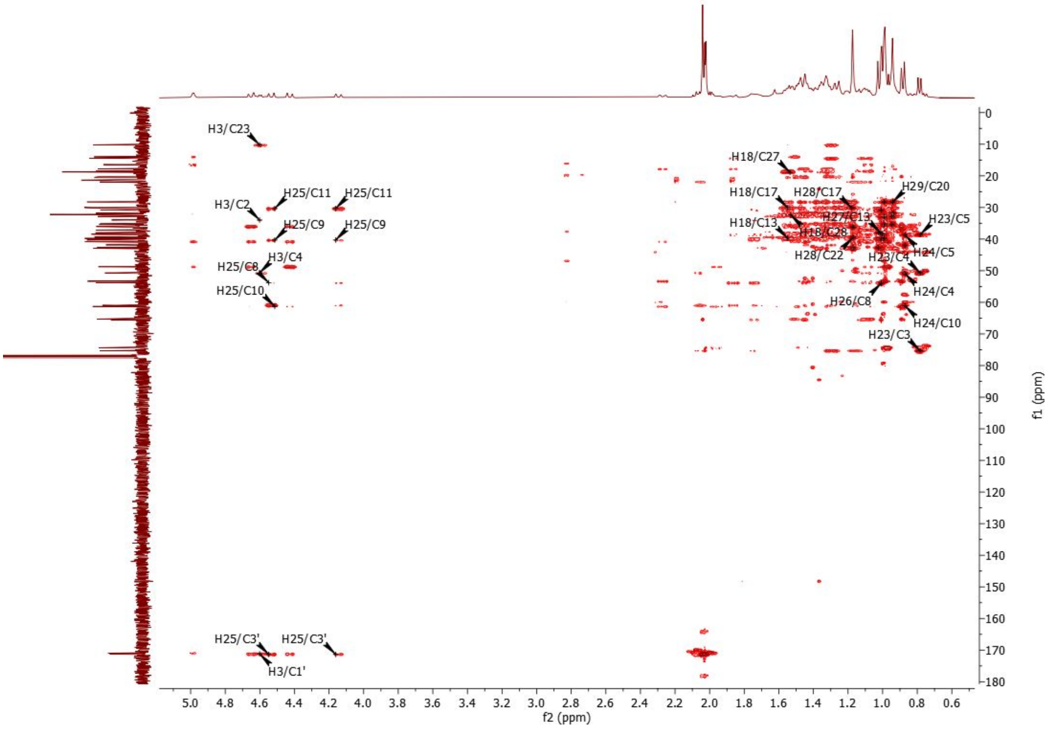

Figure S25: HMBC (400 MHz. $CDCl_3$) spectrum of the mixture of compounds **3** and **19**.

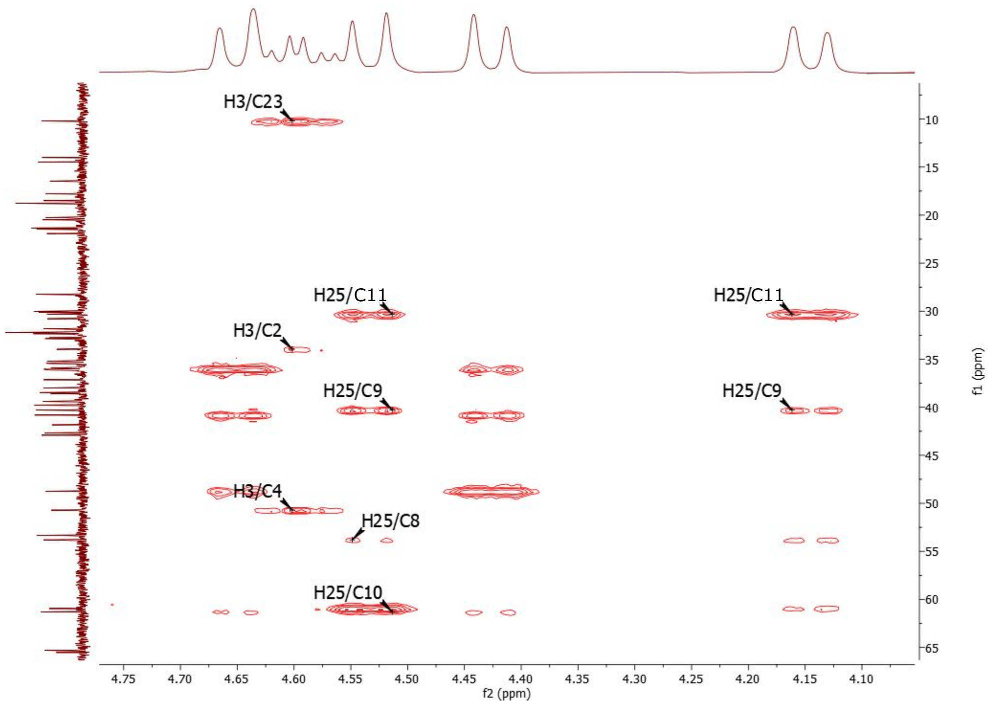

Figure S26: Expanded HMBC (400 MHz. $CDCl_3$) spectrum of the mixture of compounds **3** and **19** in the region between δ_H 4.8 to 4.0 ppm.

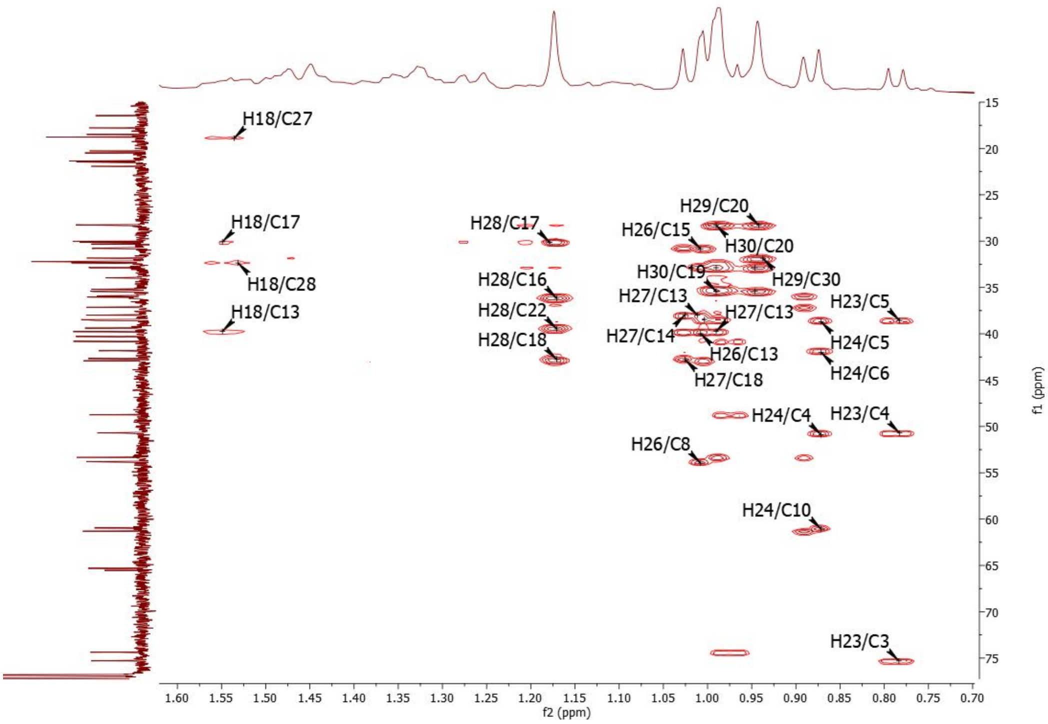

Figure S27: Expanded HMBC (400 MHz. $CDCl_3$) spectrum of the mixture of compounds **3** and **19** in the region between δ_H 1.4 to 0.7 ppm.

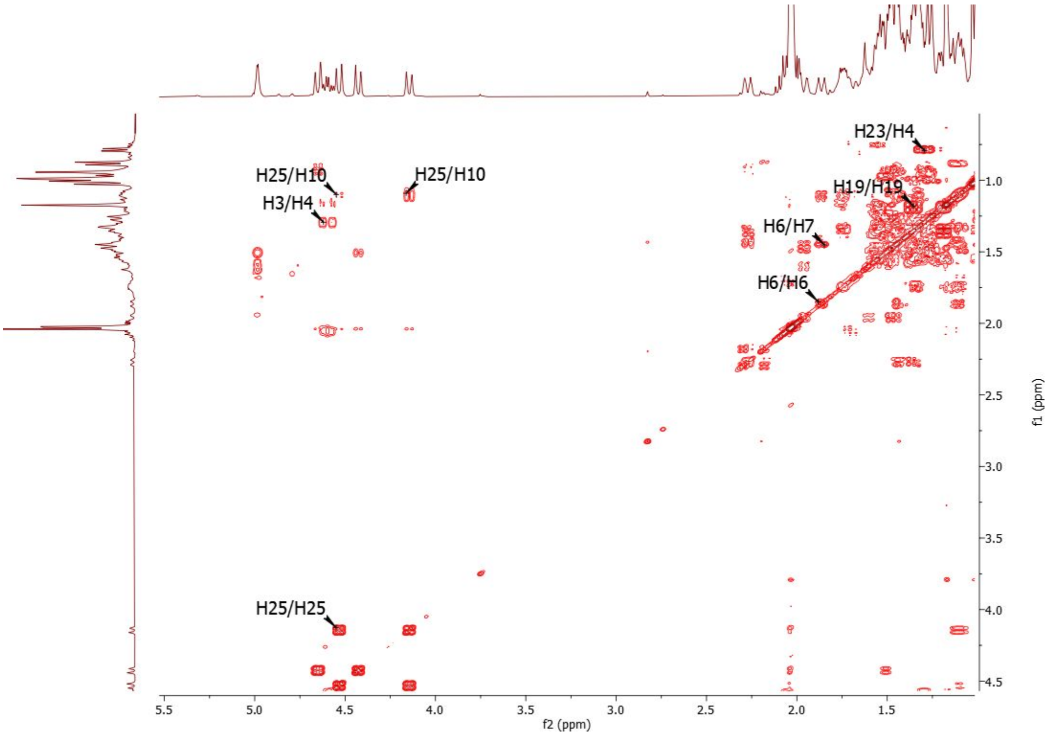

Figure S28: COSY (400 MHz. $CDCl_3$) spectrum of the mixture of compounds **3** and **19**.

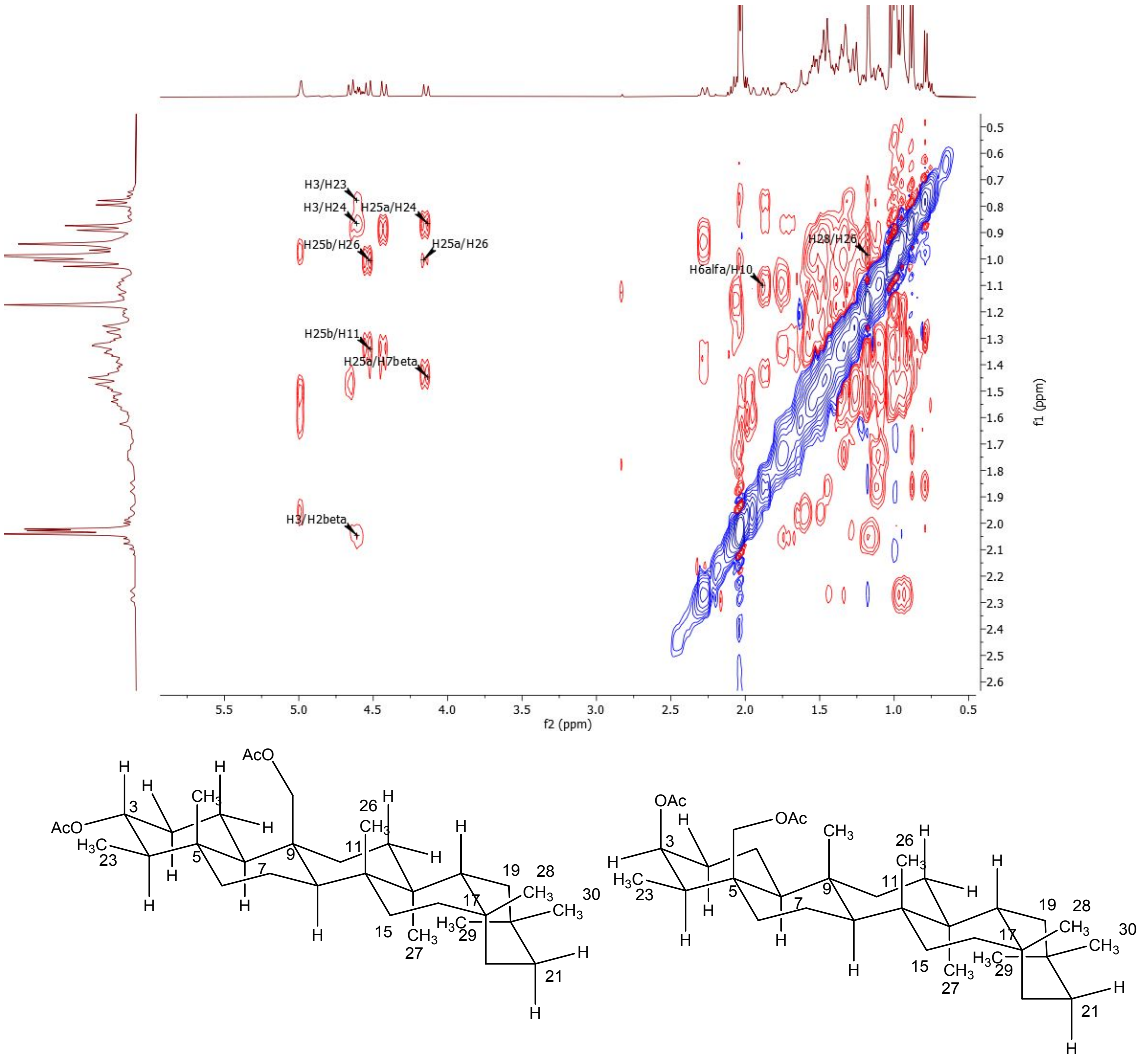

Figure S29: NOESY (400 MHz. $CDCl_3$) spectrum of the mixture of compounds **3** and **19**.

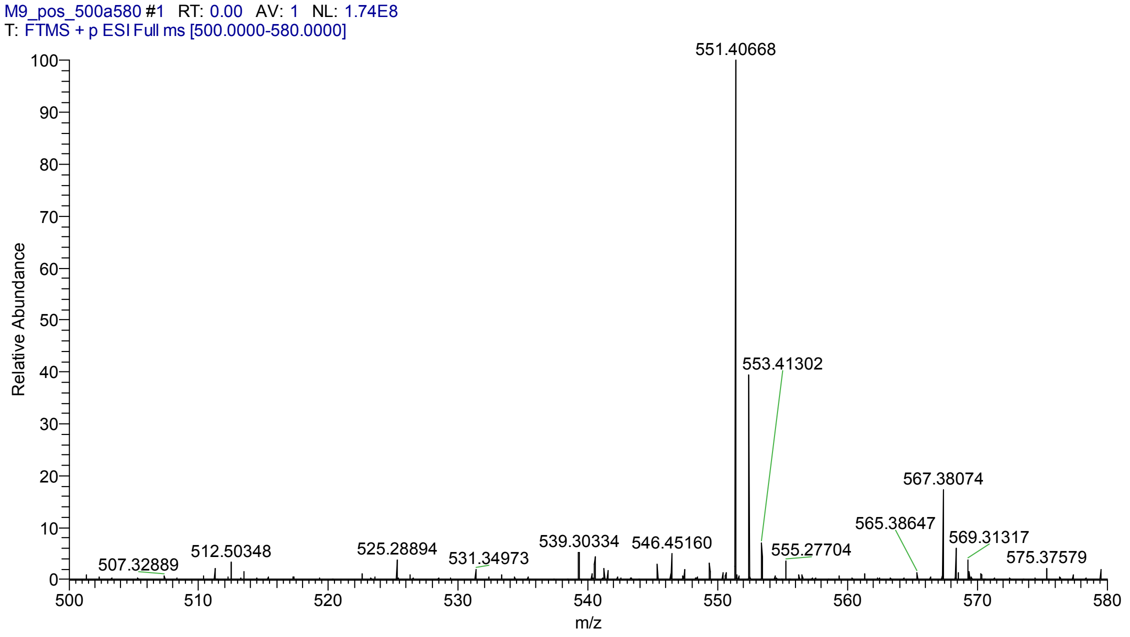

Figure S30: Mass spectrum (HR-ESI-MS) of the mixture of compounds **3** and **19**.

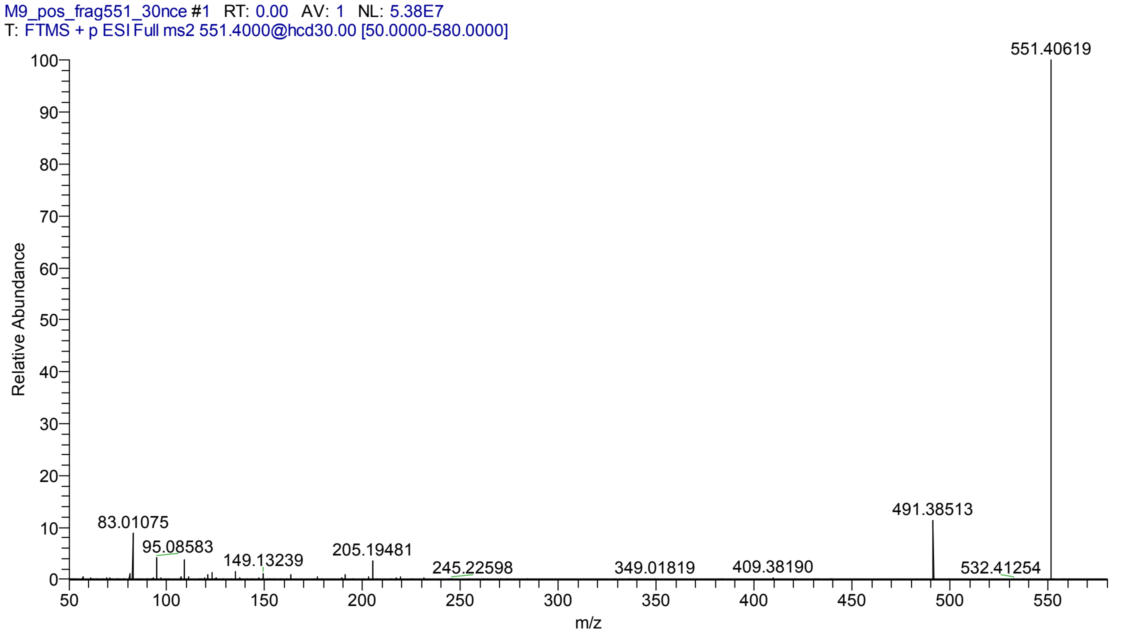

Figure S31: MS^2 spectrum of the 551 *m/z* ion fragmentation (HR-ESI-MS) of the mixture of compounds **3** and **19.**

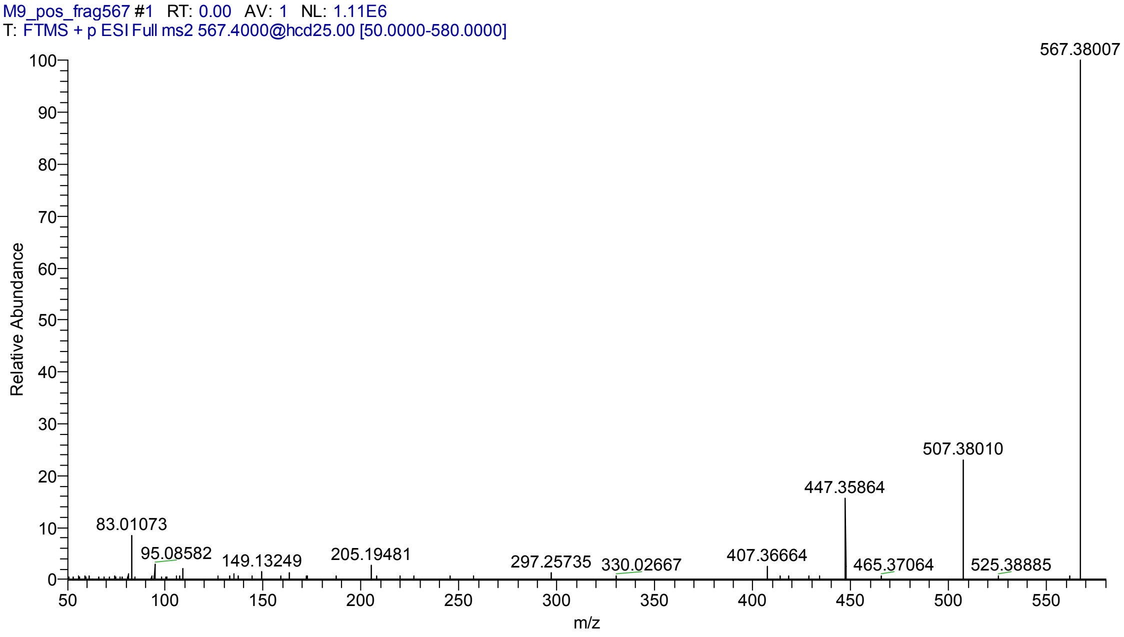

Figure S32: MS^2 spectrum of the 567 *m/z* ion fragmentation (HR-ESI-MS) of the mixture of compounds **3** and **19.**

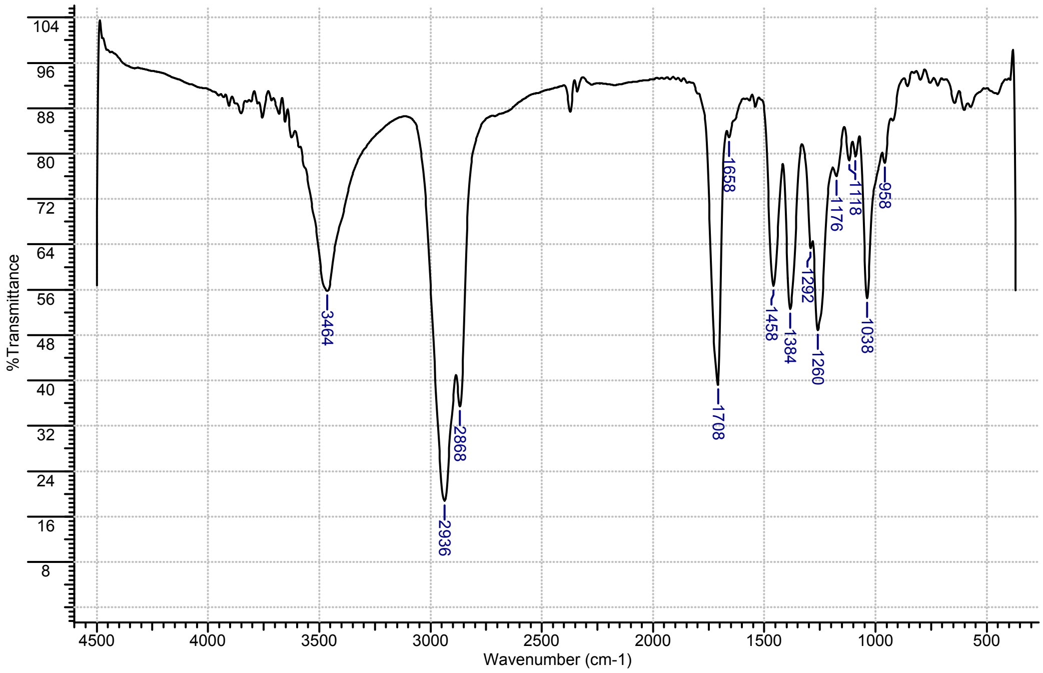

Figure S33: IR spectrum (KBr) of compound **4**.

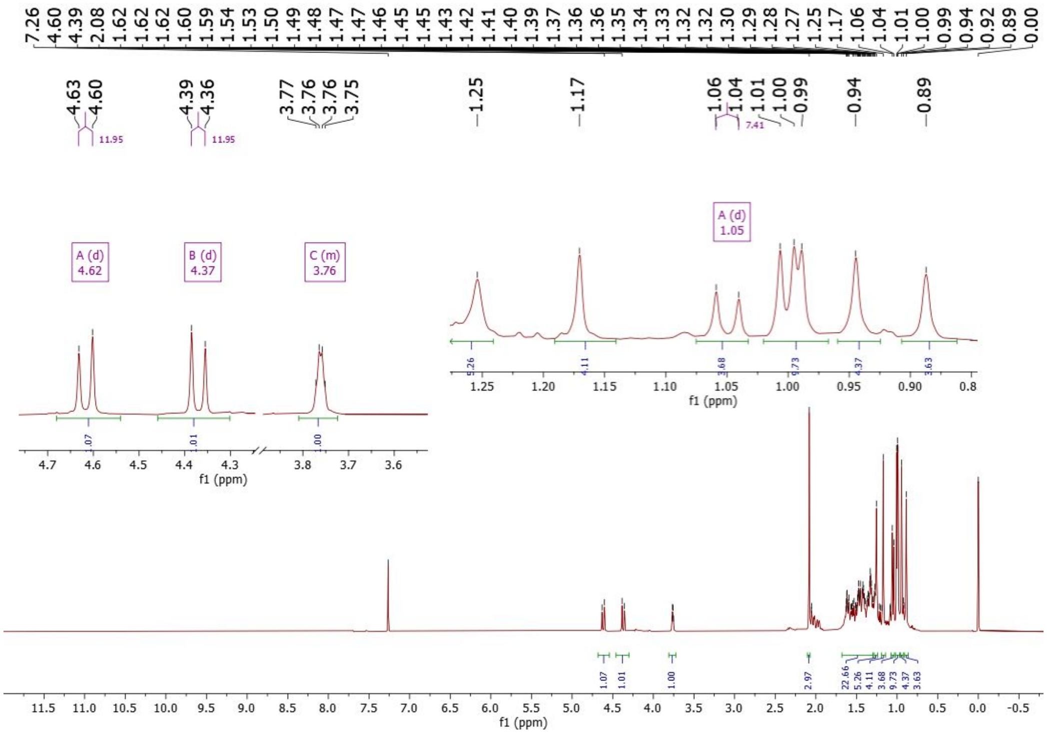

Figure S34: ^{1}H NMR spectrum (400 MHz. $CDCl_3$) of compound **4.**

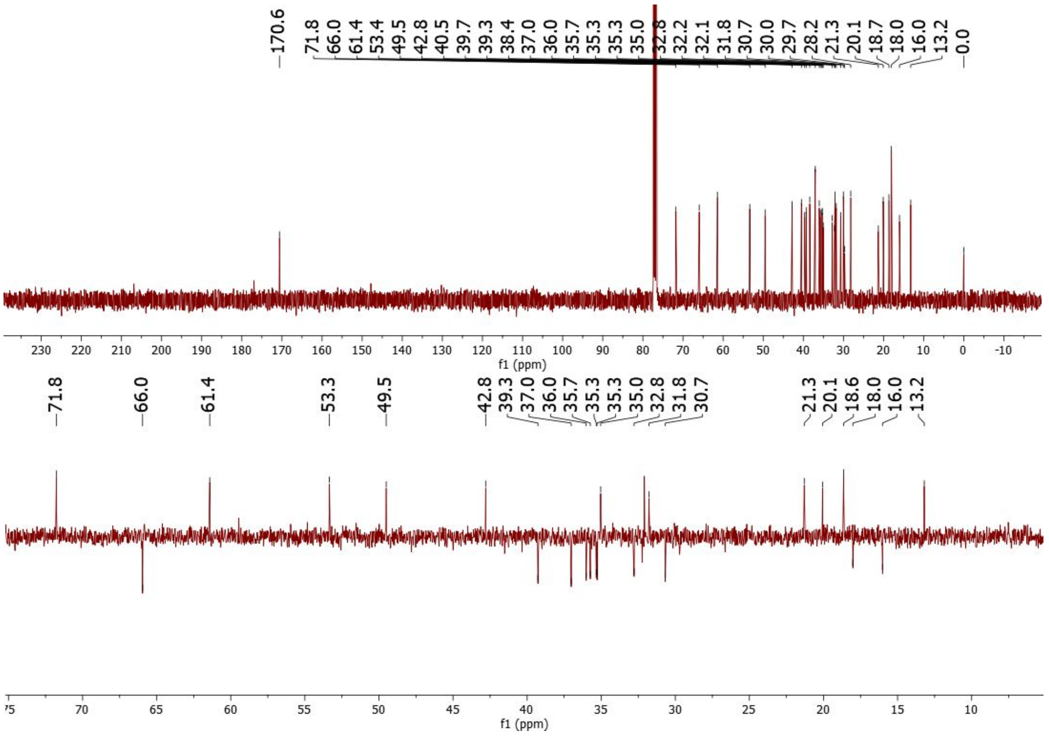

Figure S35: ^{13}C NMR and DEPT-135 spectra (100 MHz. $CDCl_3$) of compound **4**.

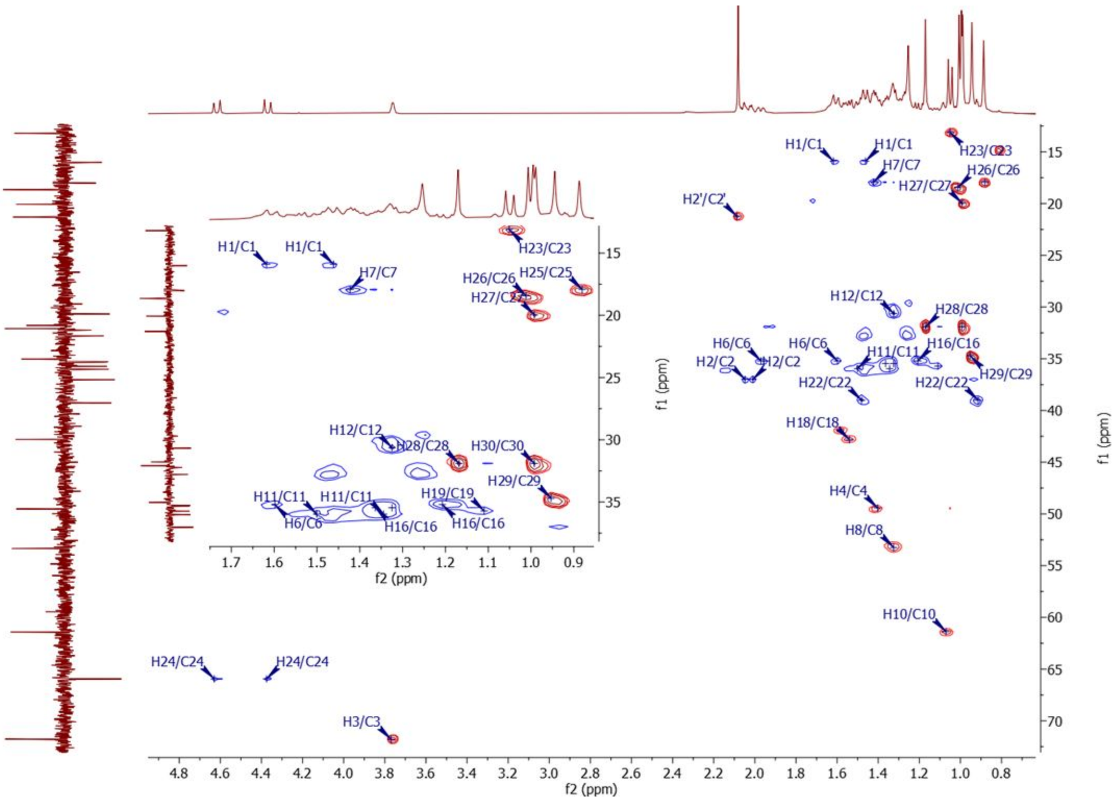

Figure S36: HSQC and expanded HSCQ (400 MHz. $CDCl_3$) spectrum of **4** in the region between δ_H 1.8 to 0.8 ppm.

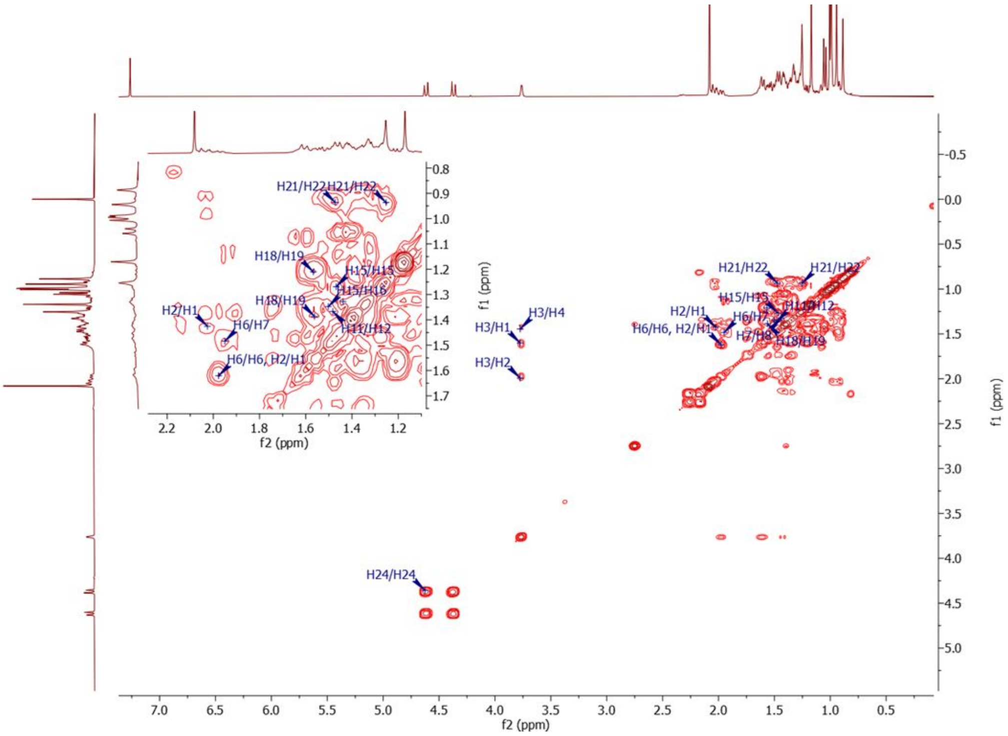

Figure S37: COSY and expanded COSY (400 MHz. $CDCl_3$) spectrum of **4** in the region between δ_H 2.4 to 1.0 ppm.

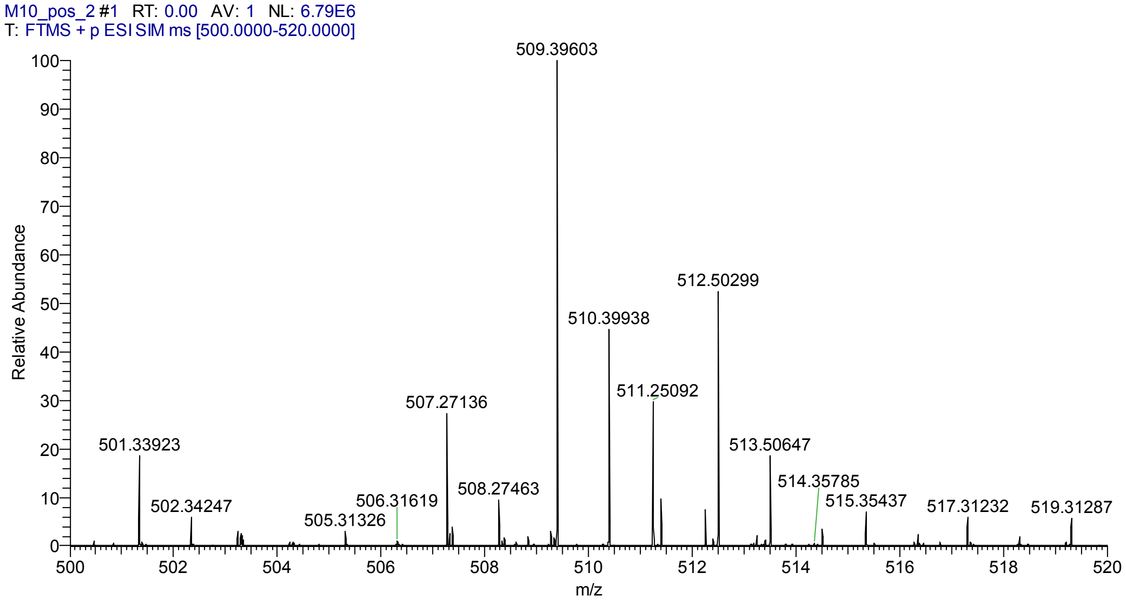

Figure S38: Mass spectrum (HR-ESI-MS) of compound **4**.

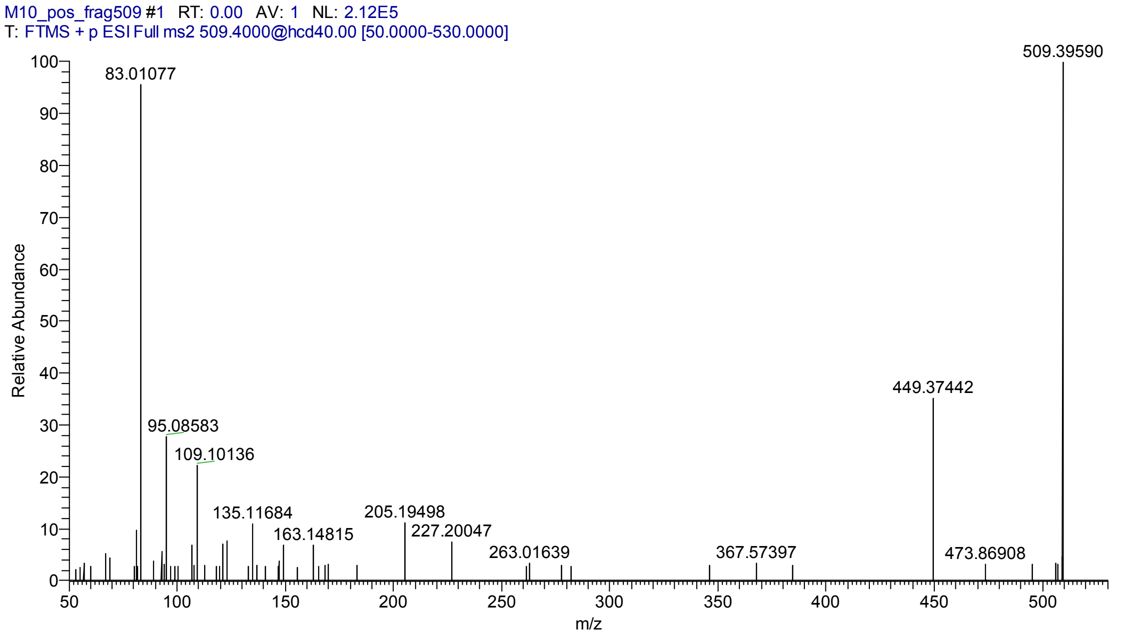

Figure S39: MS^2 spectrum of the 509 *m/z* ion fragmentation (HR-ESI-MS) of compound **4.**

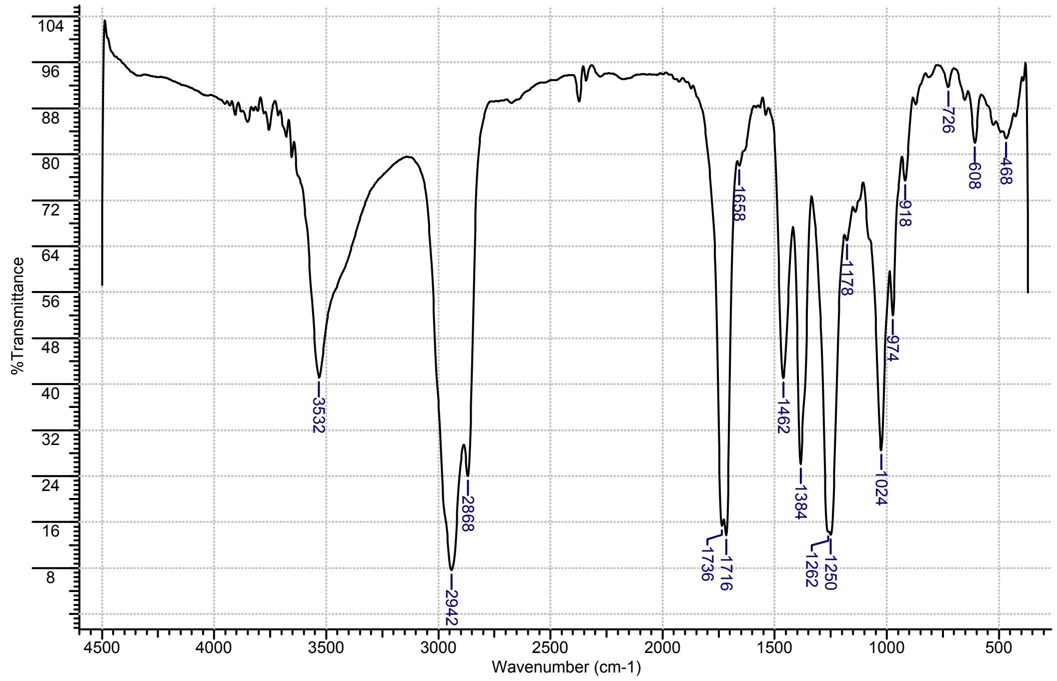

Figure S40: IR spectrum (KBr) of compound **5**.

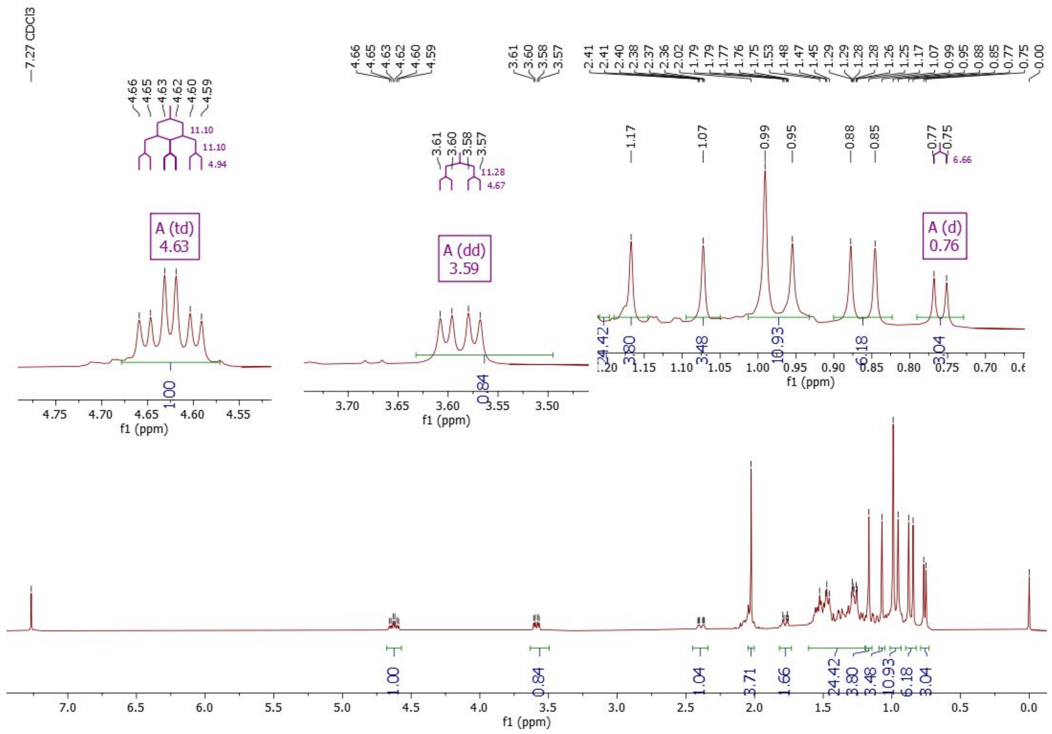

Figure S41: ^{1}H NMR spectrum (400 MHz. $CDCl_3$) of compound **5.**

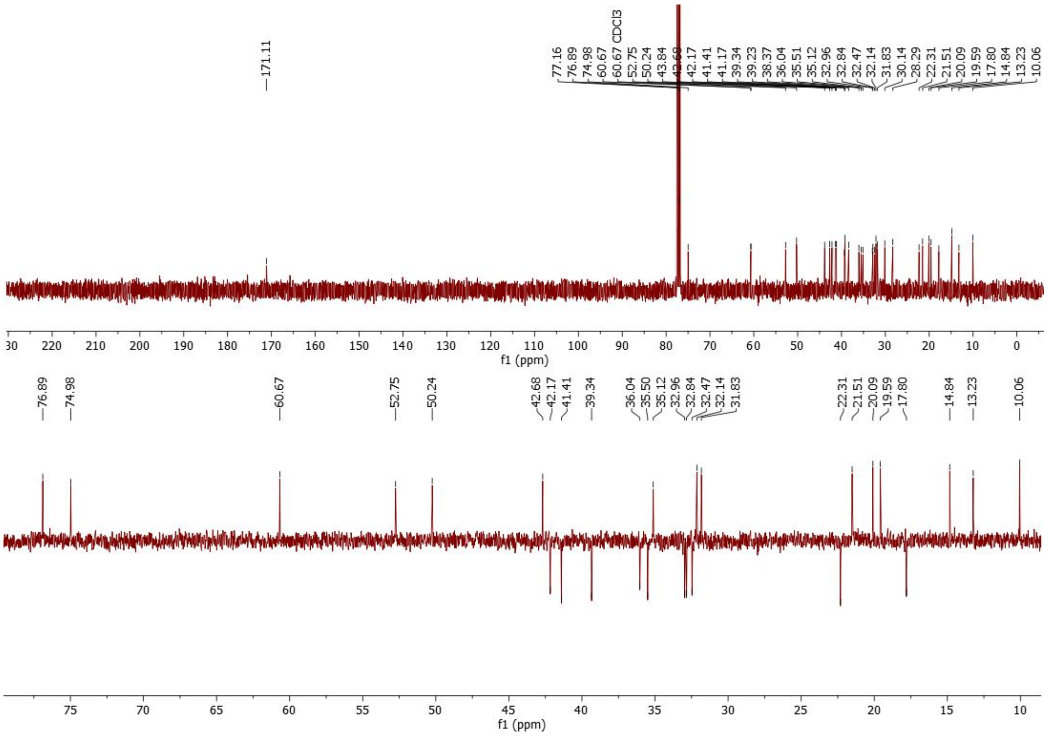

Figure S42: ^{13}C NMR and DEPT-135 spectra (100 MHz. $CDCl_3$) of compound **5.**

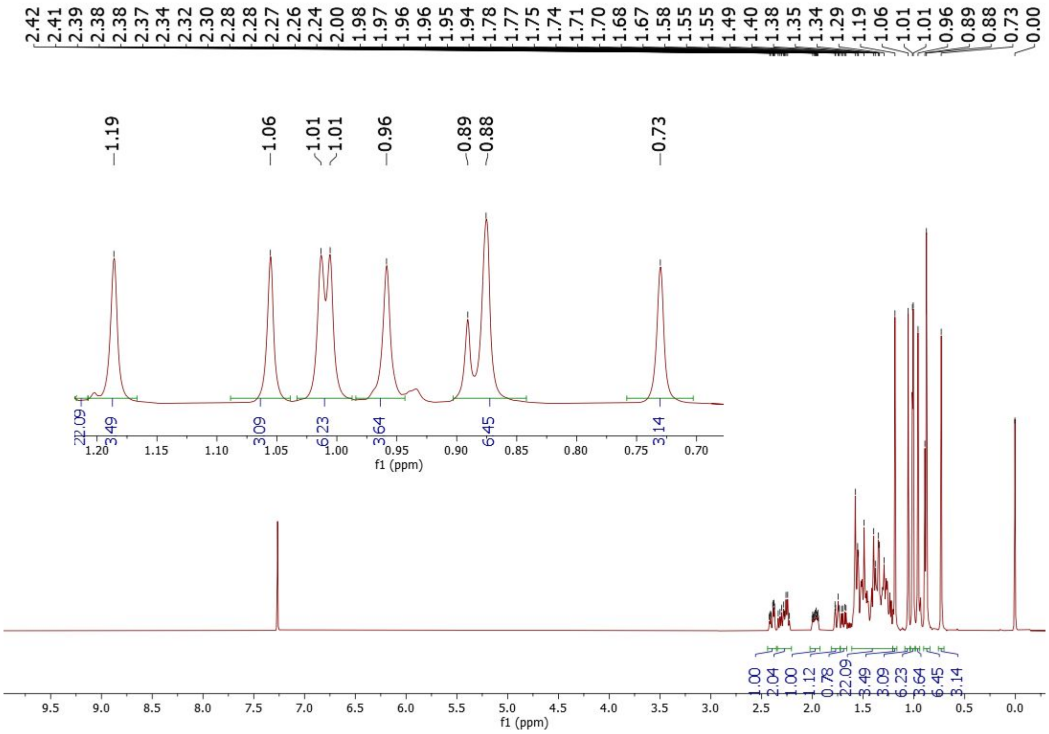

Figure S43: ^{1}H NMR spectrum (400 MHz. $CDCl_3$) of compound **6**.

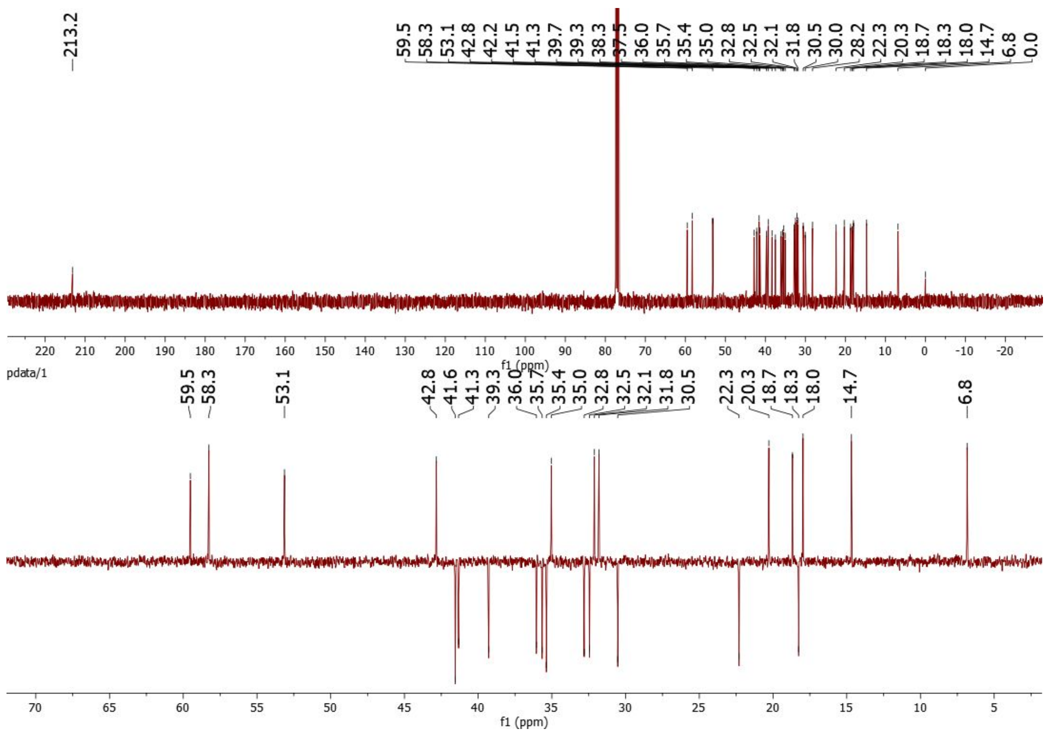

Figure S44: ^{13}C NMR and DEPT-135 spectra (100 MHz. $CDCl_3$) of compound **6**.

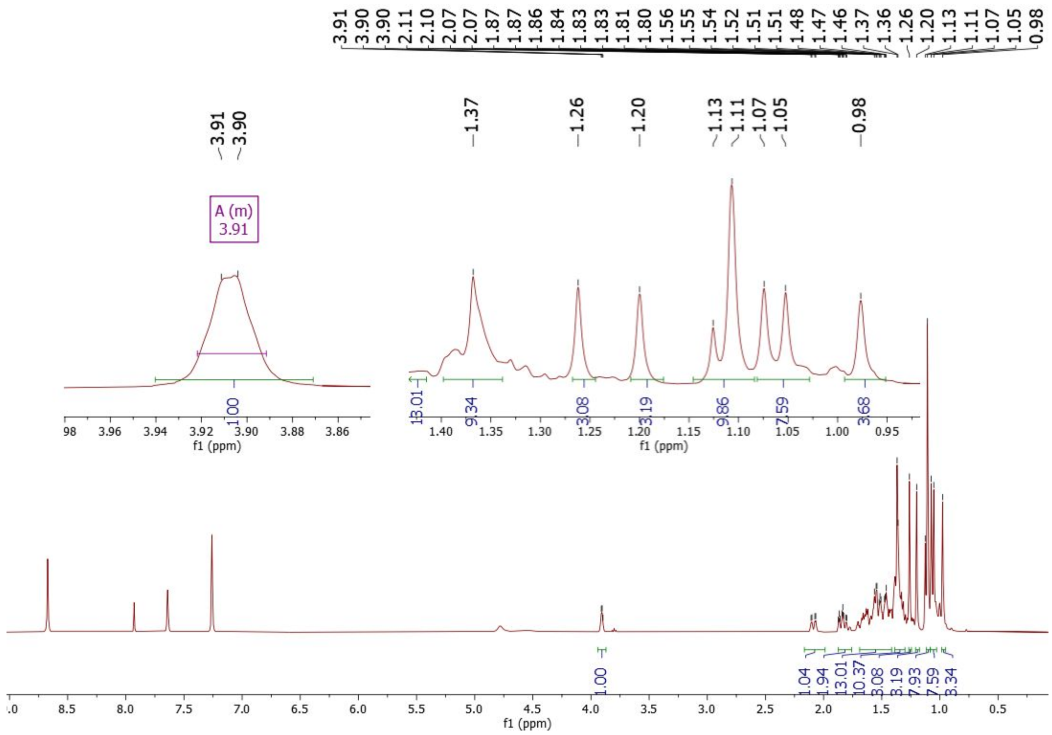

Figure S45: ^{1}H NMR spectrum (400 MHz. $CDCl_3$ + PyD_5) of compound **7**.

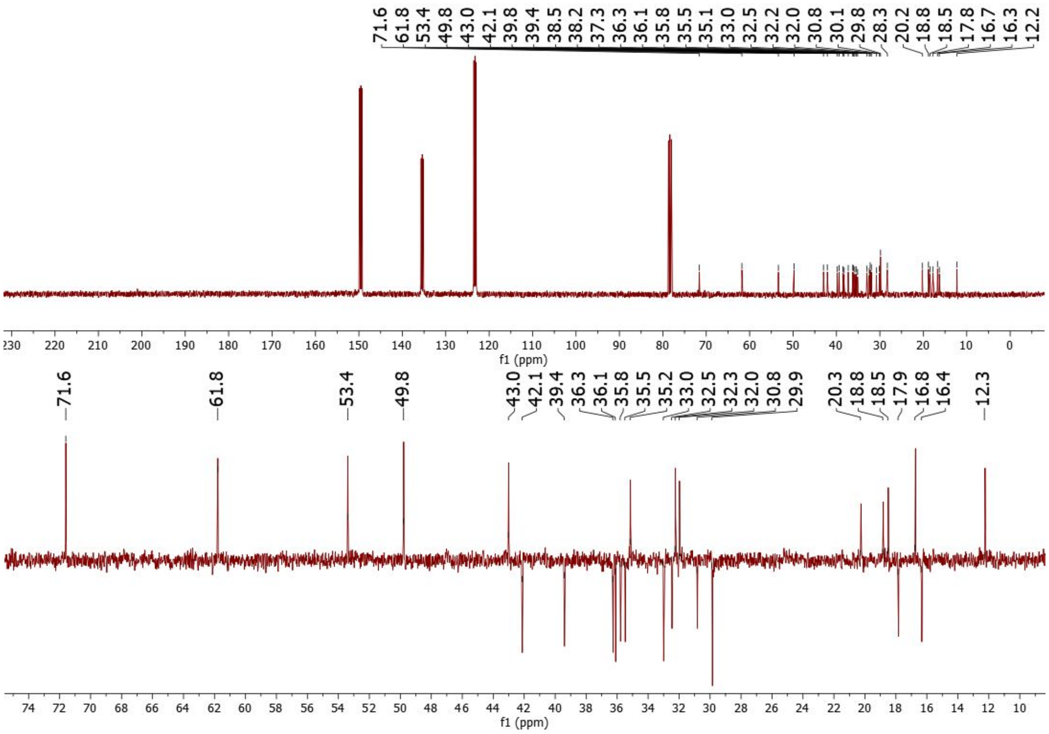

Figure S46: ^{13}C NMR and DEPT-135 spectra (100 MHz. $CDCl_3$+ PyD_5) of compound **7**.

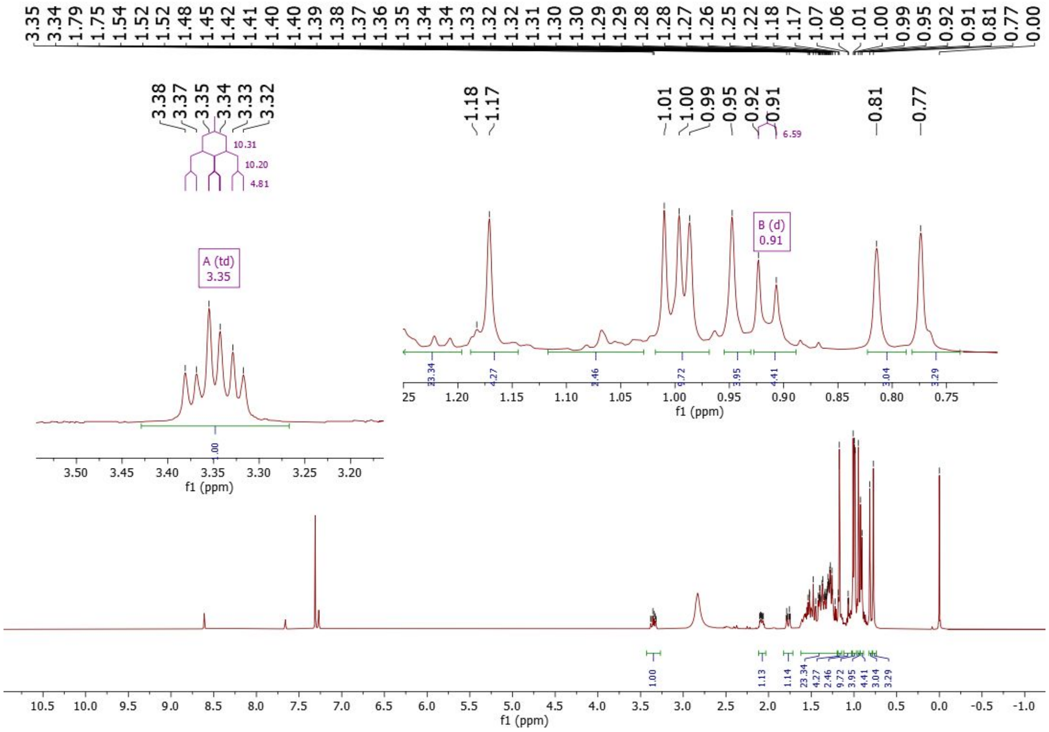

Figure S47: ^{1}H NMR spectrum (400 MHz. $CDCl_3$ + PyD_5) of compound **8**.

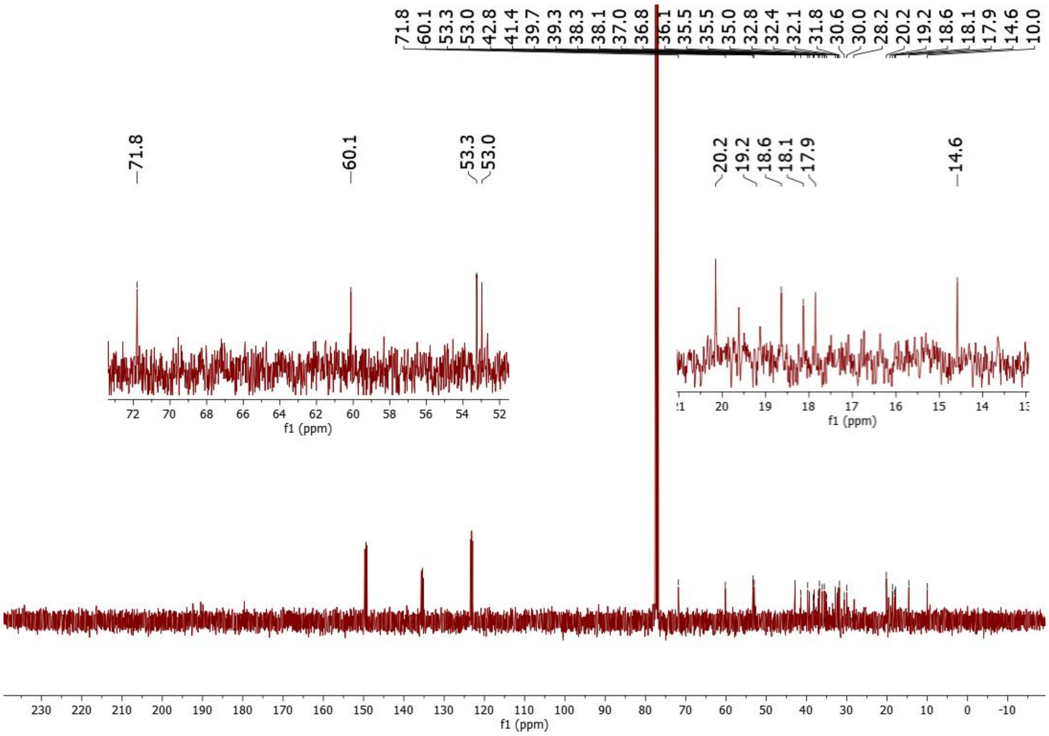

Figure S48: ^{13}C NMR spectrum (100 MHz. $CDCl_3$ + PyD_5) of compound **8**.

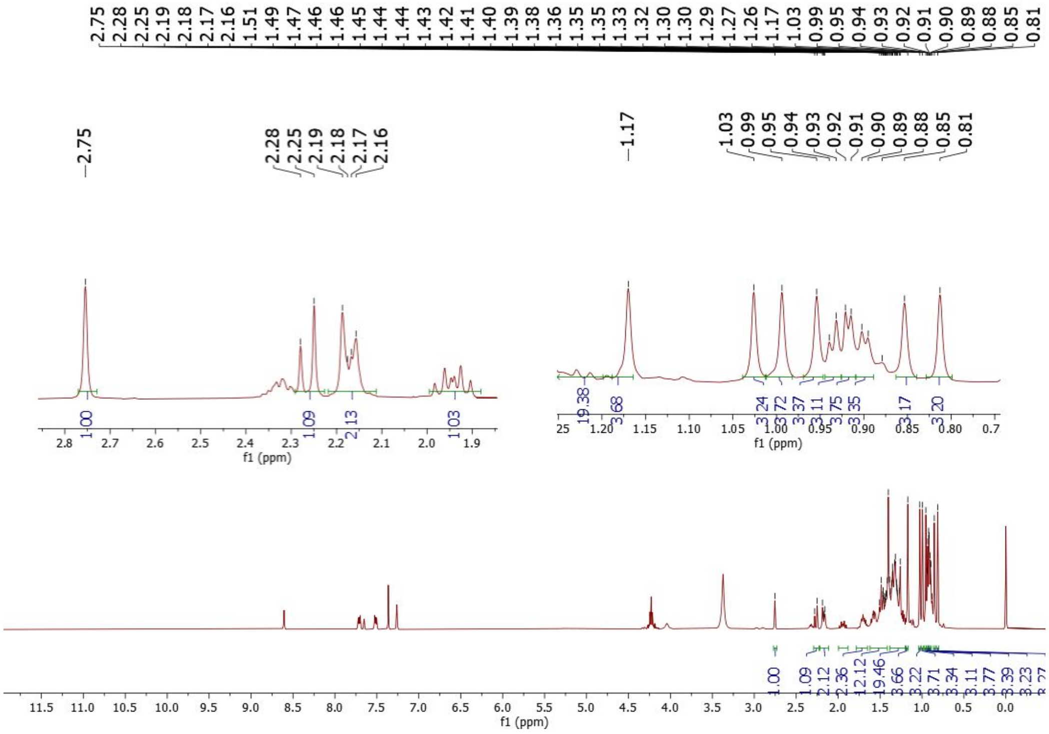

Figure S49: ^{1}H NMR spectrum (400 MHz. $CDCl_3$) of compound **9**.

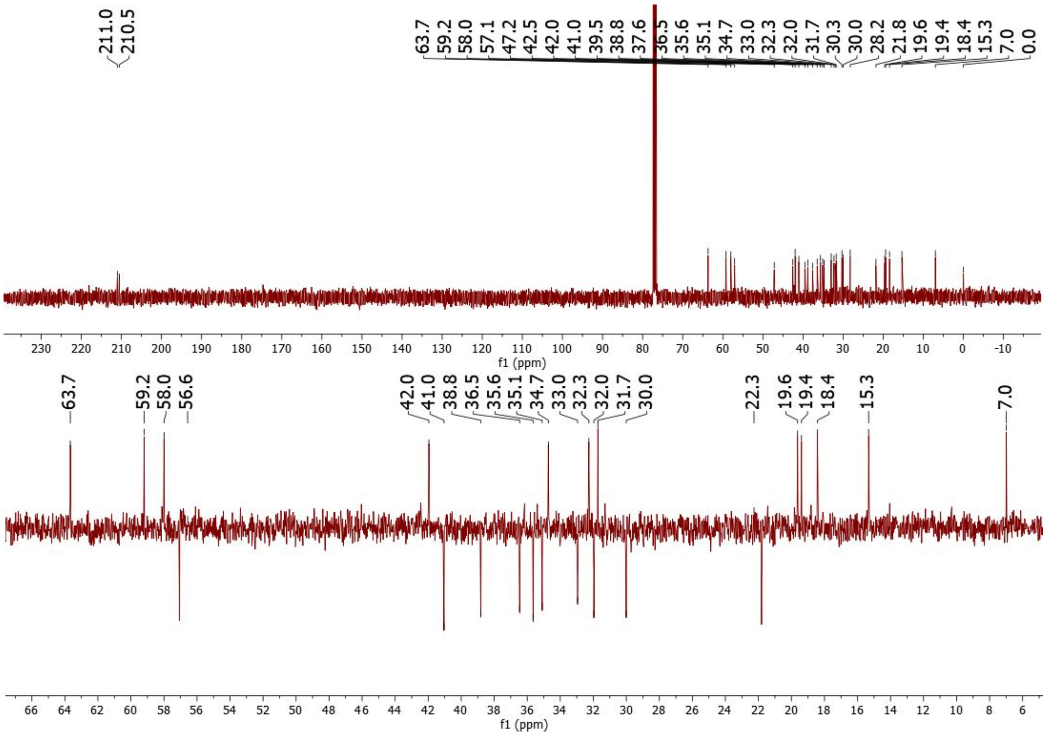

Figure S50: ^{13}C NMR and DEPT-135 spectra (100 MHz. $CDCl_3$) of compound **9**.

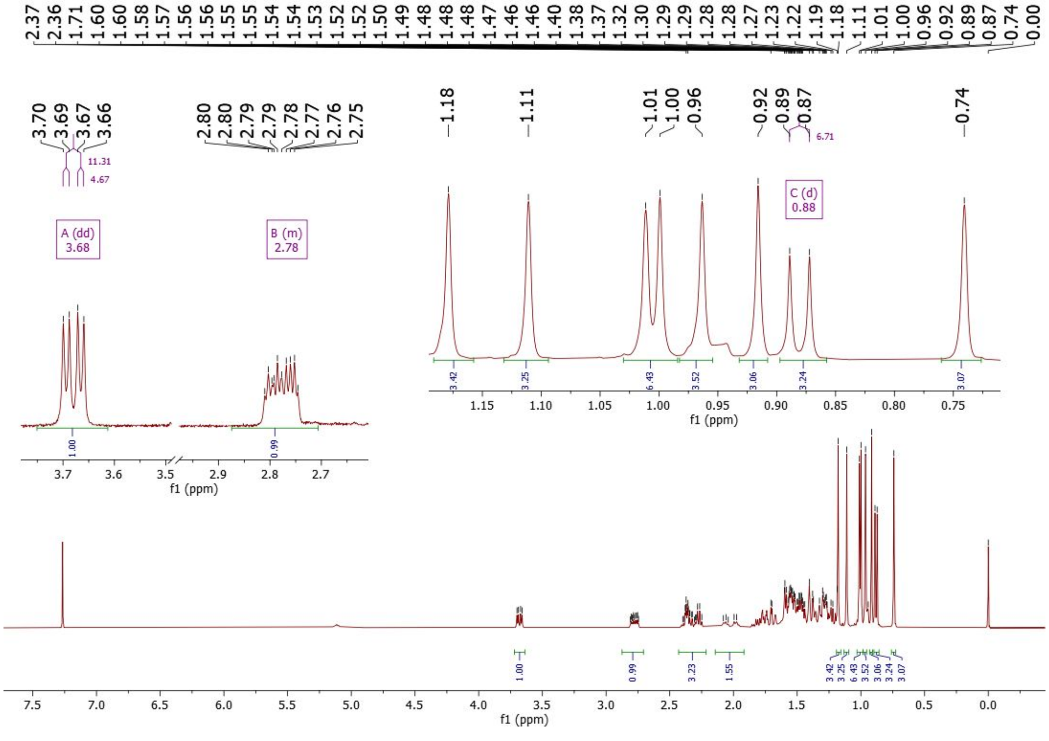

Figure S51: ^{1}H NMR spectrum (400 MHz. $CDCl_3$) of compound **10**.

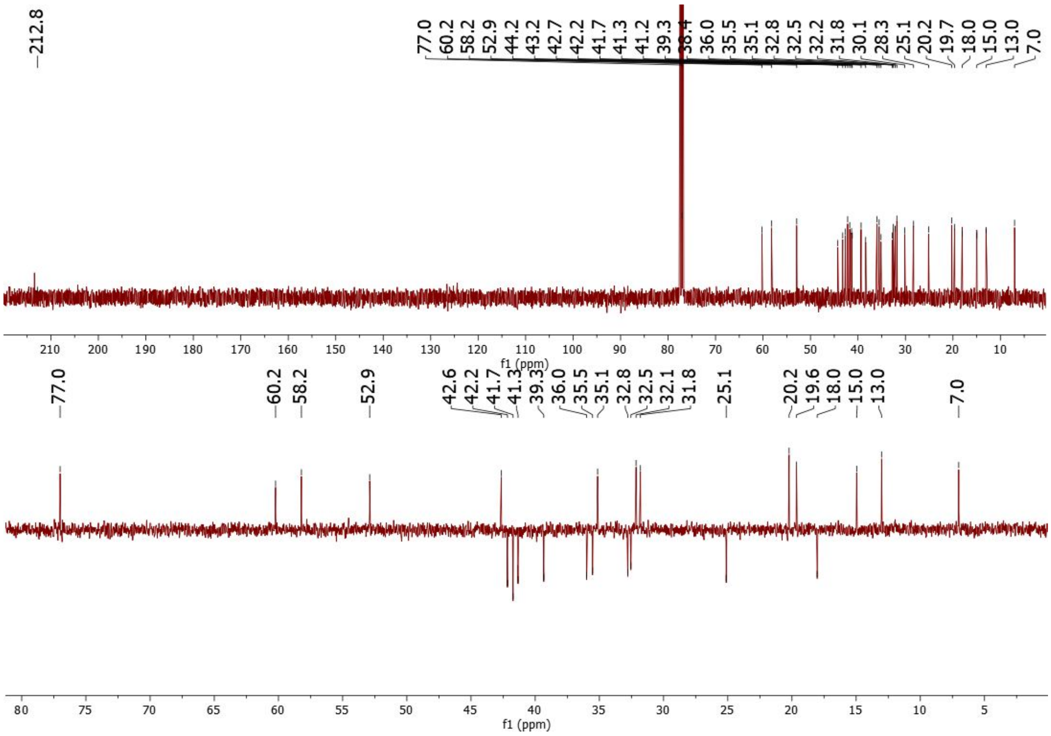

Figure S52: ^{13}C NMR and DEPT-135 spectra (100 MHz. $CDCl_3$) of compound **10**.

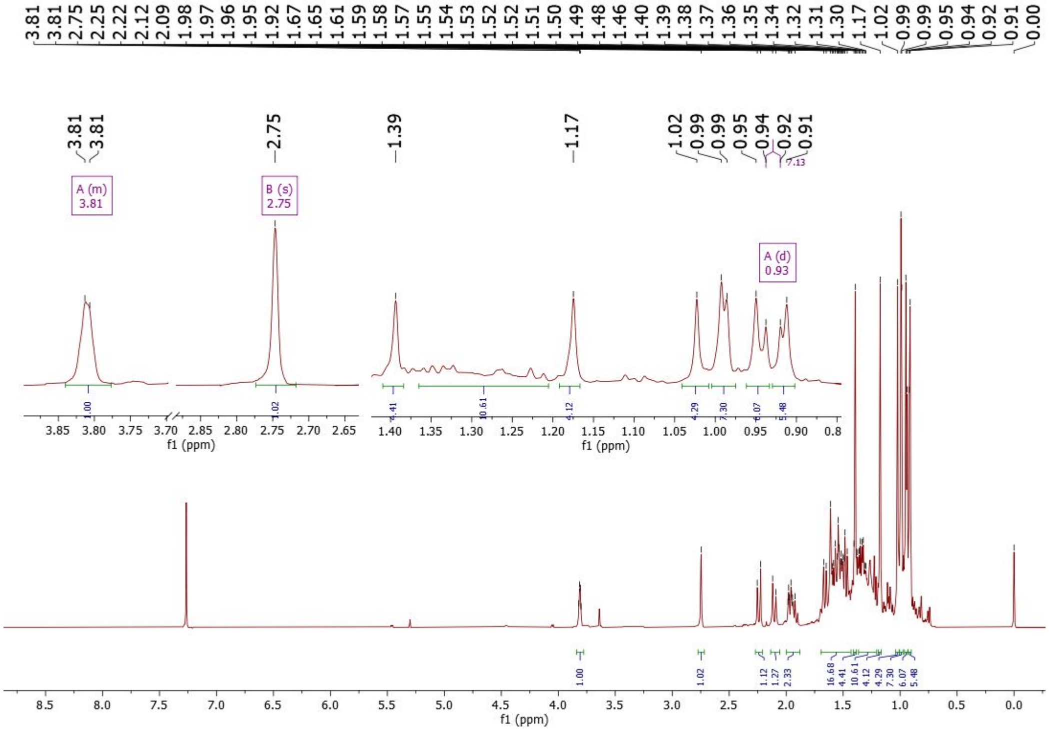

Figure S53: ^{1}H NMR spectrum (400 MHz. $CDCl_3$) of compound **11**.

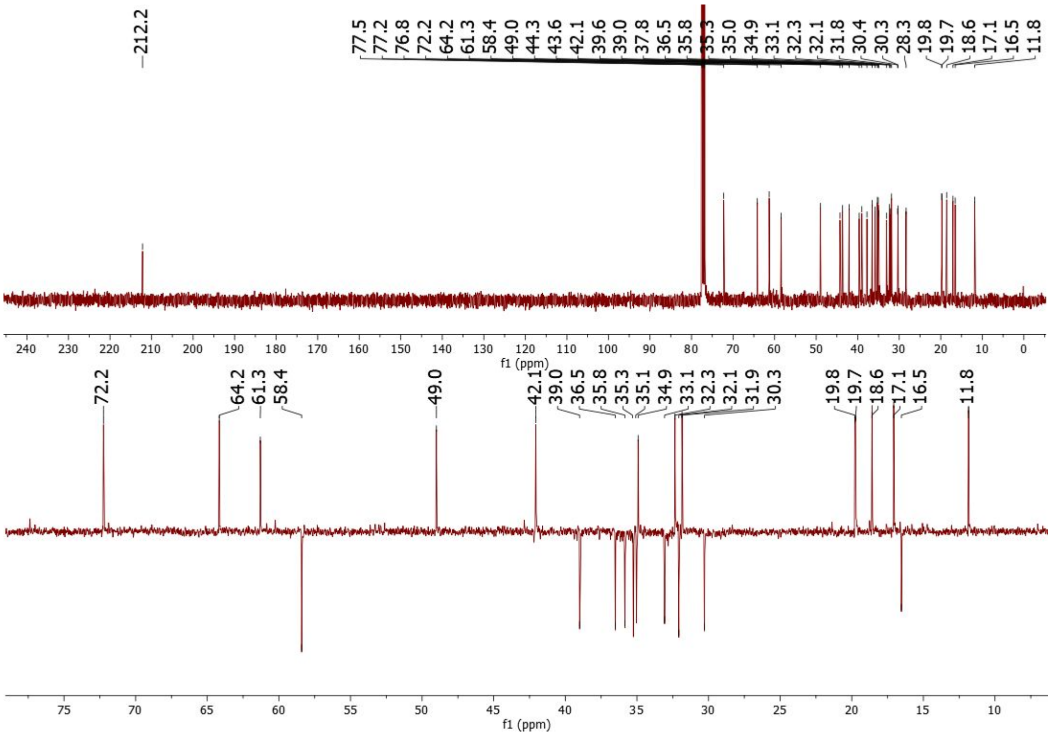

Figure S54: ^{13}C NMR and DEPT-135 spectra (100 MHz. $CDCl_3$) of compound **11**.

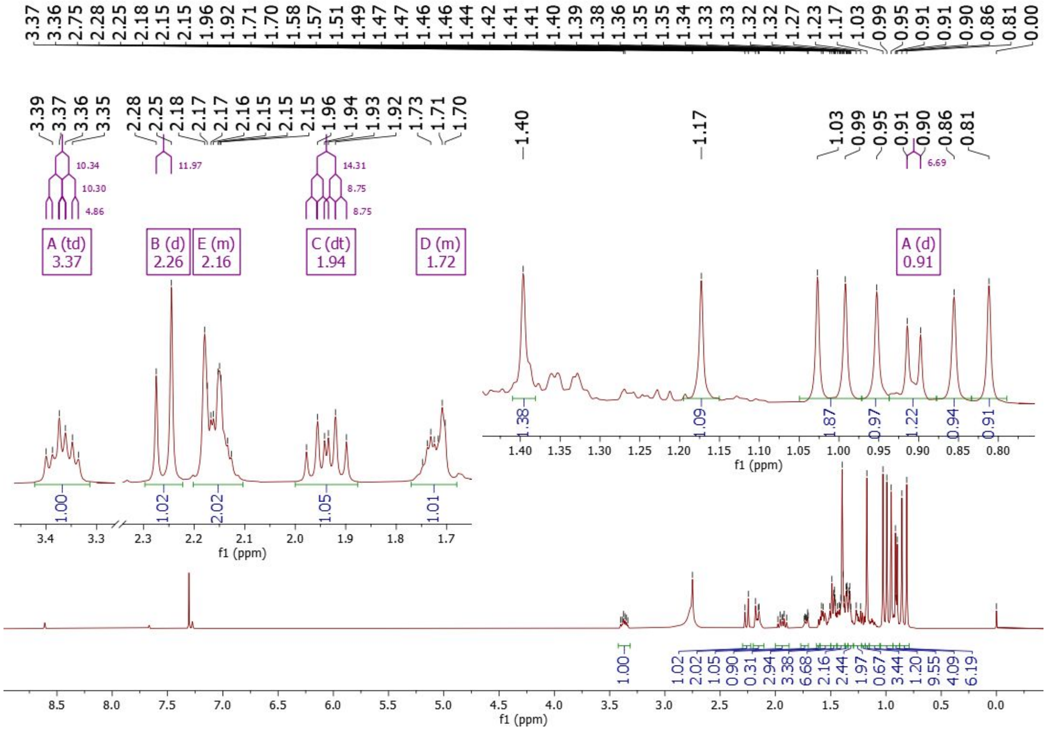

Figure S55: ^{1}H NMR spectrum (400 MHz. $CDCl_3$) of compound **12.**

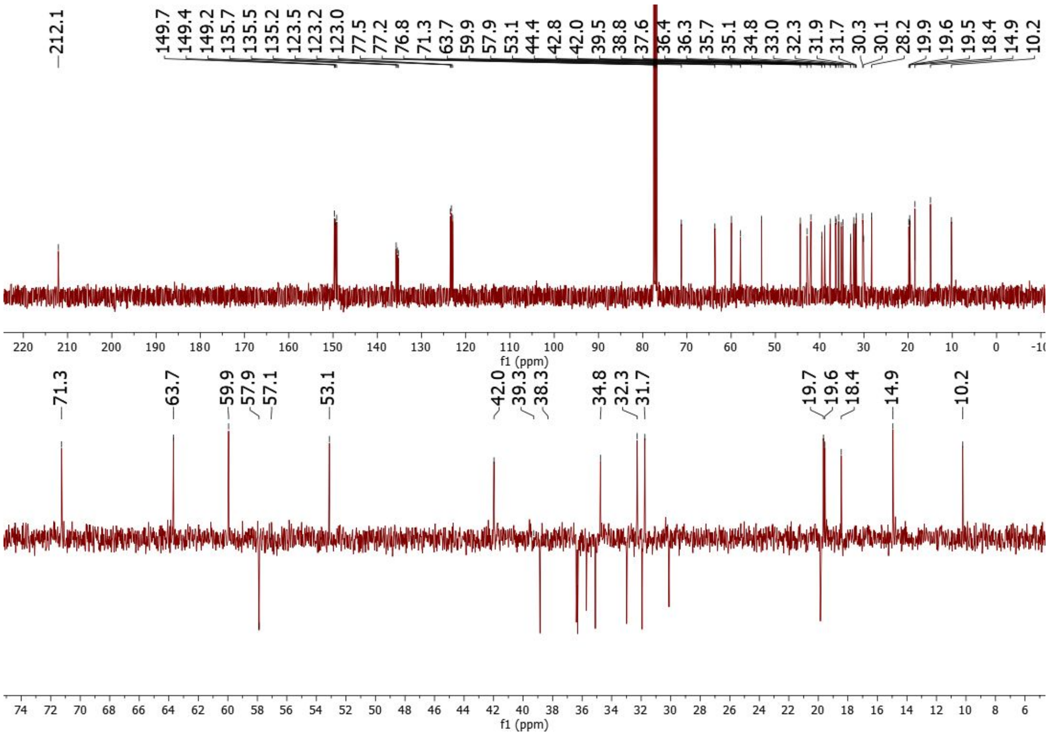

Figure S56: ^{13}C NMR and DEPT-135 spectra (100 MHz. $CDCl_3$) of compound **12**.

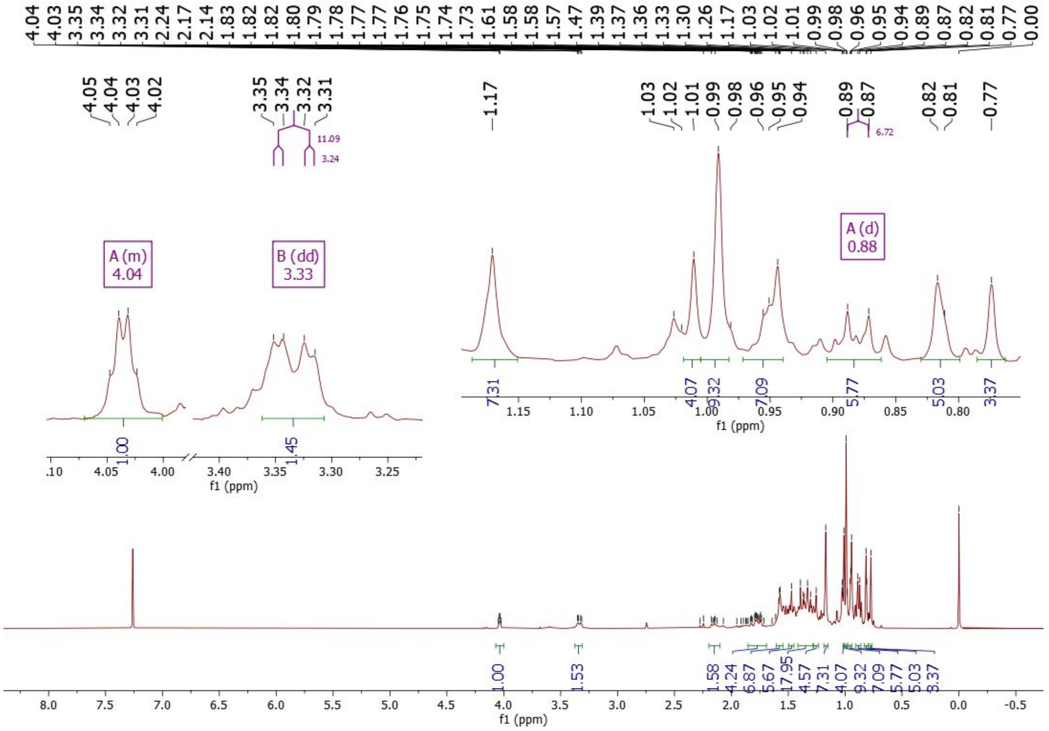

Figure S57: ^{1}H NMR spectrum (400 MHz. $CDCl_3$) of compound **13.**

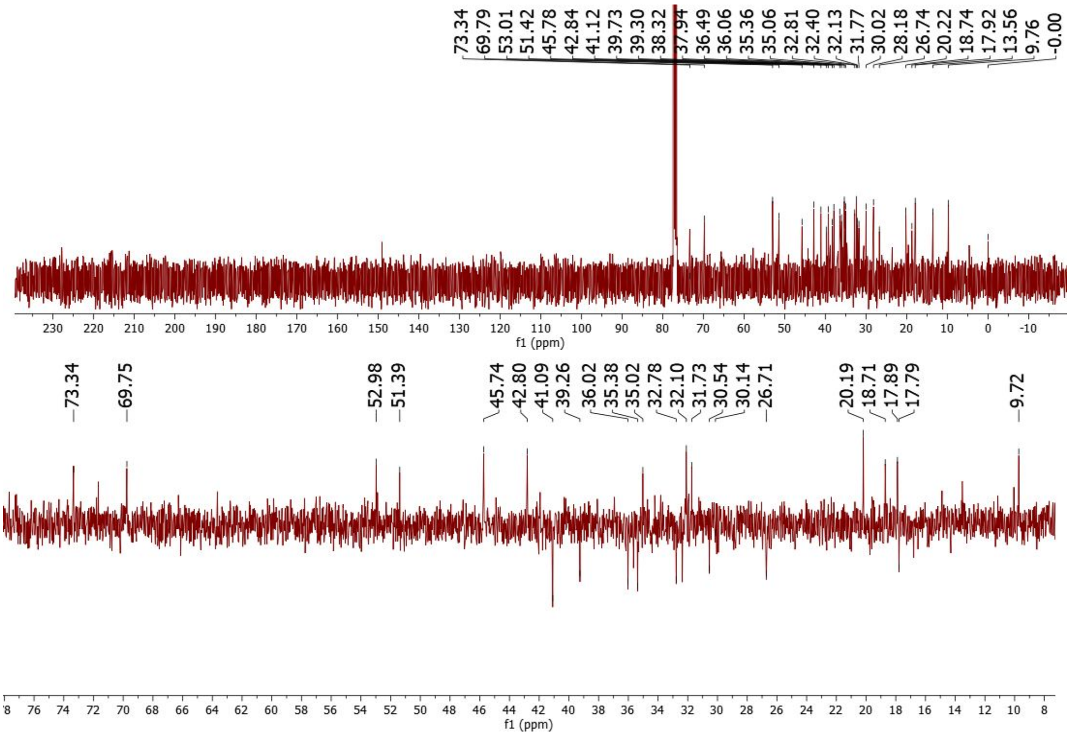

Figure S58: ^{13}C NMR and DEPT-135 spectra (100 MHz. $CDCl_3$) of compound **13.**

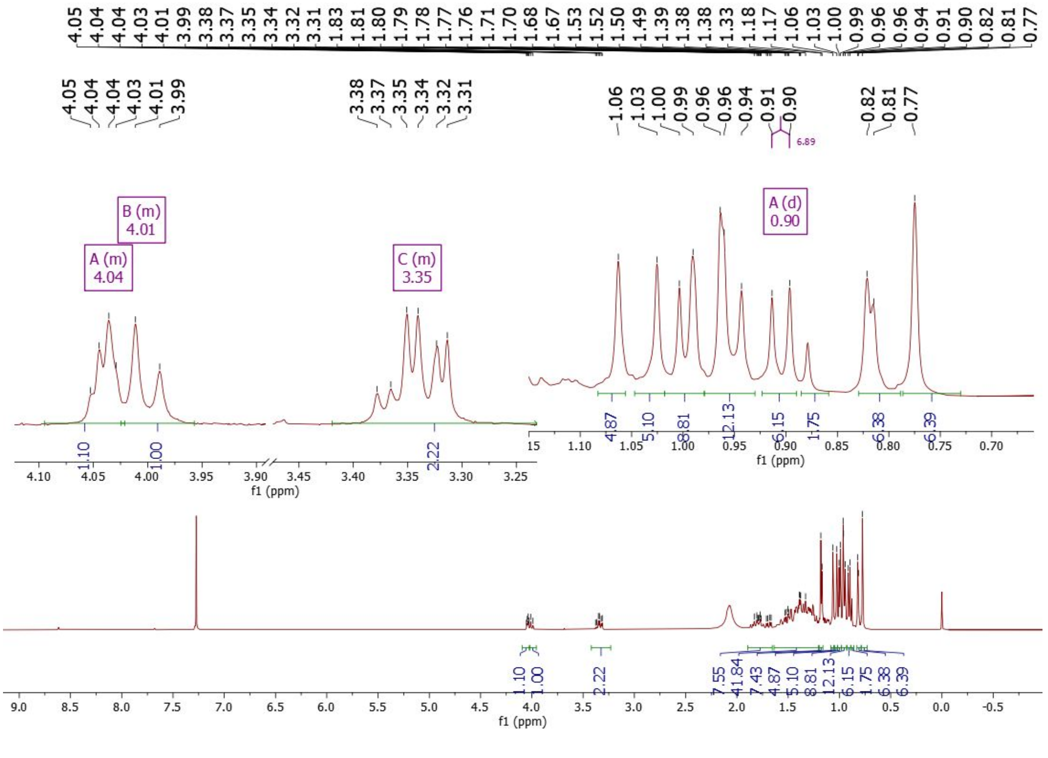

Figure S59: ^{1}H NMR spectrum (400 MHz. $CDCl_3 + PyD_5$) of the mixture of compounds **13** and **18**.

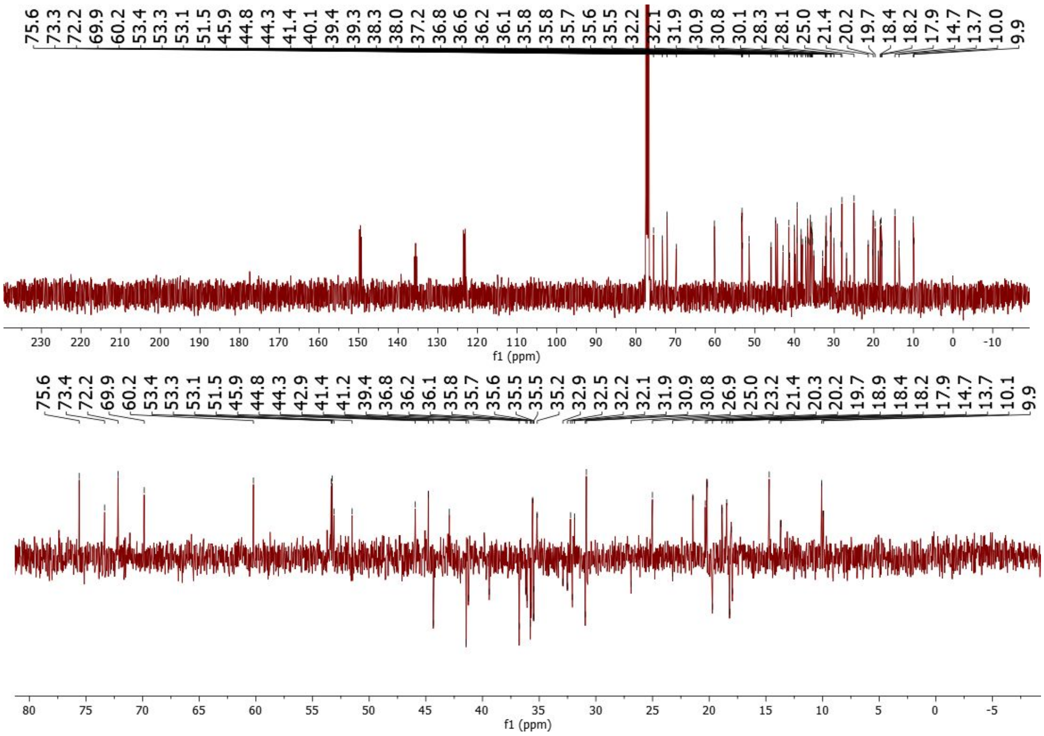

Figure S60: ^{13}C NMR and DEPT-135 spectra (100 MHz. $CDCl_3$ + PyD_5) of the mixture of compounds **13** and **18.**

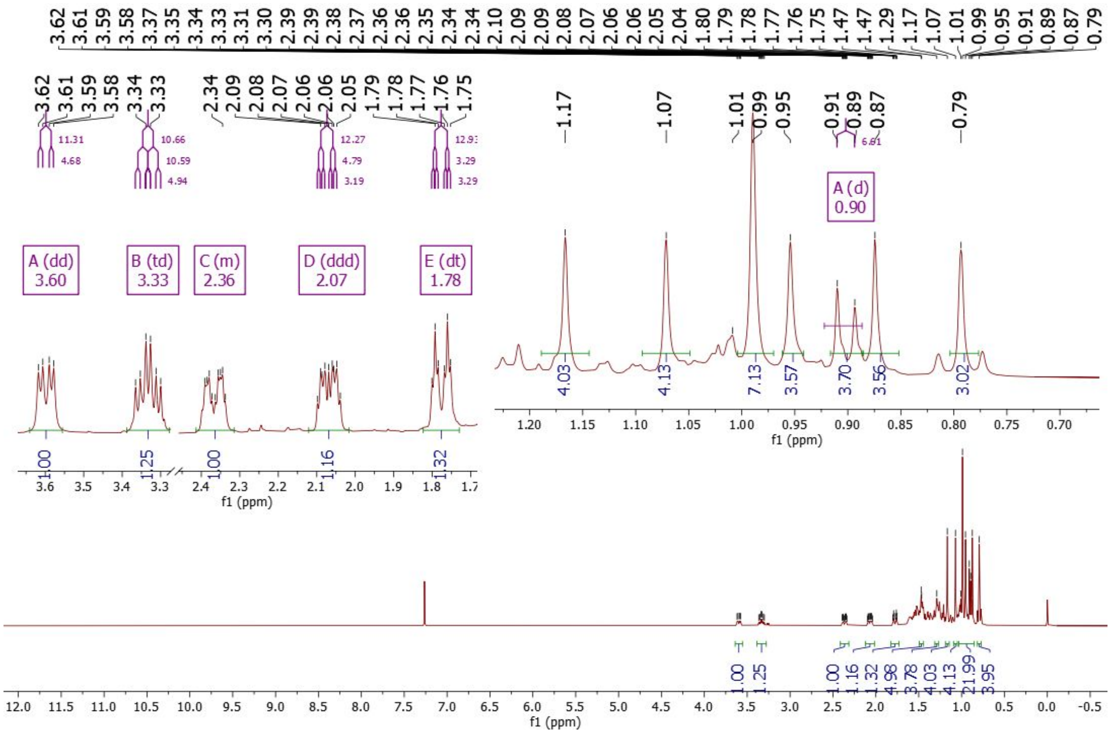

Figure S61: ^{1}H NMR spectrum (400 MHz. $CDCl_3$) of compound **14.**

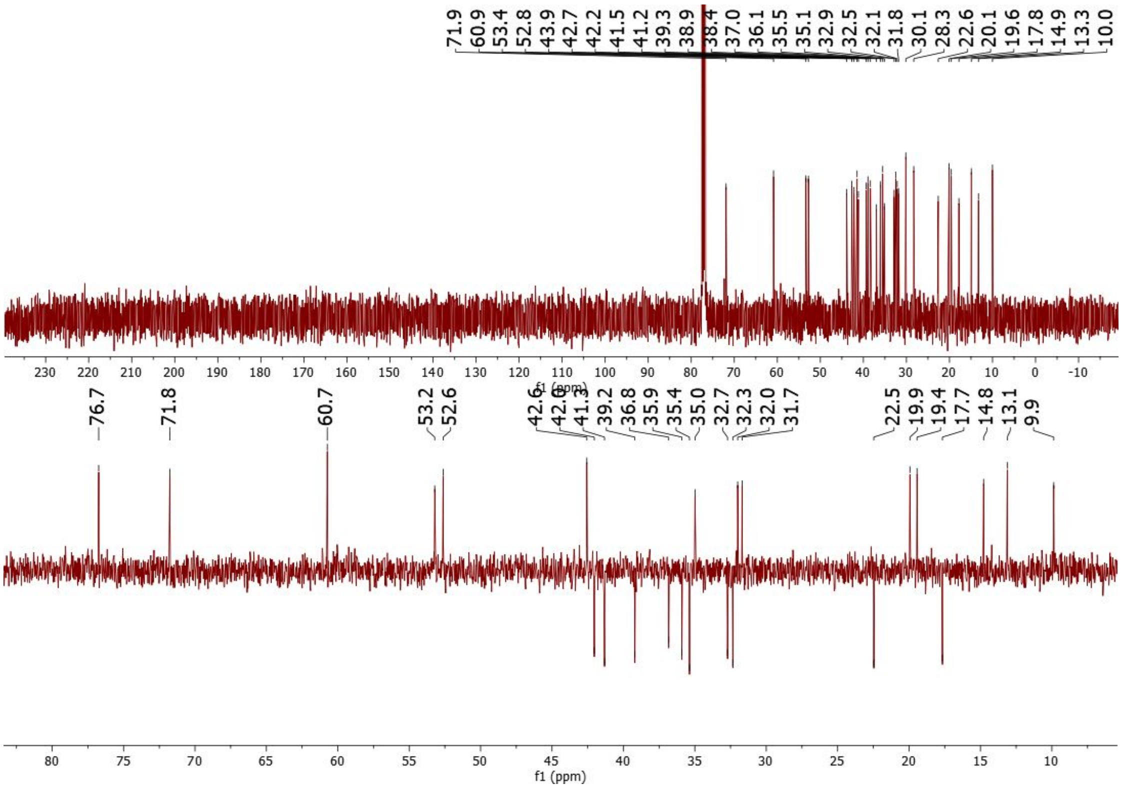

Figure S62: ^{13}C NMR and DEPT-135 spectra (100 MHz. $CDCl_3$) of compound **14.**

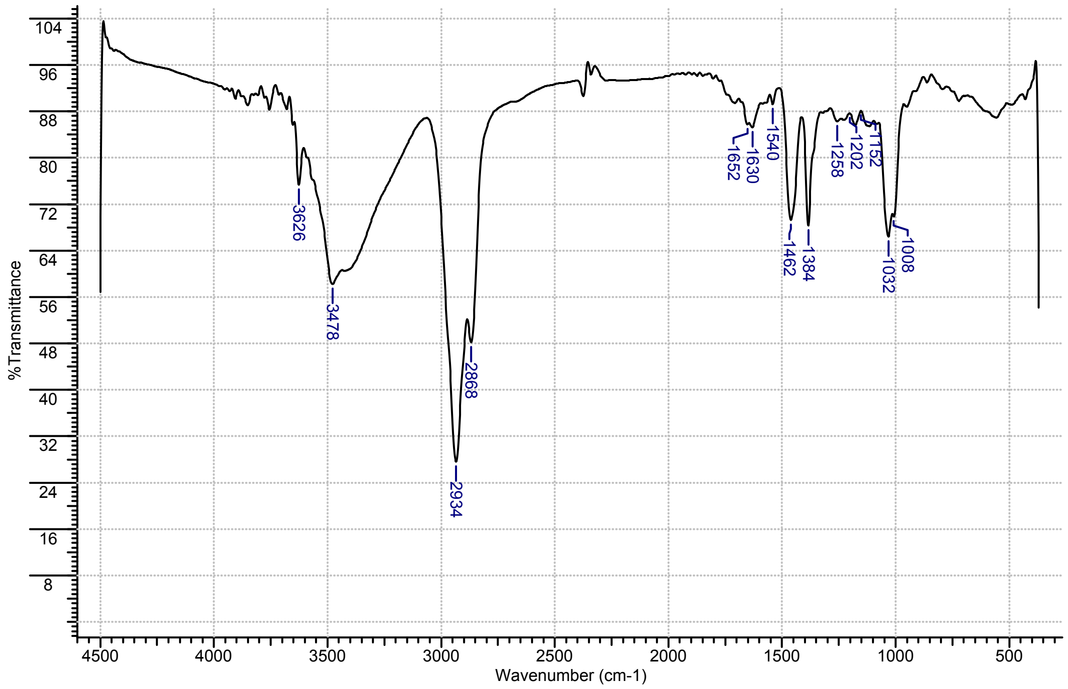

Figure S63: IR spectrum (KBr) of compound **15**.

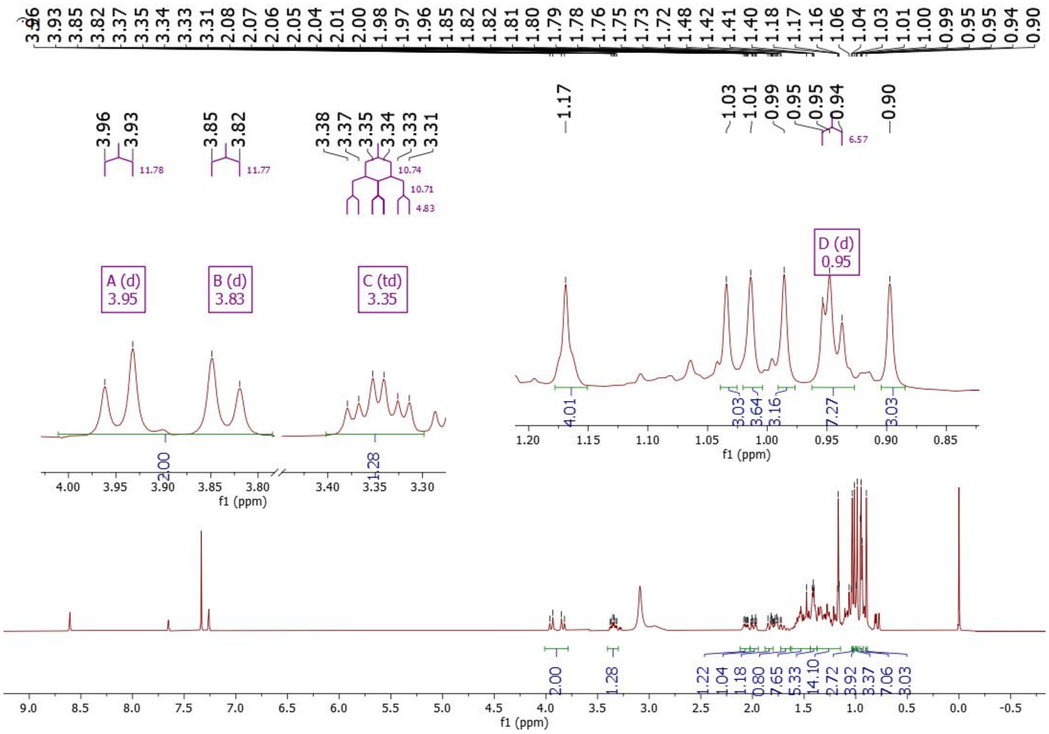

Figure S64: ^{1}H NMR spectrum (400 MHz. $CDCl_3$ + PyD_5) of compound **15.**

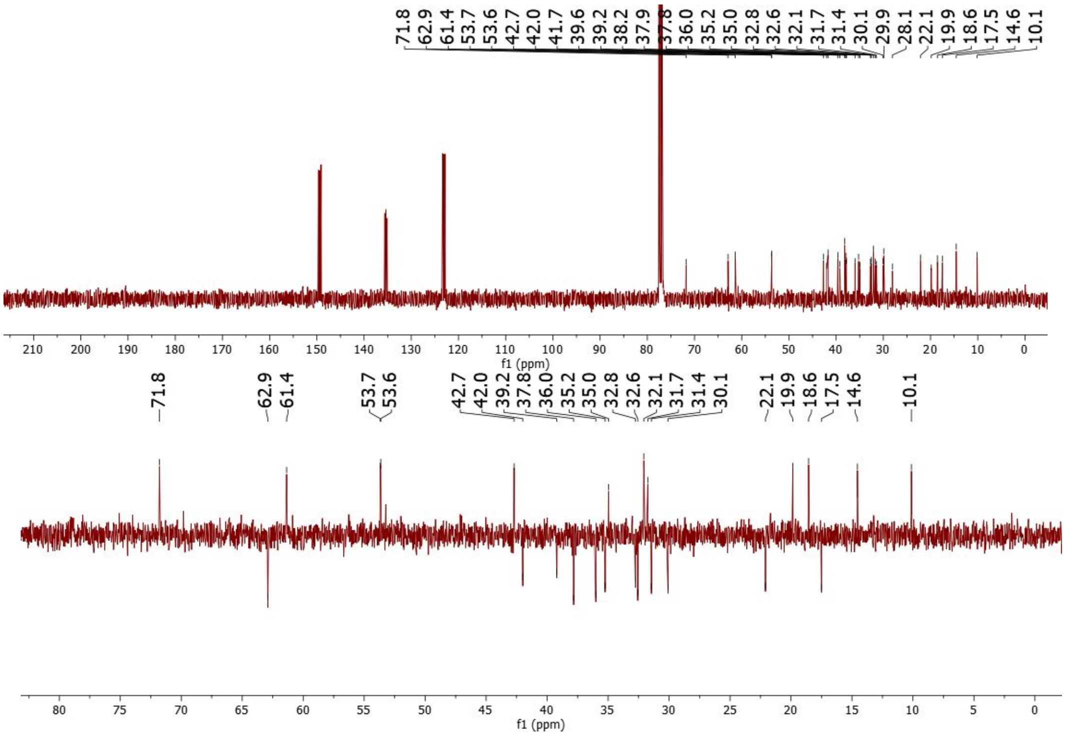

Figure S65: ^{13}C NMR and DEPT-135 spectra (100 MHz. $CDCl_3$ + PyD_5) of compound **15.**

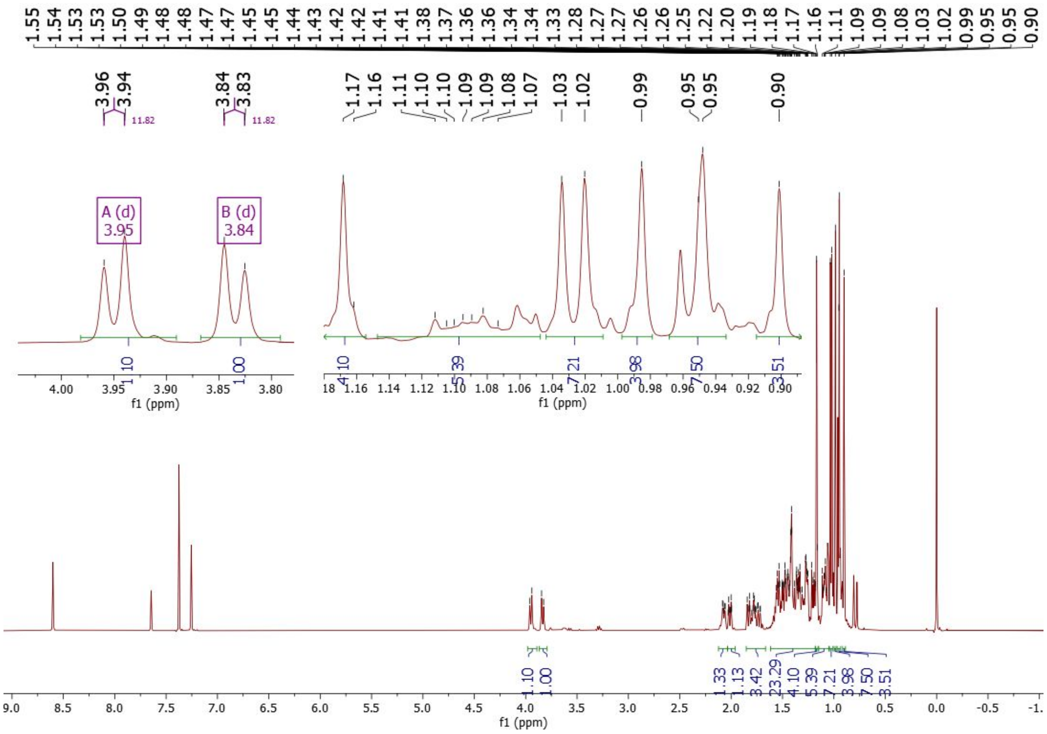

Figure S66: ^{1}H NMR spectrum (600 MHz. $CDCl_3$ + PyD_5) of compound **15.**

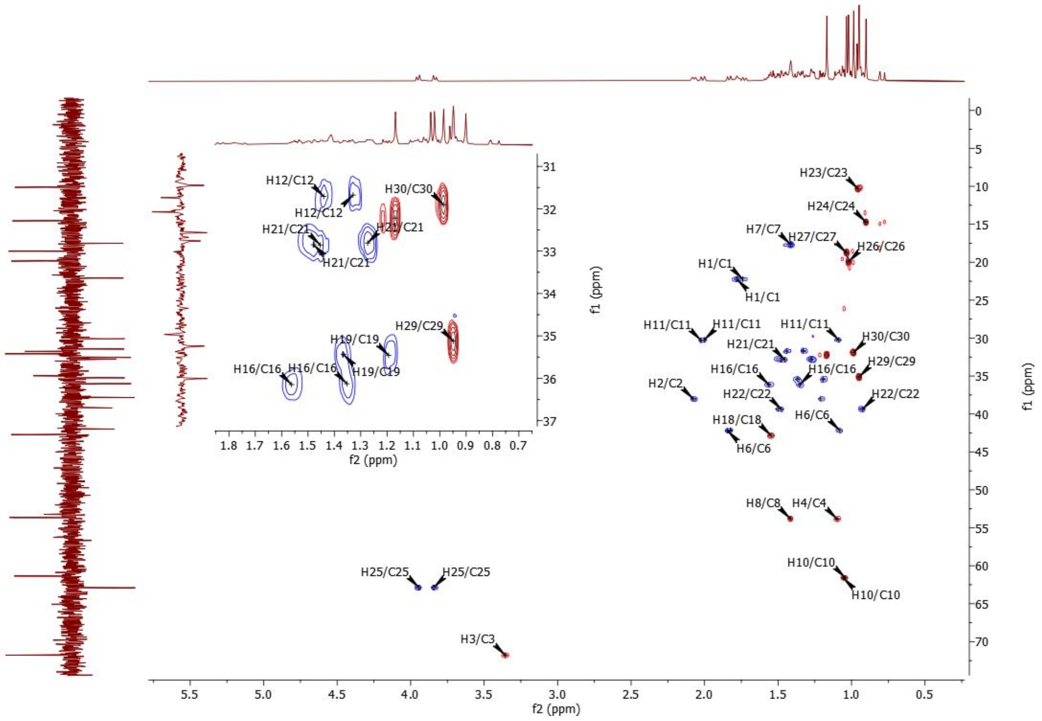

Figure S67: HSQC and expanded HSQC (600 MHz. $CDCl_3$ + PyD_5) spectrum of **15** in the region between δ_H 1.8 to 0.7 ppm.

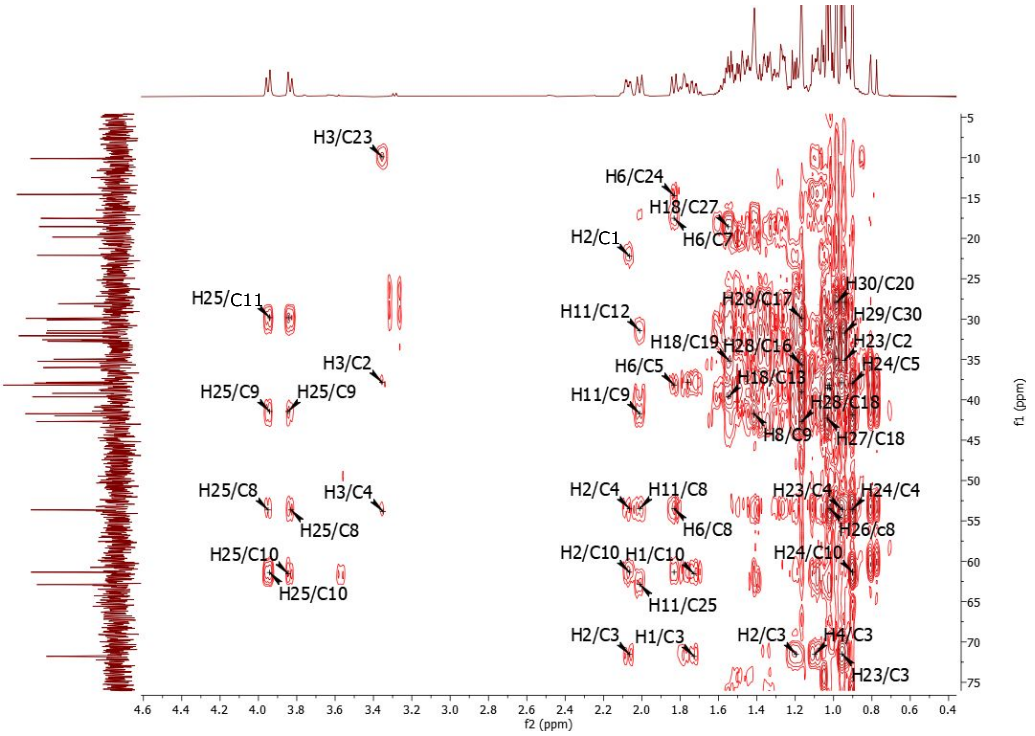

Figure S68: HMBC (600 MHz. $CDCl_3$ + PyD_5) spectrum of **15**.

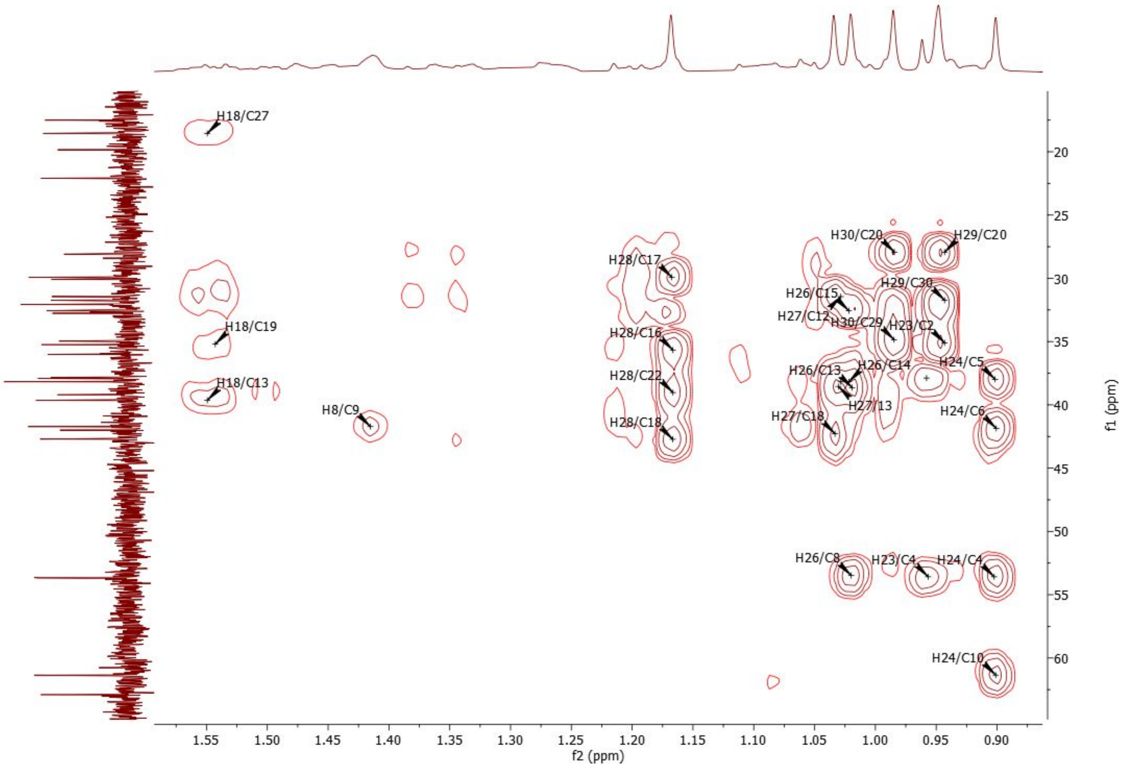

Figure S69: Expanded HMBC (600 MHz. $CDCl_3$ + PyD_5) spectrum of **15** in the region between δ_H 1.6 to 0.8 ppm.

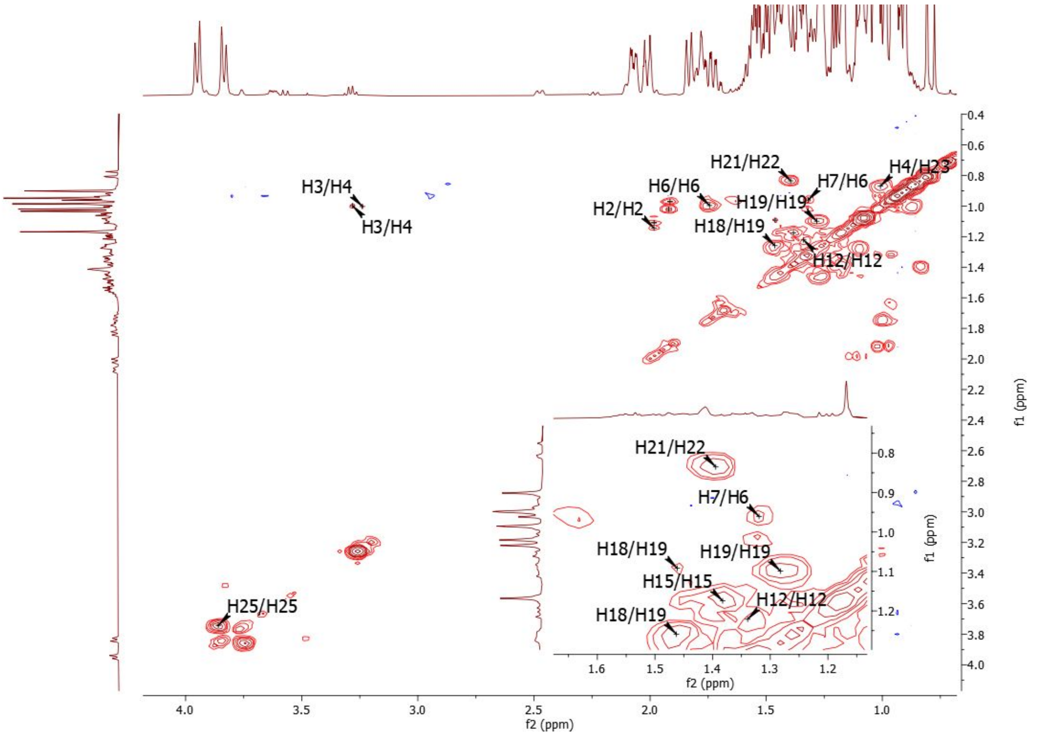

Figure S70: COSY and expanded COSY (600 MHz. $CDCl_3 + PyD_5$) spectrum of **15** in the region between δ_H 1.7 to 1.1 ppm.

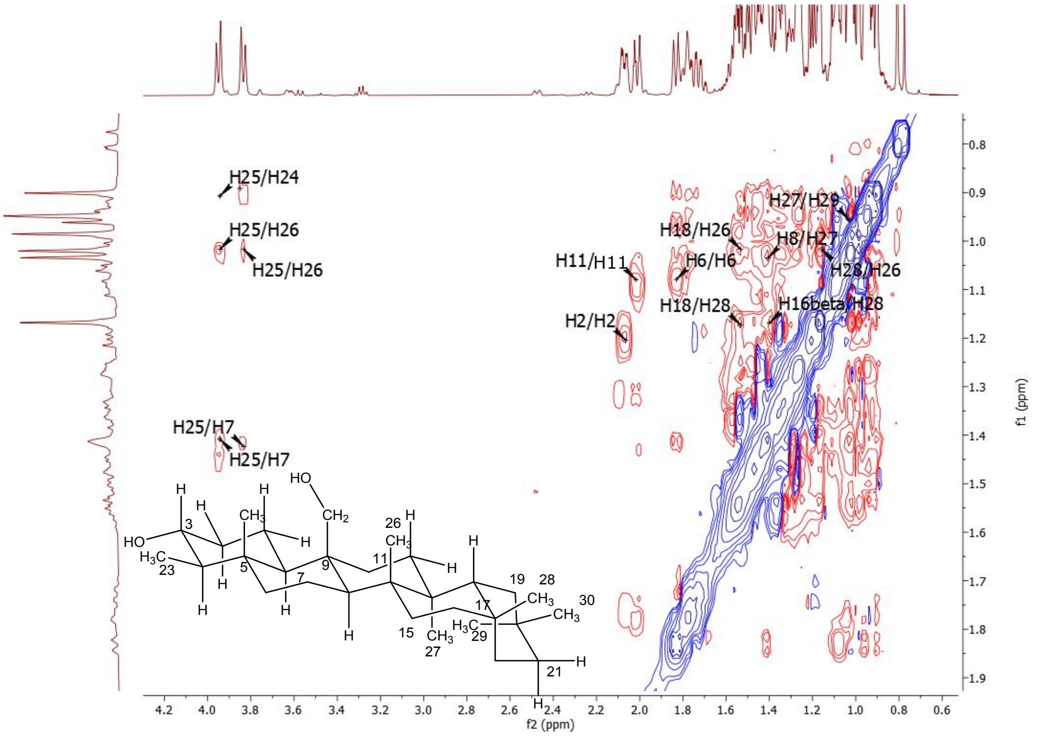

Figure S71: NOESY (600 MHz. $CDCl_3 + PyD_5$) spectrum of **15**.

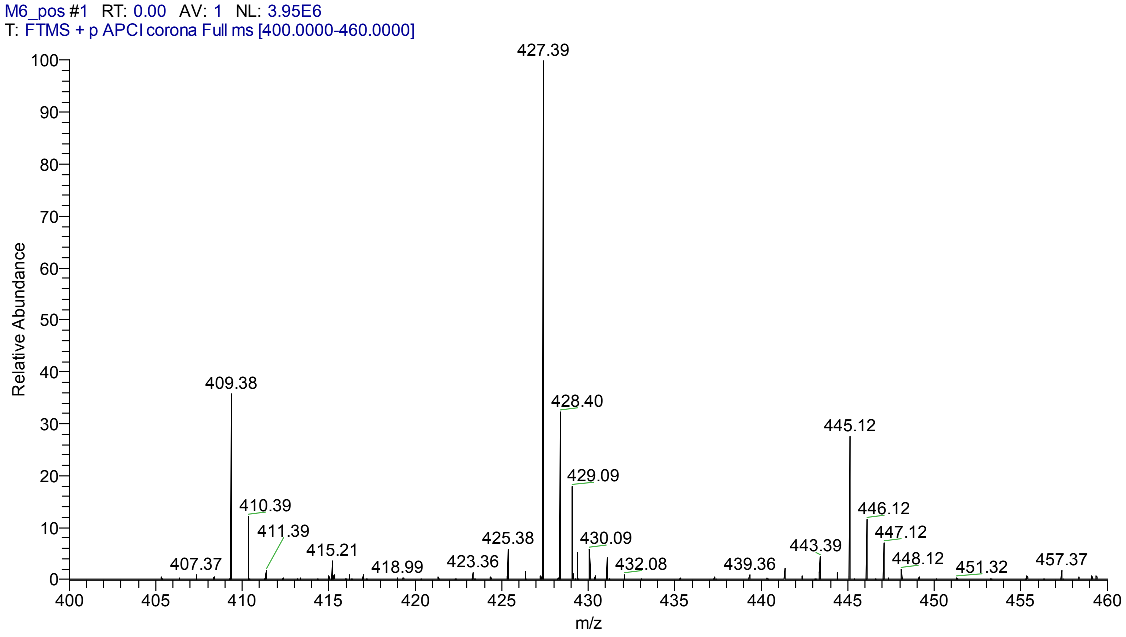

Figure S72: Mass spectrum in positive mode (HR-APCI-MS) of compound **15**.

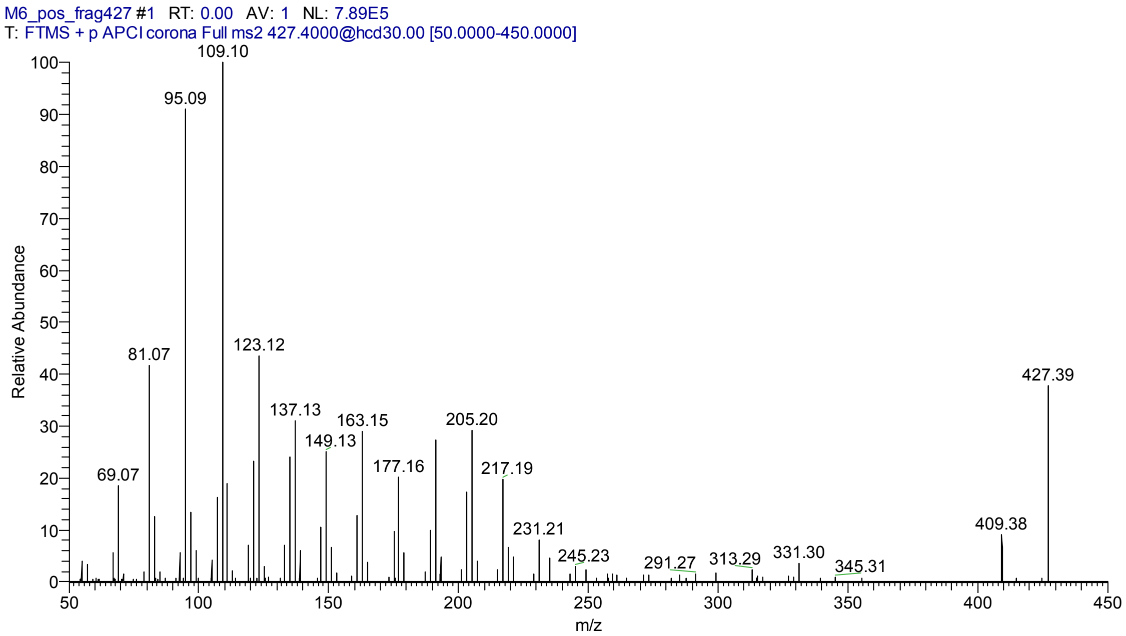

Figure S73: MS^2 spectrum of the 427 *m/z* ion fragmentation (HR-ESI-MS) of compound 15.

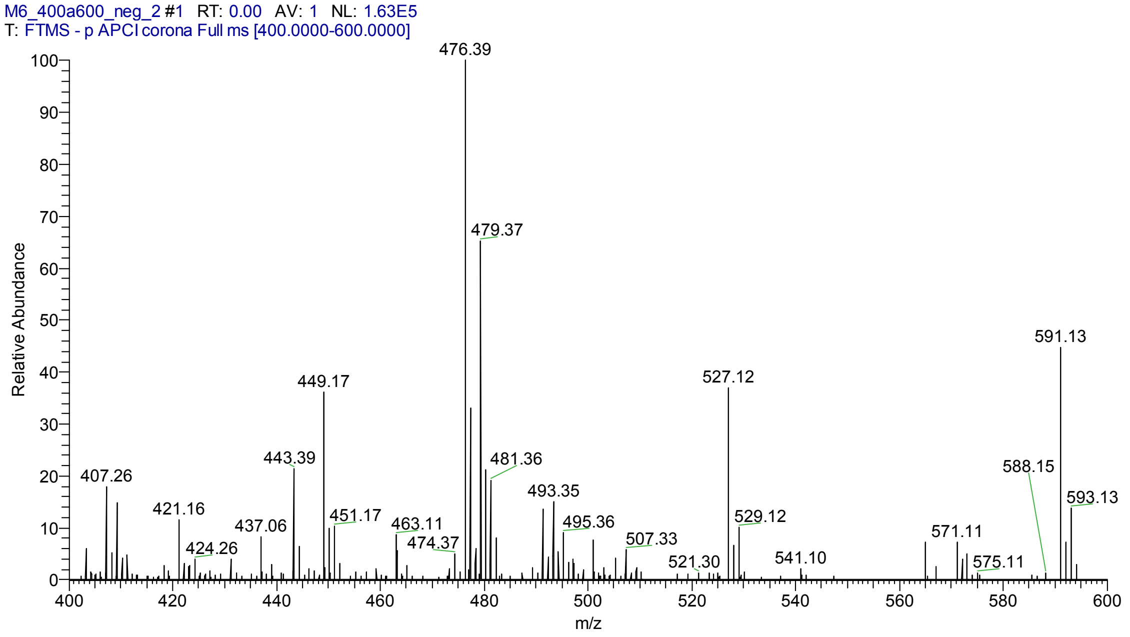

Figure S74: Mass spectrum in negative mode (HR-APCI-MS) of compound **15**.

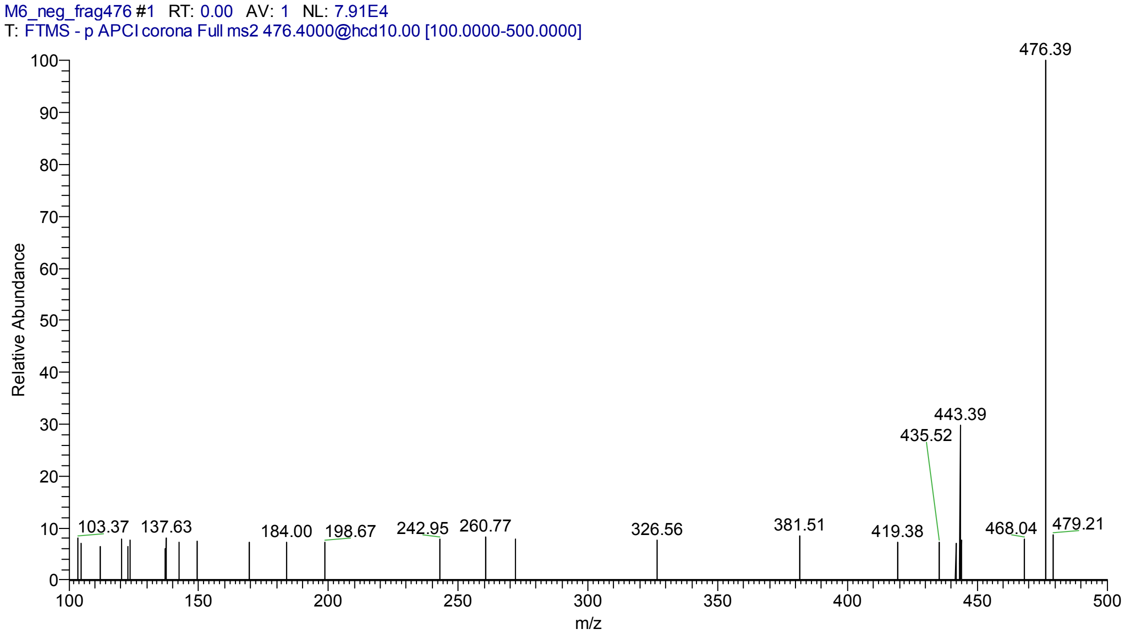

Figure S75: MS^2 spectrum of the 476 *m/z* ion fragmentation (HR-ESI-MS) of compound **15**.

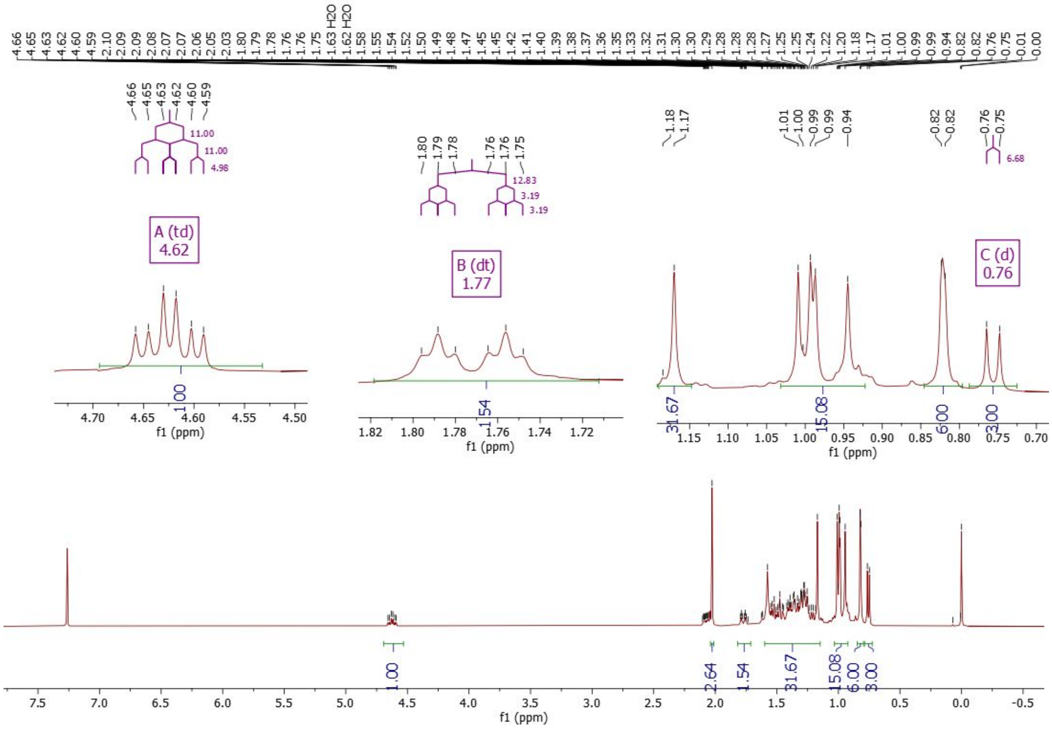

Figure S76: ^{1}H NMR spectrum (400 MHz. $CDCl_3$) of compound **16.**

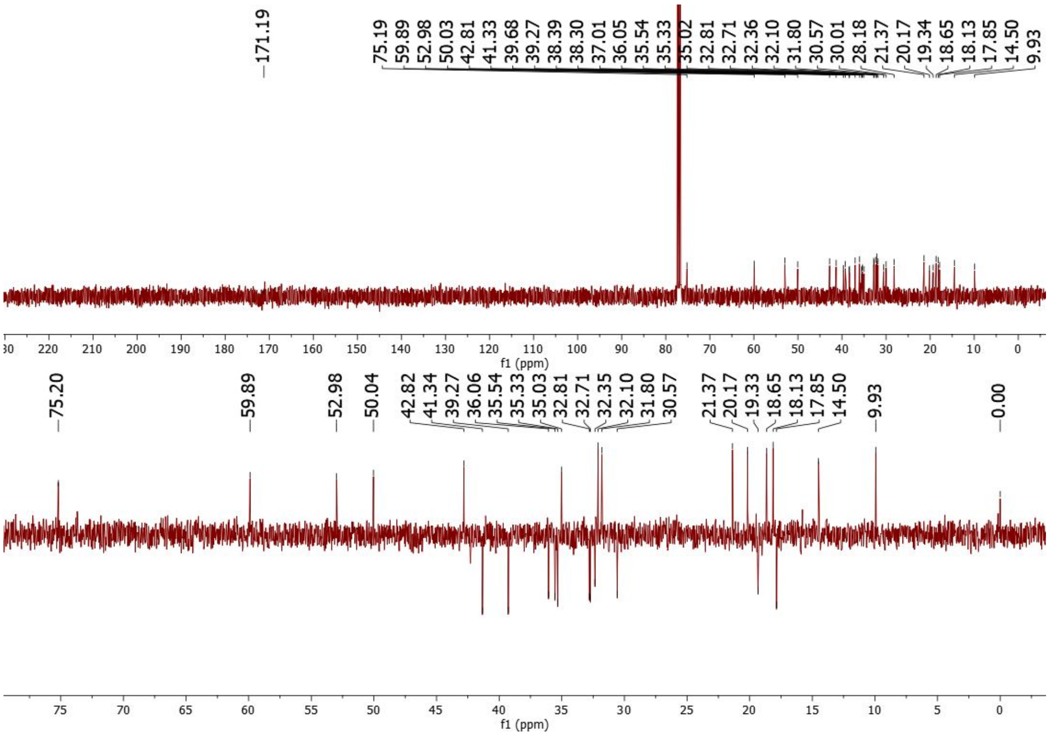

Figure S77: ^{13}C NMR and DEPT-135 spectra (100 MHz. $CDCl_3$) of compound **16.**

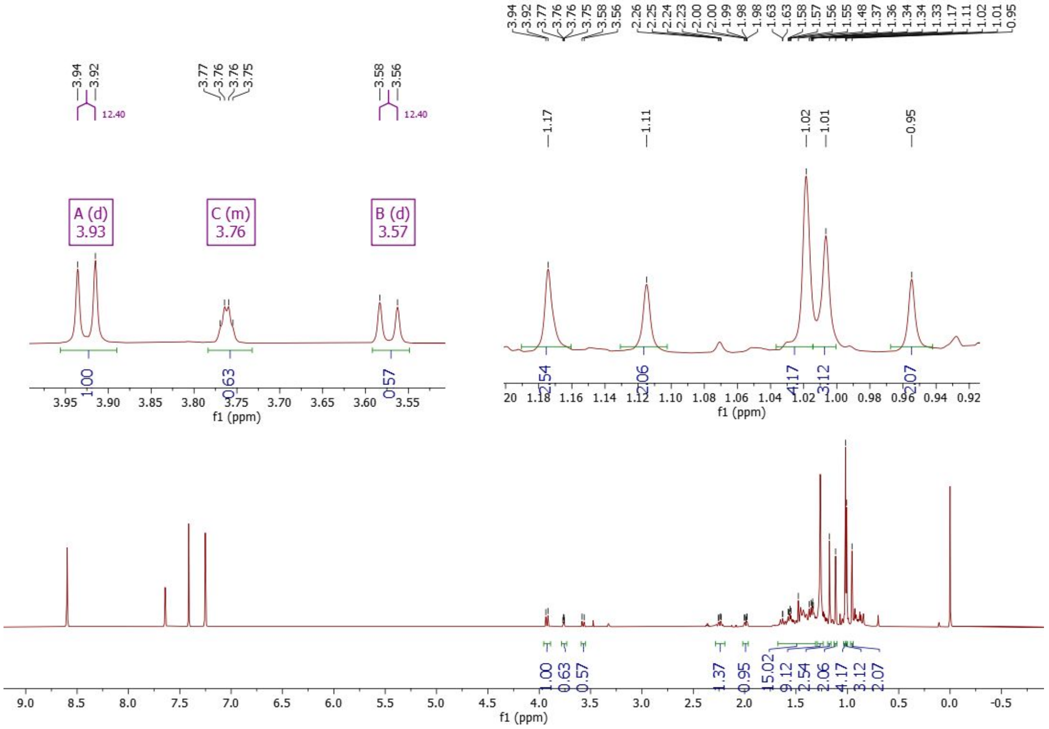

Figure S78: ^{1}H NMR spectrum (600 MHz. $CDCl_3$ + PyD_5) of compound **17.**

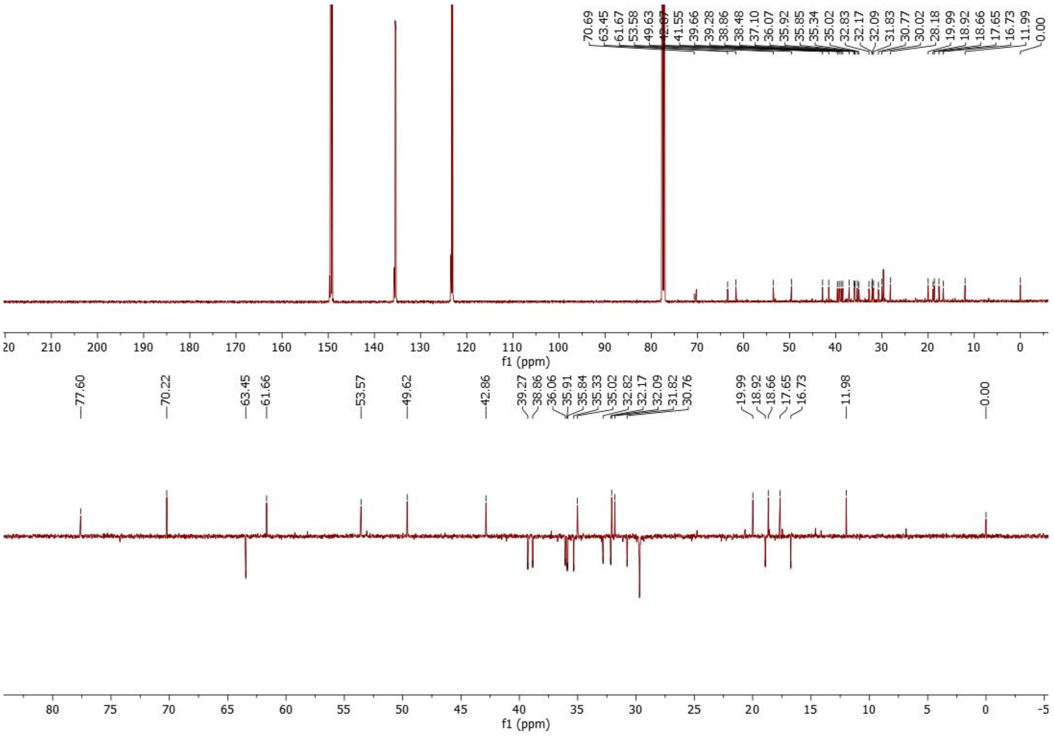

Figure S79: ^{13}C NMR and DEPT-135 spectra (150 MHz. $CDCl_3$ + PyD_5) of compound **17.**

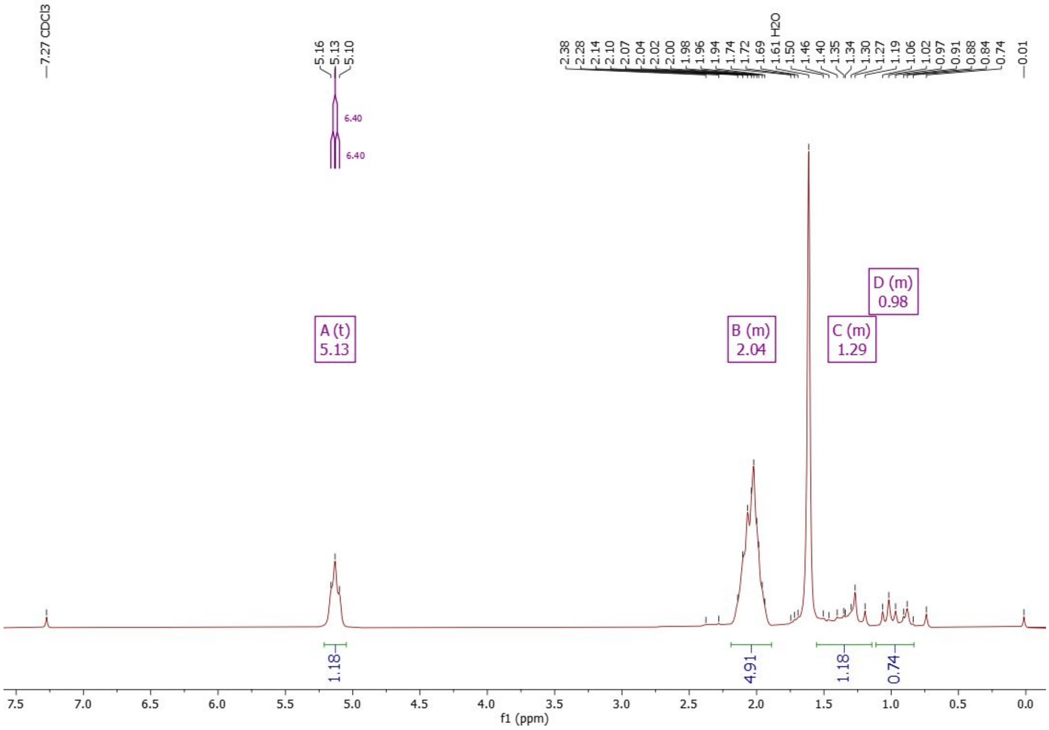

Figure S80: ^{1}H NMR spectrum (200 MHz. $CDCl_3$) of gutta-percha**.**

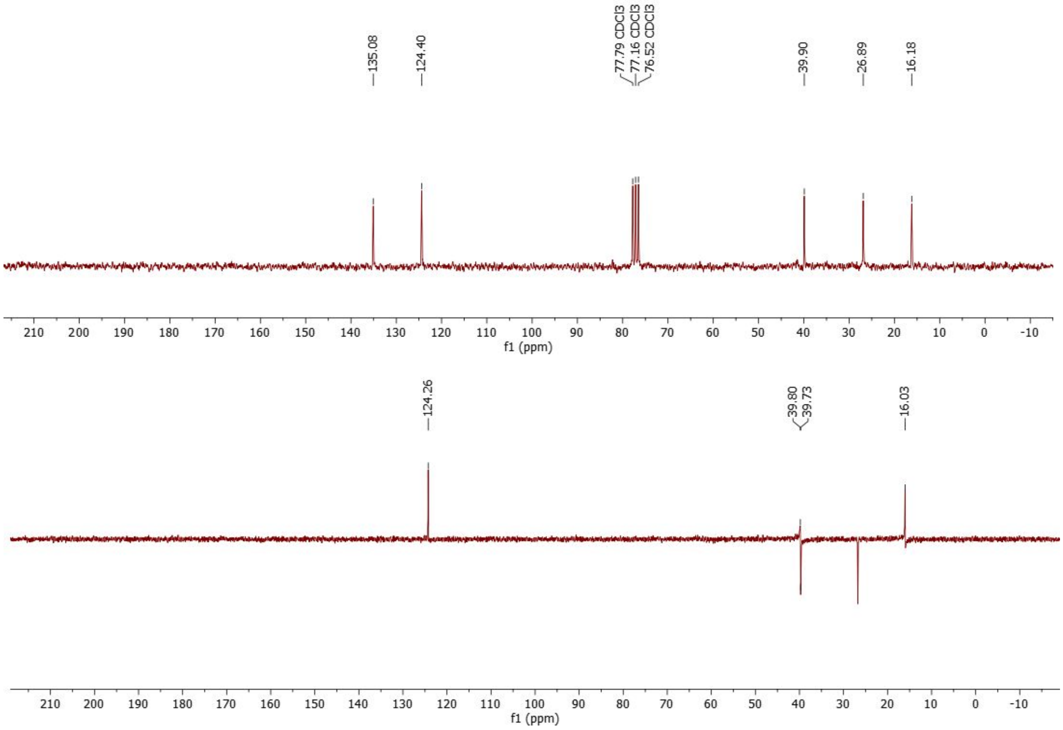

Figure S81: ^{13}C NMR and DEPT-135 spectra (50 MHz. $CDCl_3$) of gutta-percha**.**
